# Supplementary material for: Metabolites from the Paracel Islands Soft Coral Sinularia cf. molesta
Source: Mar Drugs. 2018 Dec 19;16(12):517. doi: 10.3390/md16120517 (PMC6317055; doi:10.3390/md16120517)
Supplement: Supplementary file 1 [file marinedrugs-16-00517-s001.pdf]

Supplementary Materials

Metabolites from the Paracel Islands Soft Coral *Sinularia* cf. *molesta*

Mei-Jun Chu [1,2,†], Xu-Li Tang [3,†], Xiao Han [1,4], Tao Li [1,4], Xiang-Chao Luo [1,4], Ming-Ming Jiang [1,4], Leen van Ofwegen [5], Lian-Zhong Luo [6], Gang Zhang [6], Ping-Lin Li [1,4,*] and Guo-Qiang Li [1,4,*]

1 Key Laboratory of Marine Drugs, Chinese Ministry of Education, School of Medicine and Pharmacy, Ocean University of China, Qingdao 266003, China; chumjun@163.com (M.-J.C.); 18765422150@163.com (X.H.); bruce13@163.com (T.L.); luoxc981@163.com (X.-C.L.); jiangmingming1989@126.com (M.-M.J.)
2 Tobacco Research Institute, Chinese Academy of Agricultural Sciences, Qingdao 266101, China
3 College of Chemistry and Chemical Engineering, Ocean University of China, Qingdao 266100, China; tangxuli@ouc.edu.cn
4 Laboratory of Marine Drugs and Biological Products, National Laboratory for Marine Science and Technology, Qingdao 266235, China
5 Nationaal Natuurhistorisch Museum, 2300 RA Leiden, The Netherlands; ofwegen@yahoo.com
6 Xiamen Key Laboratory of Marine Medicinal Natural Products Resources, Xiamen Medical College, Xiamen 361023, China; lzluo@xmu.edu.cn (L.-Z.L.); zg@xmmc.edu.cn (G.Z.)
* Correspondence: lipinglin@ouc.edu.cn (P.-L.L.); liguoqiang@ouc.edu.cn (G.-Q.L.); Tel.: +86-532-82032323 (G.-Q.L.)
† These authors contributed equally to this paper.

Contents

NMR and HRESIMS spectra of the new compounds

Figure S1. ^{1}H NMR (500 MHz, $CDCl_3$) spectrum of **1** **1**
Figure S2. ^{13}C NMR (125 MHz, $CDCl_3$) spectrum of **1** **1**
Figure S3. DEPT (125 MHz, $CDCl_3$) spectrum of **1** **2**
Figure S4. ^{1}H-^{1}H COSY ($CDCl_3$) spectrum of **1** **2**
Figure S5. HSQC ($CDCl_3$) spectrum of **1** **3**
Figure S6. HMBC ($CDCl_3$) spectrum of **1** **3**
Figure S7. NOESY ($CDCl_3$) spectrum of **1** **4**
Figure S8. HRESIMS spectrum of **1** **4**
Figure S9. ^{1}H NMR (500 MHz, CD_3OD) spectrum of **2** **5**
Figure S10. ^{1}H NMR (500 MHz, $CDCl_3$) spectrum of **2** **5**
Figure S11. ^{13}C NMR (125 MHz, CD_3OD) spectrum of **2** **6**
Figure S12. DEPT (125 MHz, CD_3OD) spectrum of **2** **6**
Figure S13. ^{1}H-^{1}H COSY (CD_3OD) spectrum of **2** **7**
Figure S14. HSQC (CD_3OD) spectrum of **2** **7**
Figure S15. HMBC (CD_3OD) spectrum of **2** **8**
Figure S16. NOESY (CD_3OD) spectrum of **2** **8**
Figure S17. HRESIMS spectrum of **2** **9**
Figure S18. ^{1}H NMR (500 MHz, $CDCl_3$) spectrum of **3** **9**
Figure S19. ^{13}C NMR (125 MHz, $CDCl_3$) spectrum of **3** **10**
Figure S20. DEPT (125 MHz, $CDCl_3$) spectrum of **3** **10**
Figure S21. ^{1}H-^{1}H COSY ($CDCl_3$) spectrum of **3** **11**
Figure S22. HMBC ($CDCl_3$) spectrum of **3** **11**
Figure S23. NOESY ($CDCl_3$) spectrum of **3** **12**
Figure S24. HRESIMS spectrum of **3** **12**
Figure S25. ^{1}H NMR (500 MHz, $CDCl_3$) spectrum of **4** **13**
Figure S26. ^{13}C NMR (125 MHz, $CDCl_3$) spectrum of **4** **13**
Figure S27. DEPT (125 MHz, $CDCl_3$) spectrum of **4** **14**
Figure S28. ^{1}H-^{1}H COSY ($CDCl_3$) spectrum of **4** **14**
Figure S29. HMBC ($CDCl_3$) spectrum of **4** **15**
Figure S30. NOESY ($CDCl_3$) spectrum of **4** **15**
Figure S31. HRESIMS spectrum of **4** **16**
Figure S32. ^{1}H NMR ($CDCl_3$, 500 MHz) spectrum of **5** **16**
Figure S33. ^{13}C NMR ($CDCl_3$, 125 MHz) spectrum of **5** **17**
Figure S34. DEPT ($CDCl_3$, 125 MHz) spectrum of **5** **17**
Figure S35. ^{1}H-^{1}H COSY ($CDCl_3$) spectrum of **5** **18**
Figure S36. HSQC ($CDCl_3$) spectrum of **5** **18**
Figure S37. HMBC ($CDCl_3$) spectrum of **5** **19**
Figure S38. NOESY ($CDCl_3$) spectrum of **5** **19**
Figure S39. HRESIMS spectrum of **5** **20**
Figure S40. ^{1}H NMR ($CDCl_3$, 500 MHz) spectrum of (+)**-9** **20**

**Figure S41.** ^{13}C NMR ($CDCl_3$, 125 MHz) spectrum of (+)-**9**	**21**
**Figure S42.** DEPT ($CDCl_3$, 125 MHz) spectrum of (+)-**9**	**21**
**Figure S43.** ^{1}H-^{1}H COSY ($CDCl_3$) spectrum of (+)-**9**	**22**
**Figure S44.** HSQC ($CDCl_3$) spectrum of (+)-**9**	**22**
**Figure S45.** HMBC ($CDCl_3$) spectrum of (+)-**9**	**23**
**Figure S46.** NOESY ($CDCl_3$) spectrum of (+)-**9**	**23**
**Figure S47.** HRESIMS spectrum of (+)-**9**	**24**
**Figure S48.** ^{1}H NMR ($CDCl_3$, 500 MHz) spectrum of (+)-**10**	**24**
**Figure S49.** ^{13}C NMR ($CDCl_3$, 125 MHz) spectrum of (+)-**10**	**25**
**Figure S50.** DEPT ($CDCl_3$, 125 MHz) spectrum of (+)-**10**	**25**
**Figure S51.** ^{1}H-^{1}H COSY ($CDCl_3$) spectrum of (+)-**10**	**26**
**Figure S52.** HSQC ($CDCl_3$) spectrum of (+)-**10**	**26**
**Figure S53.** HMBC ($CDCl_3$) spectrum of (+)-**10**	**27**
**Figure S54.** NOESY ($CDCl_3$) spectrum of (+)-**10**	**27**
**Figure S55.** HRESIMS spectrum of (+)-**10**	**28**
**Figure S56.** ^{1}H NMR ($CDCl_3$, 500 MHz) spectrum of (+)-**11**	**28**
**Figure S57.** ^{13}C NMR ($CDCl_3$, 125 MHz) spectrum of (+)-**11**	**29**
**Figure S58.** DEPT ($CDCl_3$, 125 MHz) spectrum of (+)-**11**	**29**
**Figure S59.** ^{1}H-^{1}H COSY ($CDCl_3$) spectrum of (+)-**11**	**30**
**Figure S60.** HSQC ($CDCl_3$) spectrum of (+)-**11**	**30**
**Figure S61.** HMBC ($CDCl_3$) spectrum of (+)-**11**	**31**
**Figure S62.** NOESY ($CDCl_3$) spectrum of (+)-**11**	**31**
**Figure S63.** HRESIMS spectrum of (+)-**11**	**32**
**Figure S64.** ^{1}H NMR ($CDCl_3$, 500 MHz) spectrum of (+)-**13**	**32**
**Figure S65.** ^{13}C NMR ($CDCl_3$, 125 MHz) spectrum of (+)-**13**	**33**
**Figure S66.** DEPT ($CDCl_3$, 125 MHz) spectrum of (+)-**13**	**33**
**Figure S67.** ^{1}H-^{1}H COSY ($CDCl_3$) spectrum of (+)-**13**	**34**
**Figure S68.** HSQC ($CDCl_3$) spectrum of (+)-**13**	**34**
**Figure S69.** HMBC ($CDCl_3$) spectrum of (+)-**13**	**35**
**Figure S70.** HRESIMS spectrum of (+)-**13**	**35**
**Figure S71.** ^{1}H NMR ($CDCl_3$, 500 MHz) spectrum of **14**	**36**
**Figure S72.** ^{13}C NMR ($CDCl_3$, 125 MHz) spectrum of **14**	**36**
**Figure S73.** DEPT ($CDCl_3$, 125 MHz) spectrum of **14**	**37**
**Figure S74.** ^{1}H-^{1}H COSY ($CDCl_3$) spectrum of **14**	**37**
**Figure S75.** HSQC ($CDCl_3$) spectrum of **14**	**38**
**Figure S76.** HMBC ($CDCl_3$) spectrum of **14**	**38**
**Figure S77.** NOESY ($CDCl_3$) spectrum of **14**	**39**
**Figure S78.** HRESIMS spectrum of **14**	**39**
Chiral HPLC chromatograms of compounds (±)-**9**–(±)-**13**	
**Figure S79.** Chiral HPLC (n-hexane/i-PrOH 80:20) chromatogram of (+)-**9** and (–)-**9**	**40**
**Figure S80.** Chiral HPLC (n-hexane/i-PrOH 90:10) chromatogram of (+)-**10** and (–)-**10**	**40**
**Figure S81.** Chiral HPLC (n-hexane/i-PrOH 70:30) chromatogram of (+)-**11**	**41**

and (–)-**11**	
**Figure S82.** Chiral HPLC (n-hexane/i-PrOH 97:3) chromatogram of (+)-**12** and (–)-**12**	**41**
**Figure S83.** Chiral HPLC (n-hexane/i-PrOH 85:15) chromatogram of (+)-**13** and (–)-**13**	**42**
Computational details of compounds **1−5**	
**Figure S84.** Stable conformers of compound **1** with 1*R*,10*R* (**1a**) and 1*S*,10*S* (**1b**) configurations, respectively	**44**
**Table S1.** Important thermodynamic parameters (a.u.) of the optimized compound **1** at B3LYP/6-31G (d,p) level in the gas phase	**44**
**Table S2.** Optimized Z-Matrixes of compound **1** in the Gas Phase (Å) at B3LYP/6-31G (d,p) level	**44**
**Figure S85.** Stable conformers of compound **2** with 1*R*,10*S* (**2a**) and 1*S*,10*R* (**2b**) configurations, respectively	**50**
**Table S3.** Important thermodynamic parameters (a.u.) of the optimized compound **2** at B3LYP/6-31G (d,p) level in the gas phase	**50**
**Table S4**. Optimized Z-Matrixes of compound **1** in the Gas Phase (Å) at B3LYP/6-31G (d,p) level.	**51**
**Figure S86.** Stable conformers of compound **3** with 1*S*,7*R*,10*S* (**3a**) and 1*R*,7*S*,10*R* (**3b**) configurations, respectively	**55**
**Table S5.** Important thermodynamic parameters (a.u.) of the optimized compound **3** at B3LYP/6-31G (d,p) level in the gas phase	**55**
**Table S6.** Optimized Z-Matrixes of compound **3** in the Gas Phase (Å) at B3LYP/6-31G (d,p) level	**56**
**Figure S87.** Stable conformers of compound **4** with 4*R*,10*R* (**4a**), 4*S*,10*S* (**4b**), 4*R*,10*S* (**4c**) and 4*S*,10*R* (**4d**) configurations, respectively	**60**
**Table S7.** Important thermodynamic parameters (a.u.) of the optimized compound **4** at B3LYP/6-31G (d,p) level in the gas phase	**60**
**Table S8.** Optimized Z-Matrixes of compound **4** in the Gas Phase (Å) at B3LYP/6-31G (d,p) level	**61**
**Figure S88.** Stable conformers of compound **5** with 1*R*,4*S*,10*S* (**5a**) and 1*S*,4*R*,10*R* (**5b**) configurations, respectively	**69**
**Table S9.** Important thermodynamic parameters (a.u.) of the optimized compound **5** at B3LYP/6-31G (d,p) level in the gas phase	**70**
**Table S10.** Optimized Z-Matrixes of compound **5** in the Gas Phase (Å) at B3LYP/6-31G (d,p) level	**70**

NMR and HRESIMS spectra of the new compounds

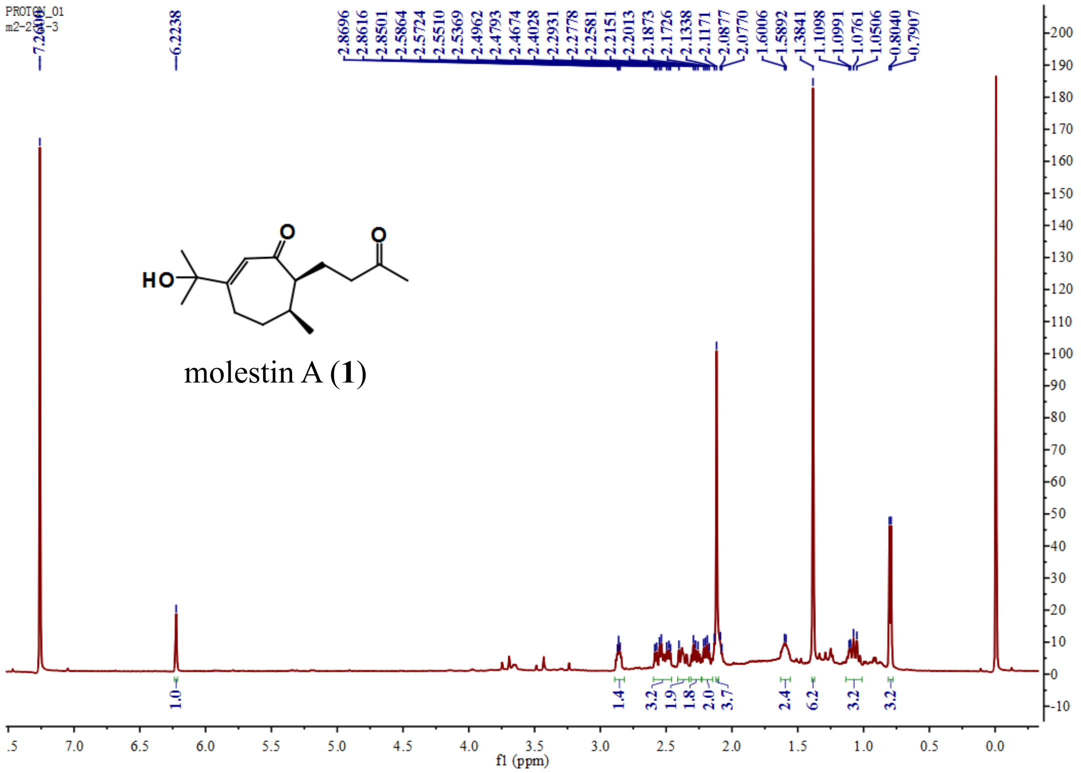

Figure S1. ^{1}H NMR (500 MHz, $CDCl_3$) spectrum of **1**

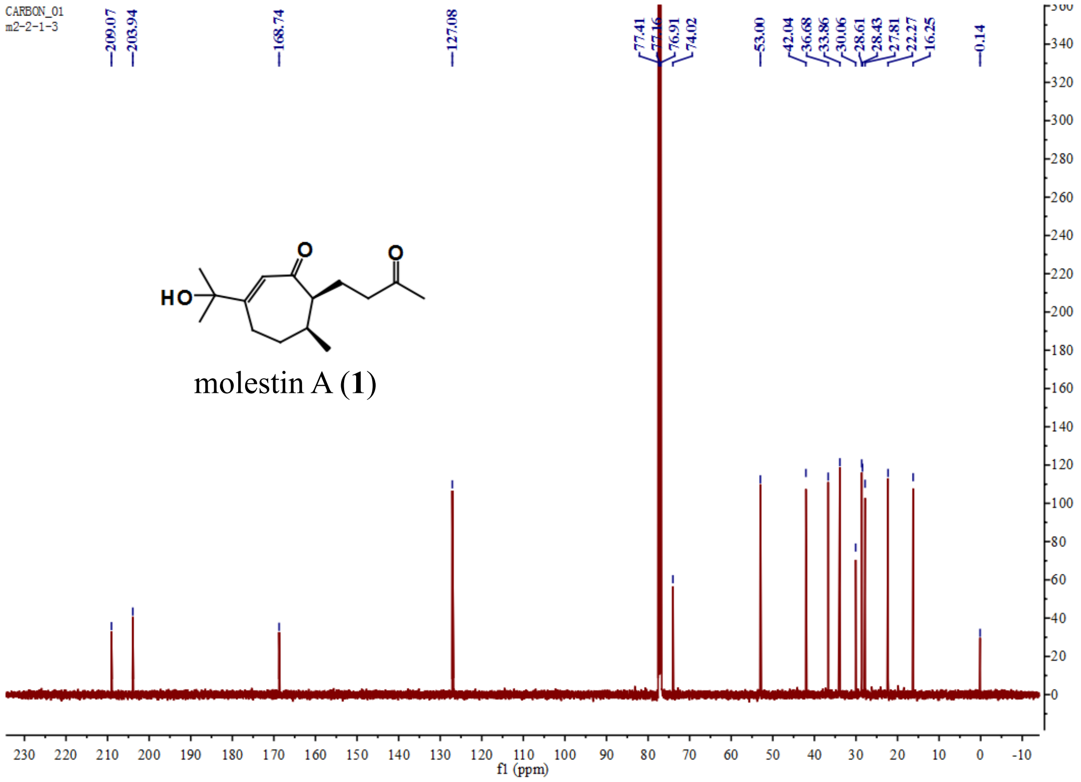

Figure S2. ^{13}C NMR (125 MHz, $CDCl_3$) spectrum of **1**

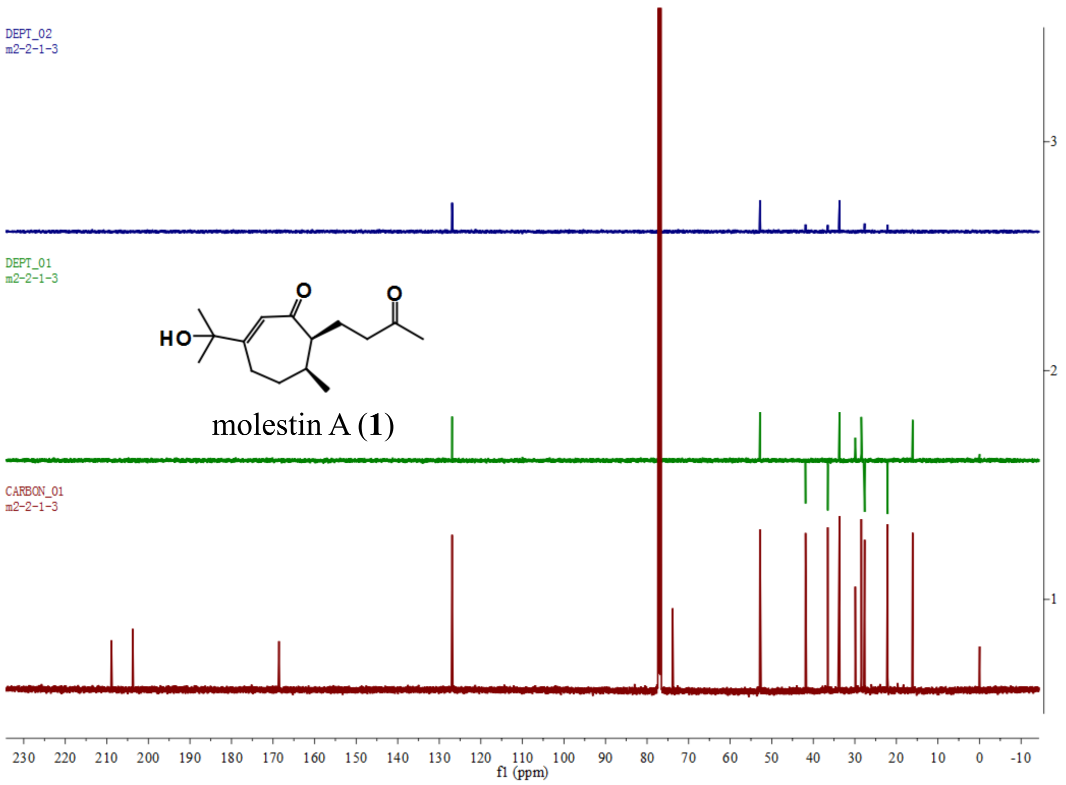

Figure S3. DEPT (125 MHz, $CDCl_3$) spectrum of **1**

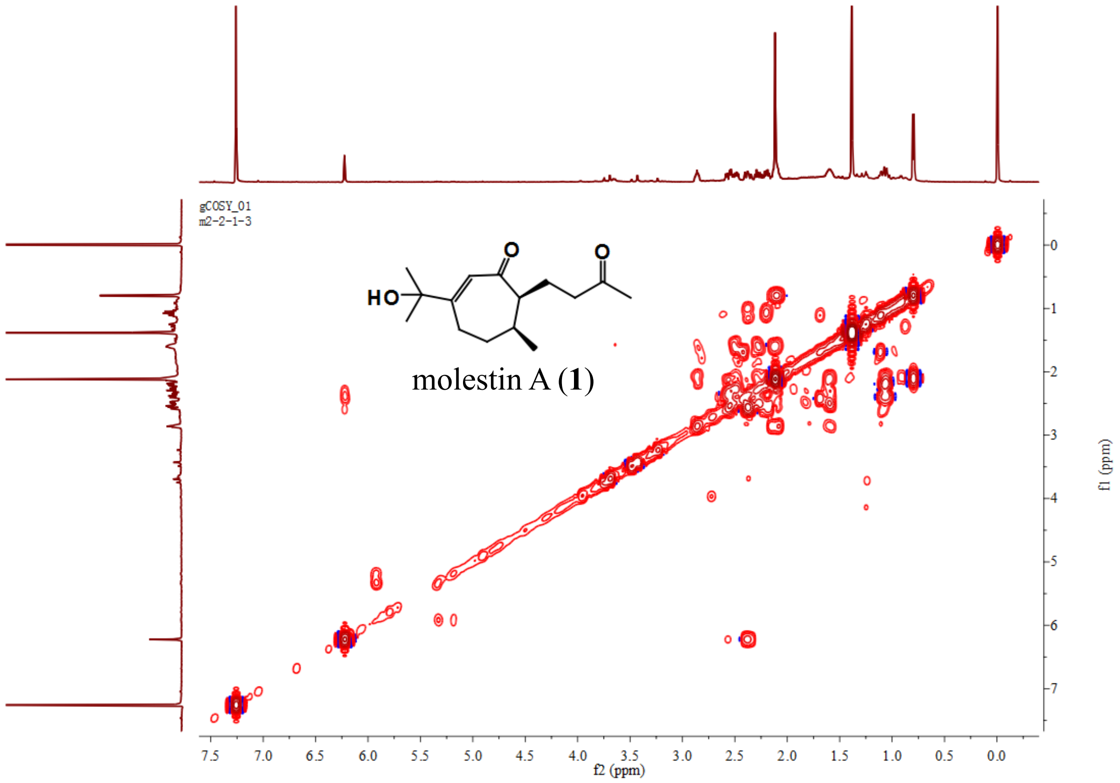

Figure S4. ^1H-^1H COSY ($CDCl_3$) spectrum of **1**

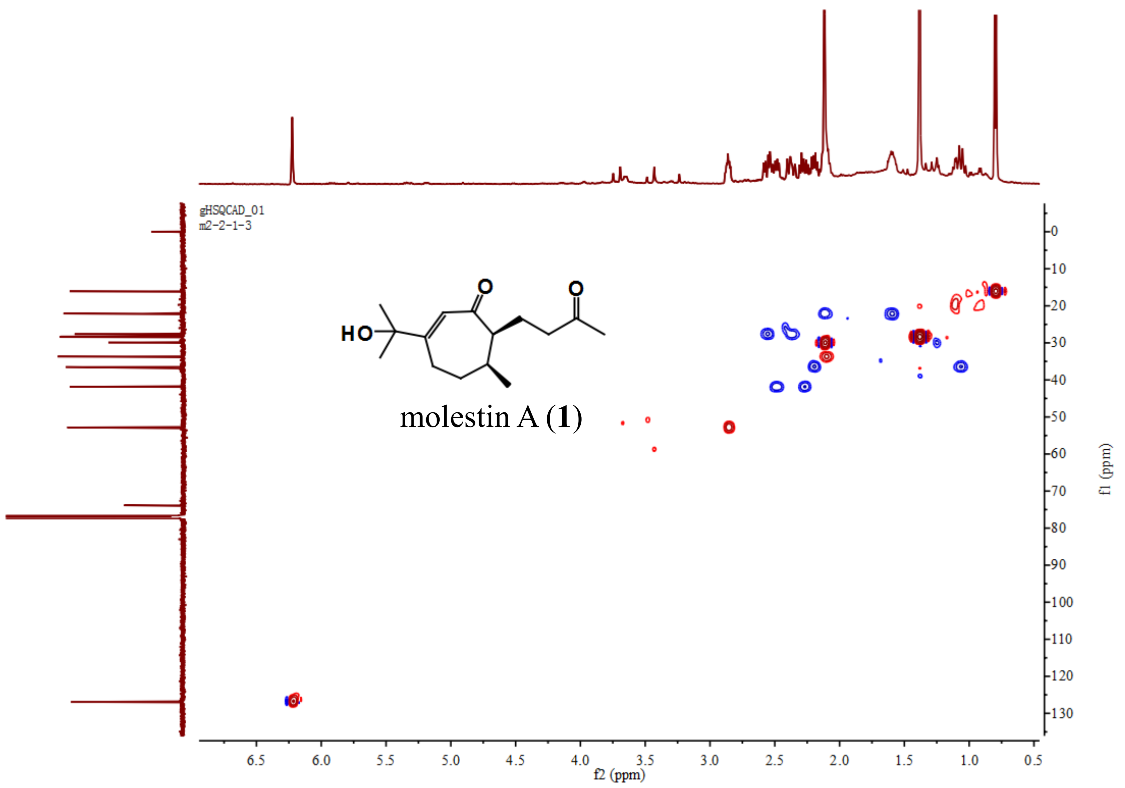

Figure S5. HSQC ($CDCl_3$) spectrum of **1**

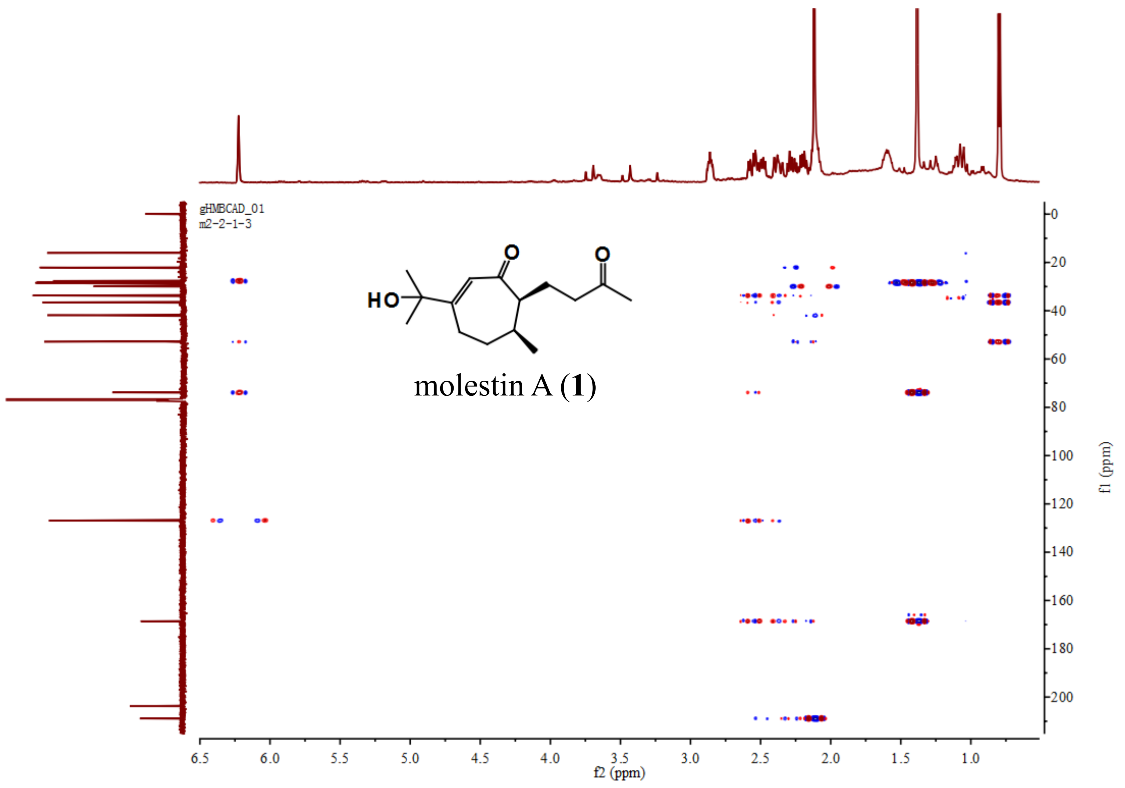

Figure S6. HMBC ($CDCl_3$) spectrum of **1**

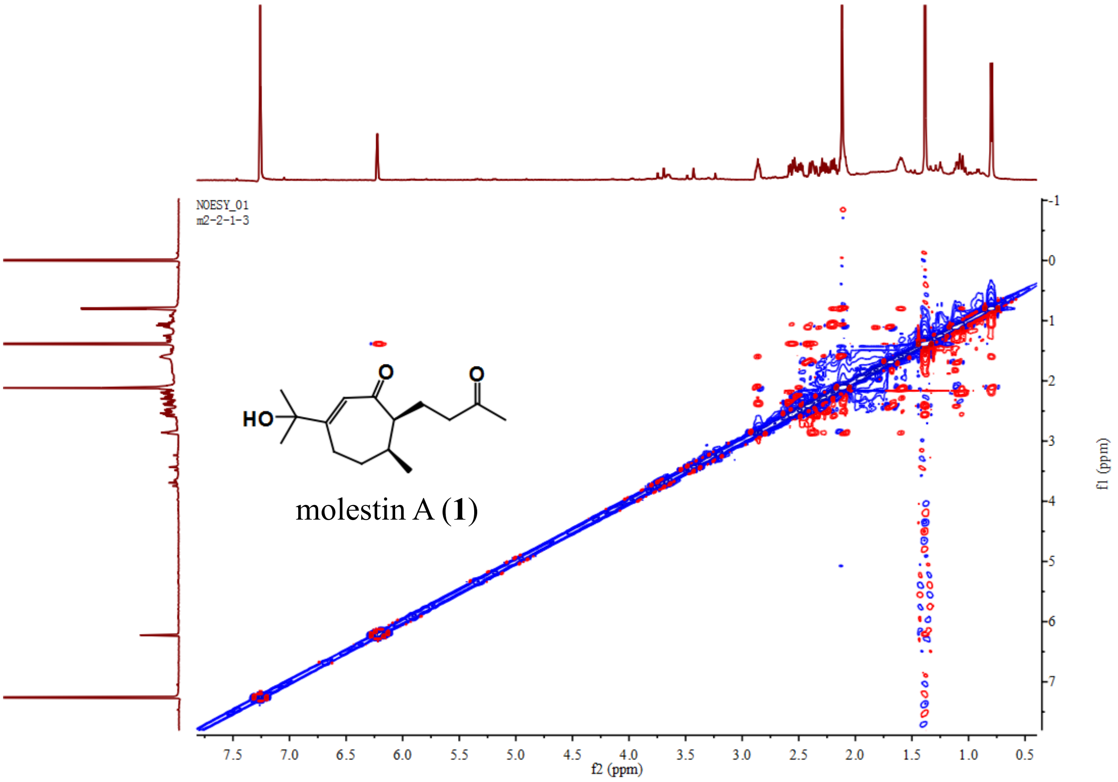

Figure S7. NOESY ($CDCl_3$) spectrum of **1**

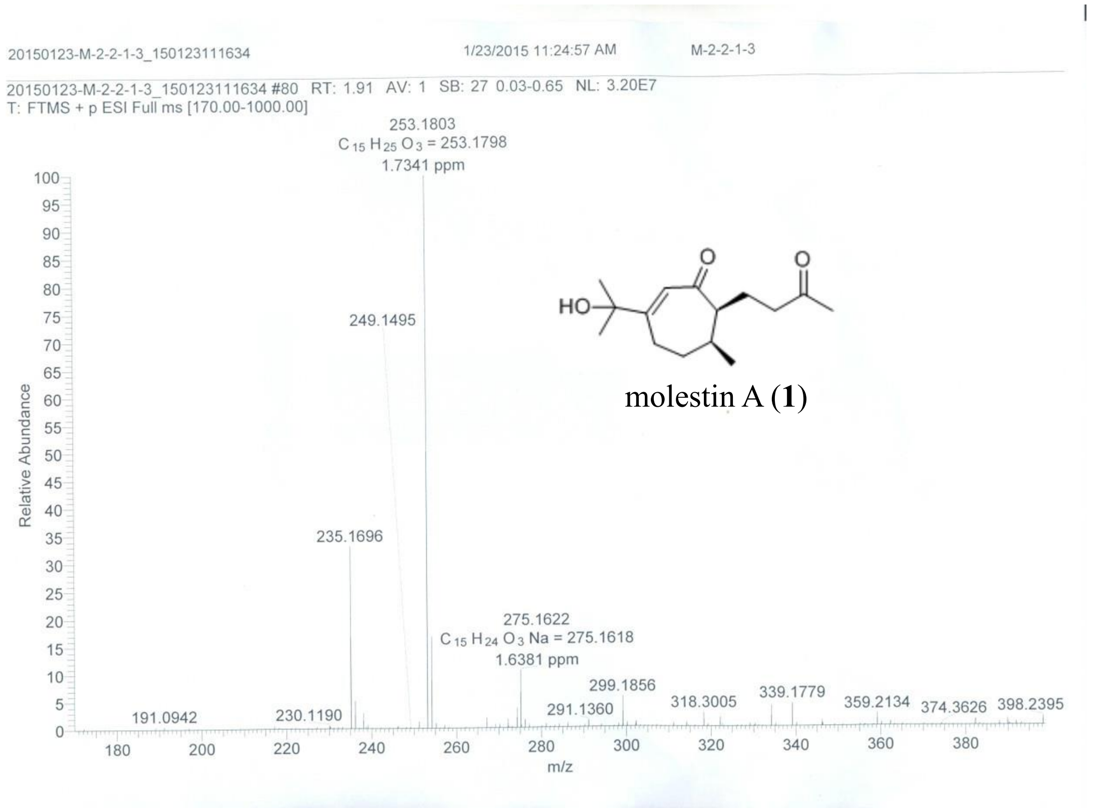

Figure S8. HRESIMS spectrum of **1**

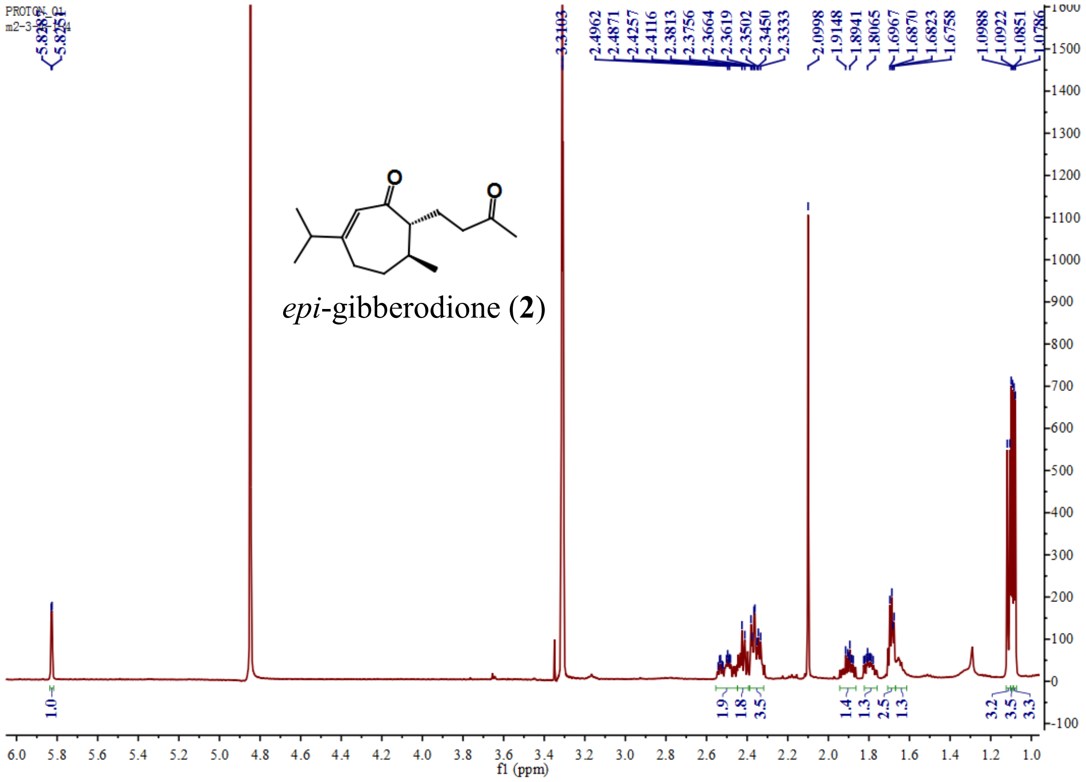

Figure S9. ^{1}H NMR (500 MHz, CD_3OD) spectrum of **2**

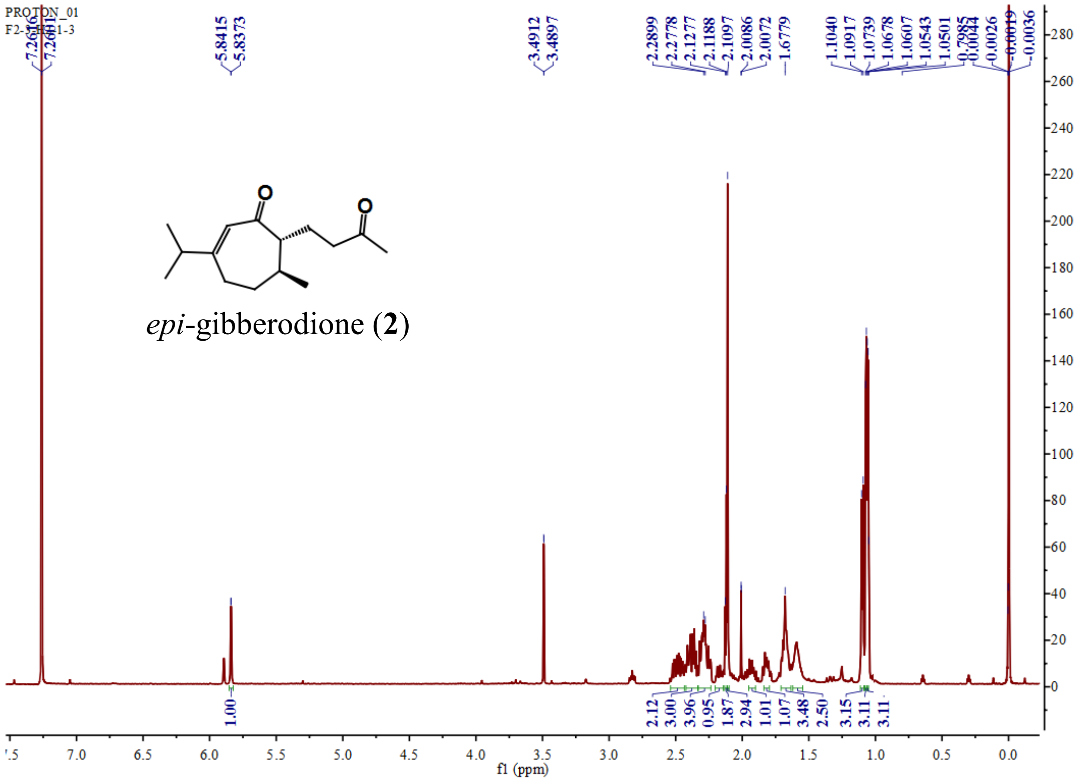

Figure S10. ^{1}H NMR (500 MHz, $CDCl_3$) spectrum of **2**

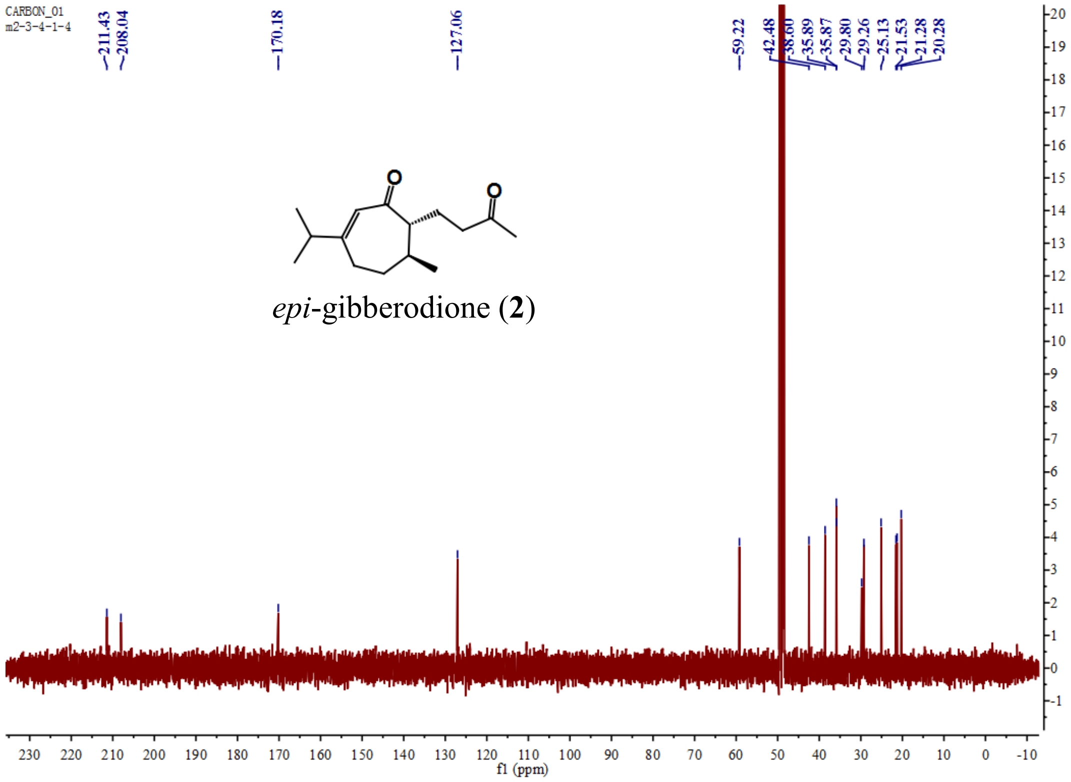

Figure S11. ^{13}C NMR (125 MHz, CD_3OD) spectrum of **2**

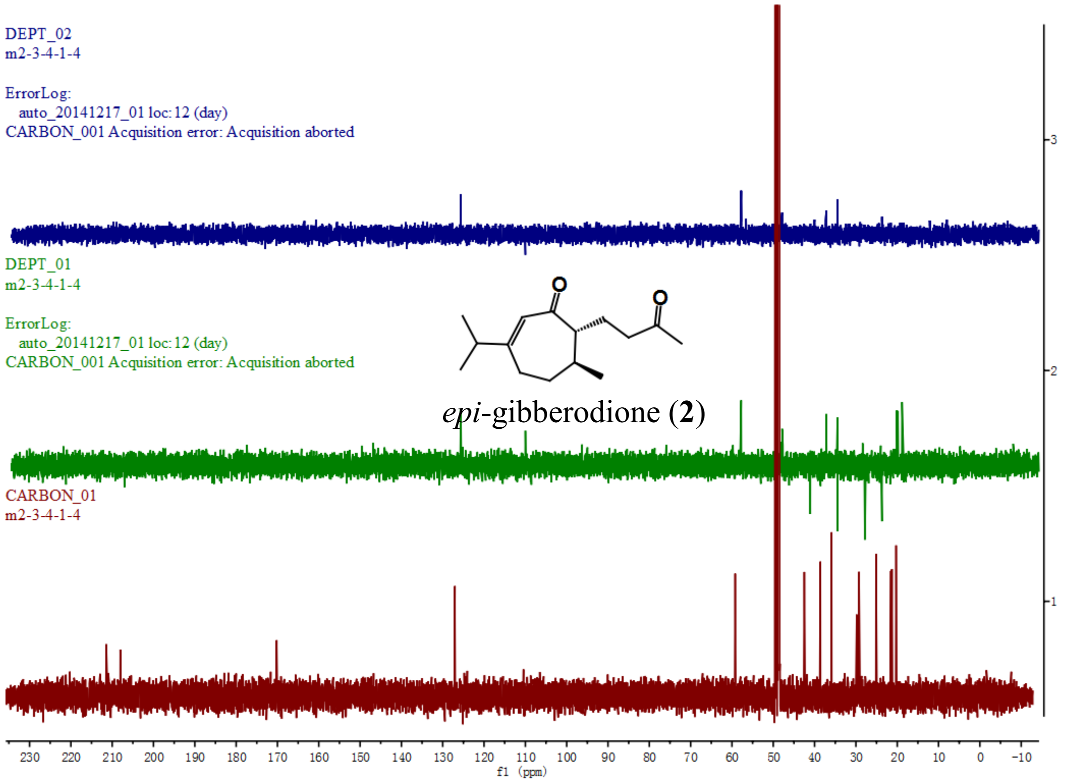

Figure S12. DEPT (125 MHz, CD_3OD) spectrum of **2**

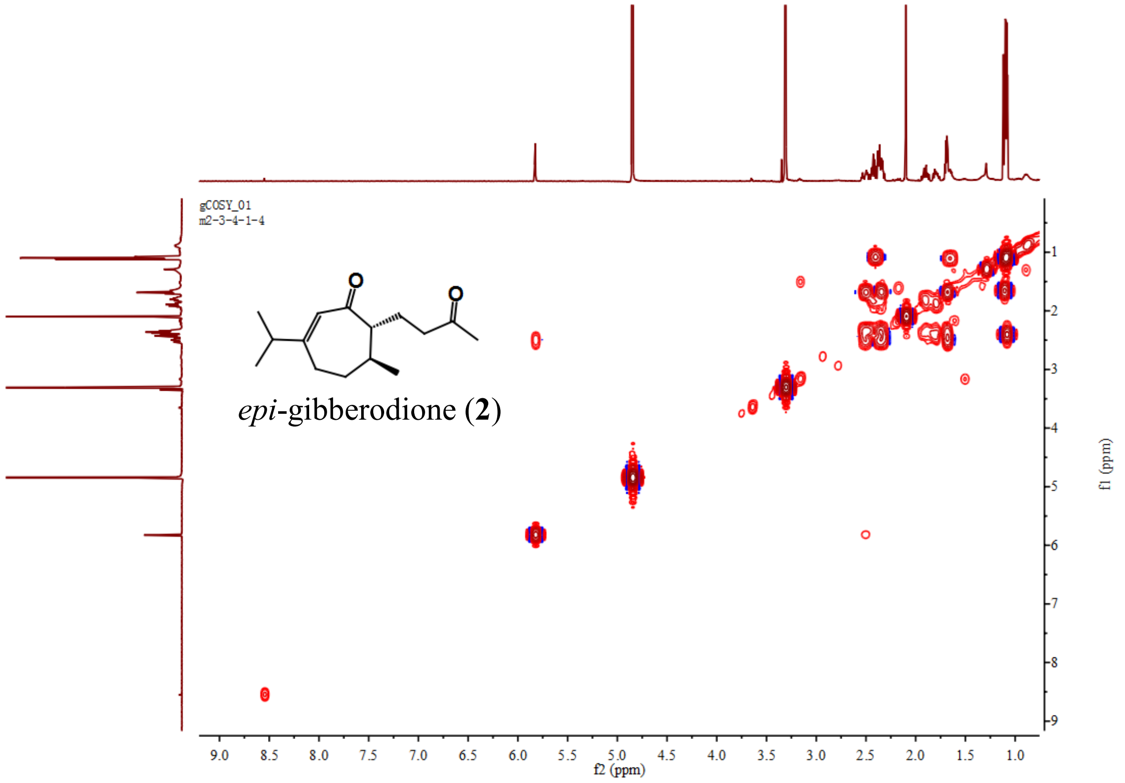

Figure S13. ^{1}H-^{1}H COSY (CD_3OD) spectrum of **2**

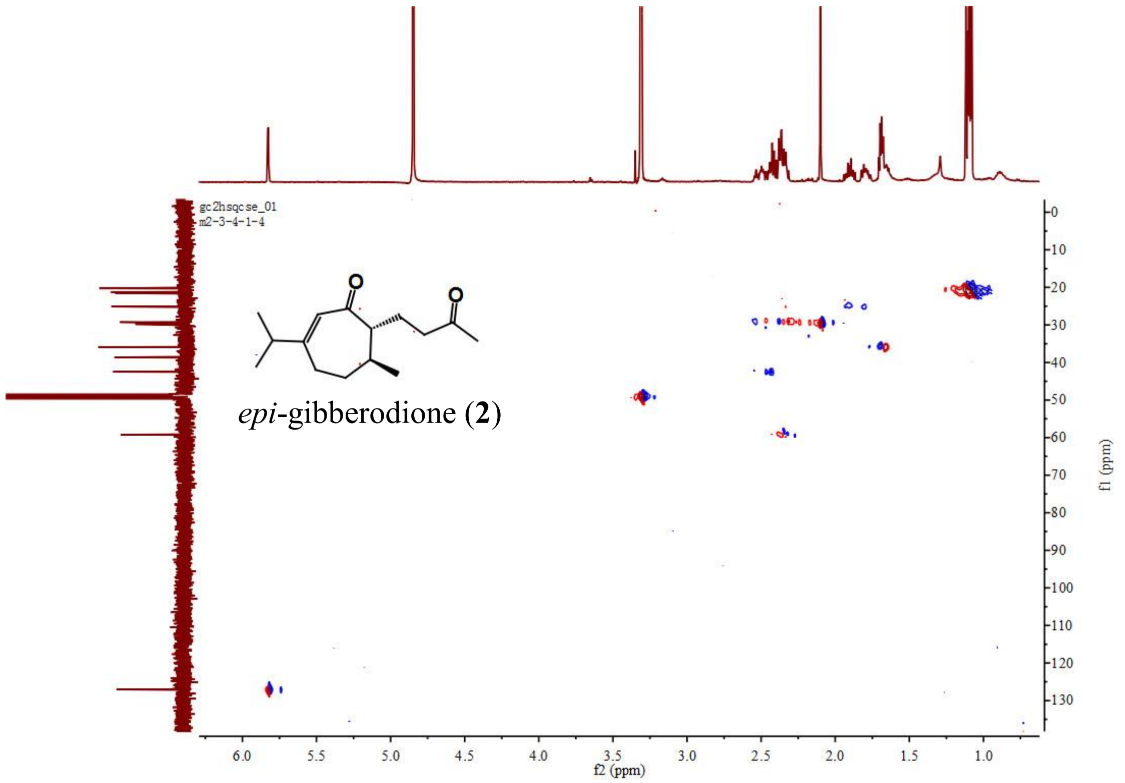

Figure S14. HSQC (CD_3OD) spectrum of **2**

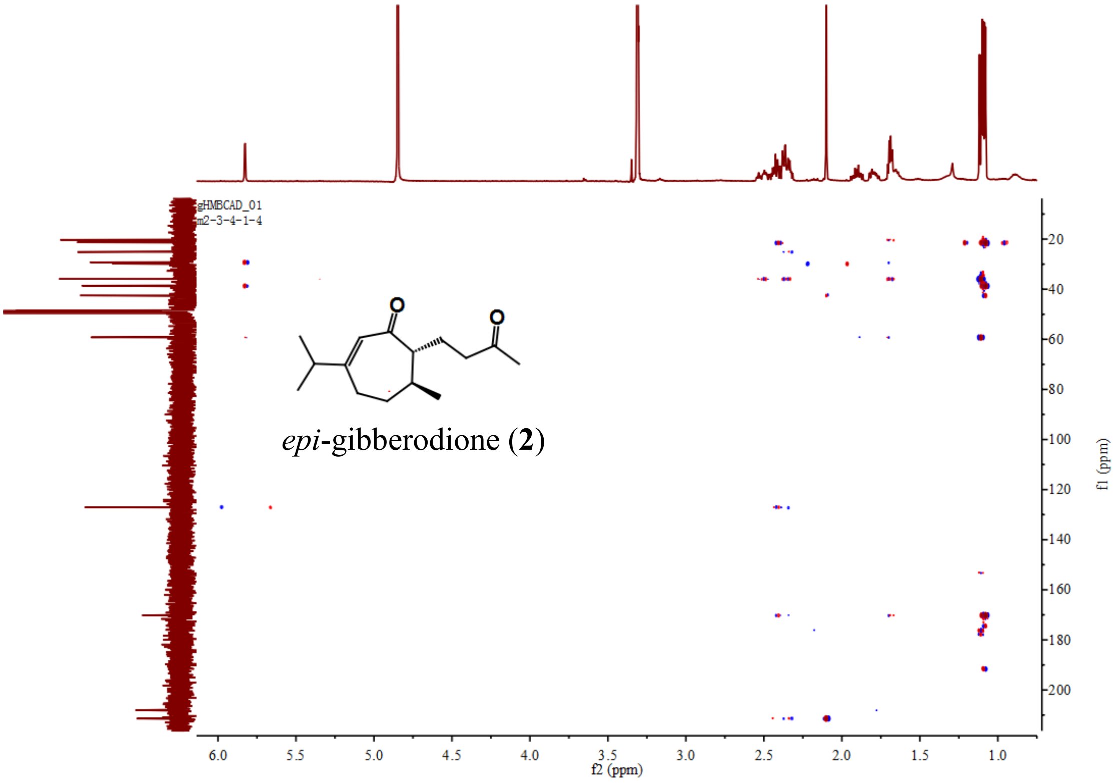

Figure S15. HMBC (CD_3OD) spectrum of **2**

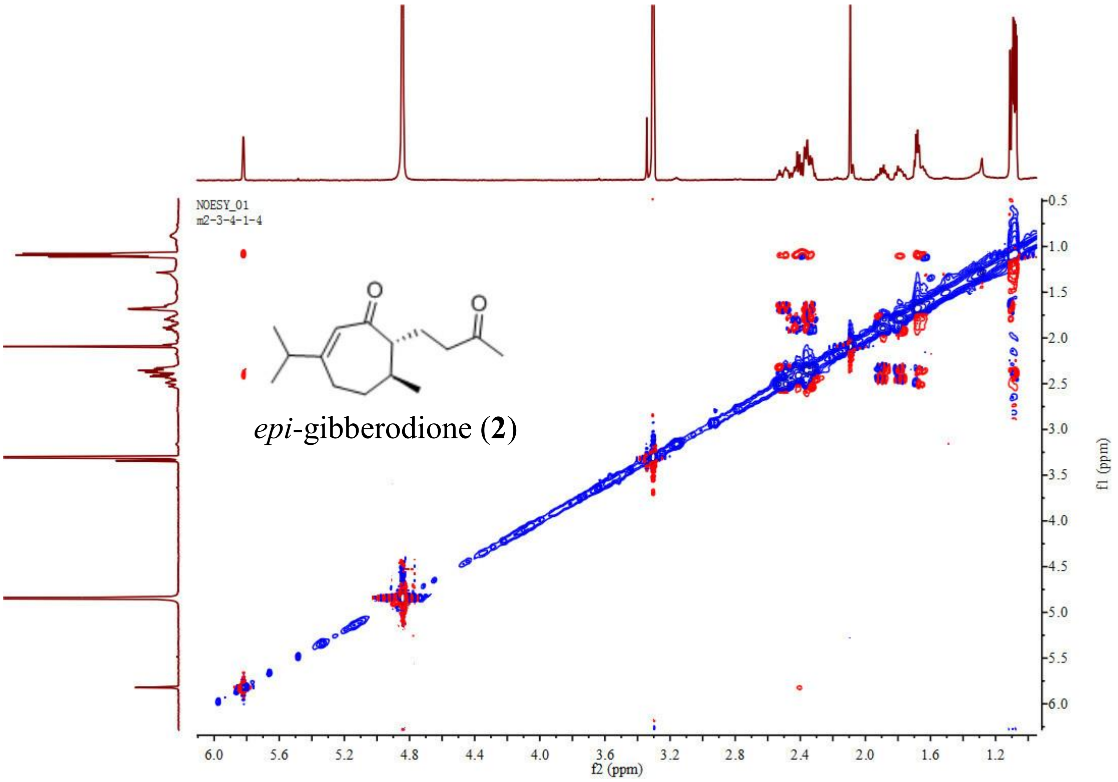

Figure S16. NOESY (CD_3OD) spectrum of **2**

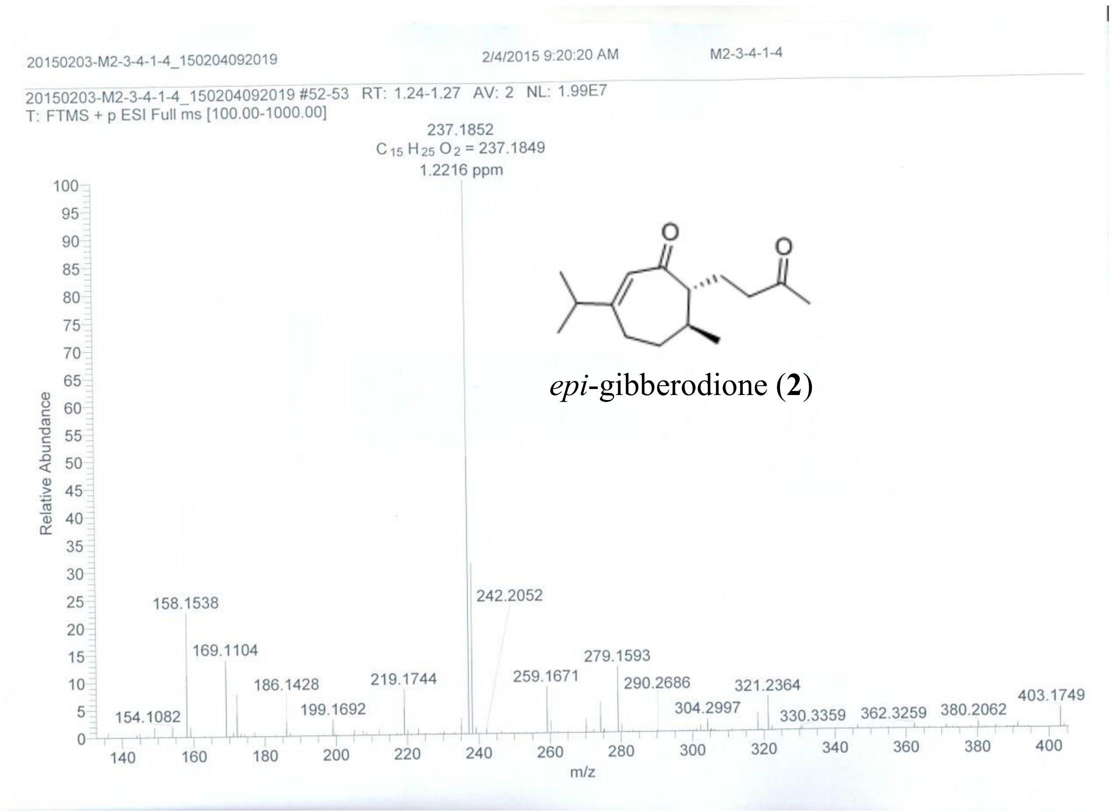

Figure S17. HRESIMS spectrum of **2**

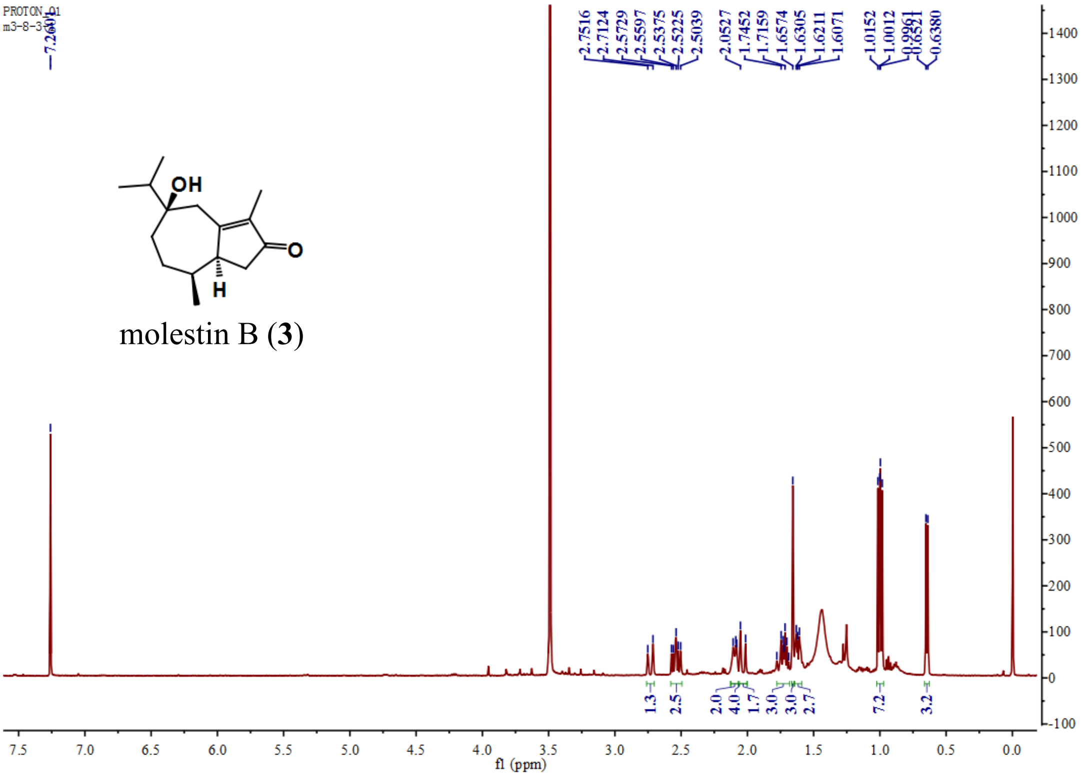

Figure S18. ^{1}H NMR (500 MHz, $CDCl_3$) spectrum of **3**

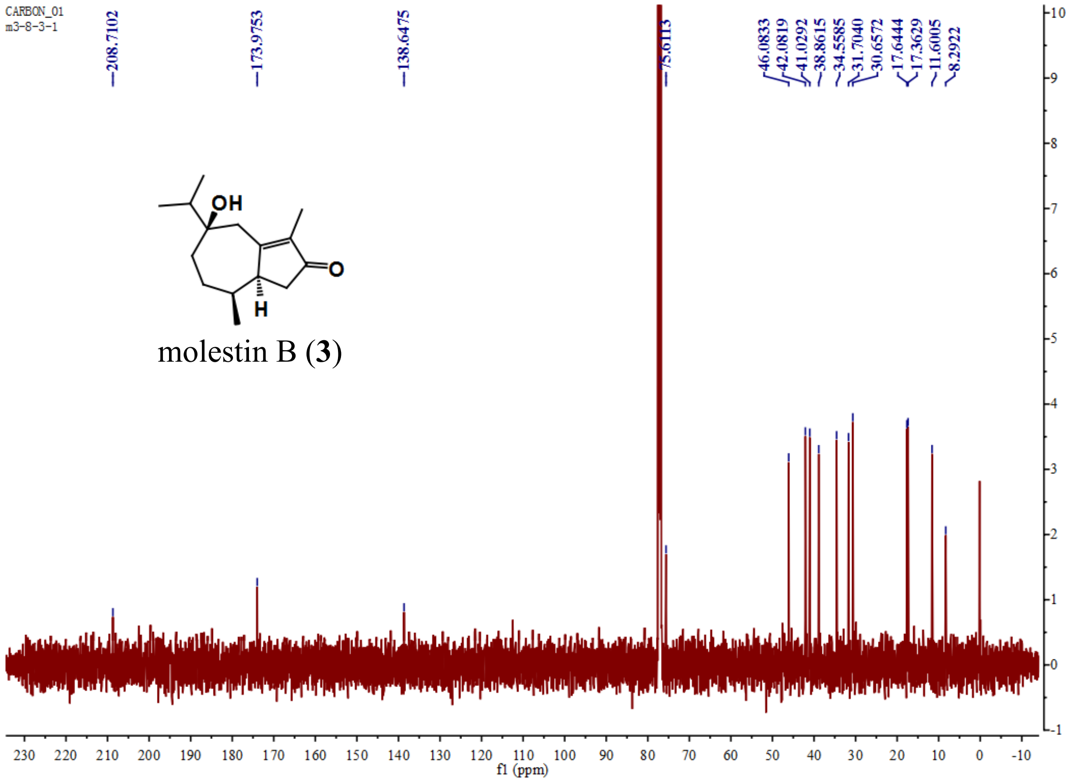

Figure S19. ^{13}C NMR (125 MHz, $CDCl_3$) spectrum of **3**

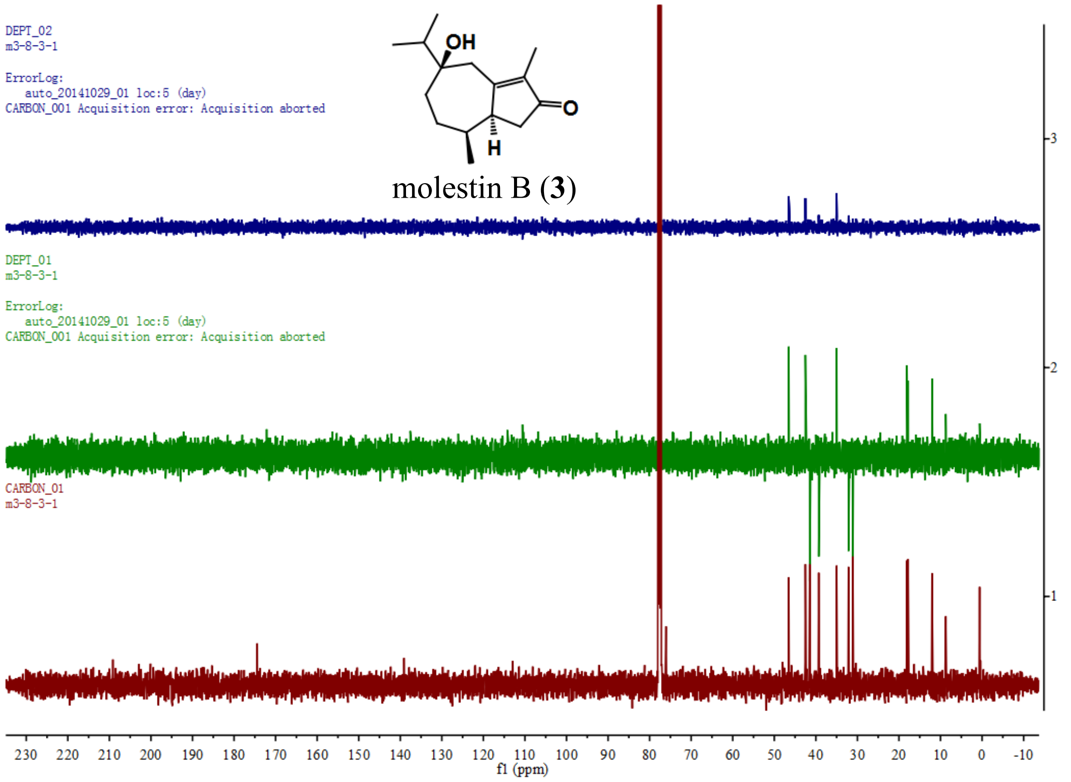

Figure S20. DEPT (125 MHz, $CDCl_3$) spectrum of **3**

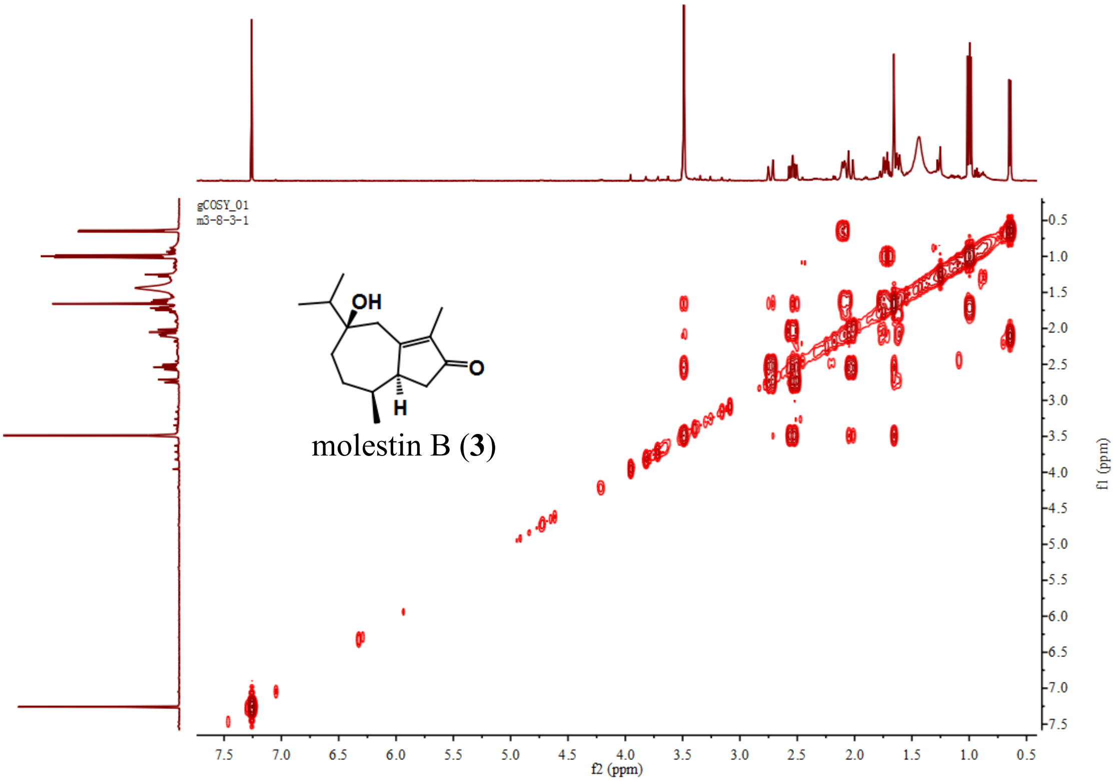

Figure S21. ^{1}H-^{1}H COSY ($CDCl_3$) spectrum of **3**

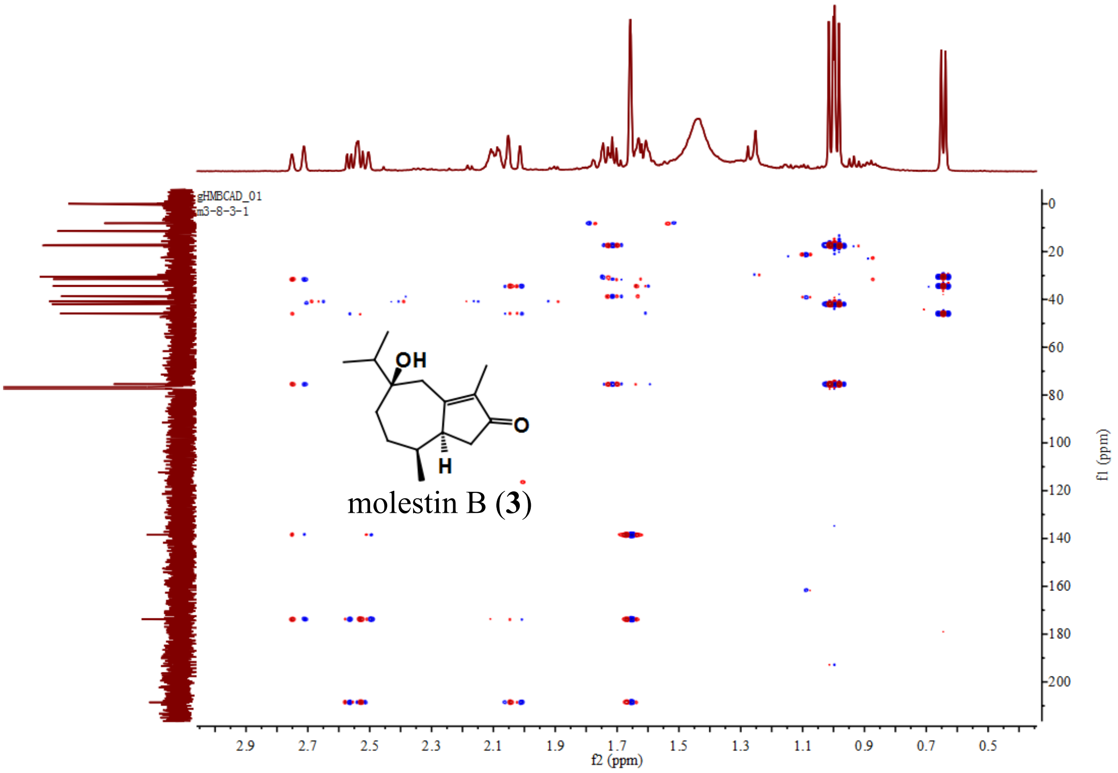

Figure S22. HMBC ($CDCl_3$) spectrum of **3**

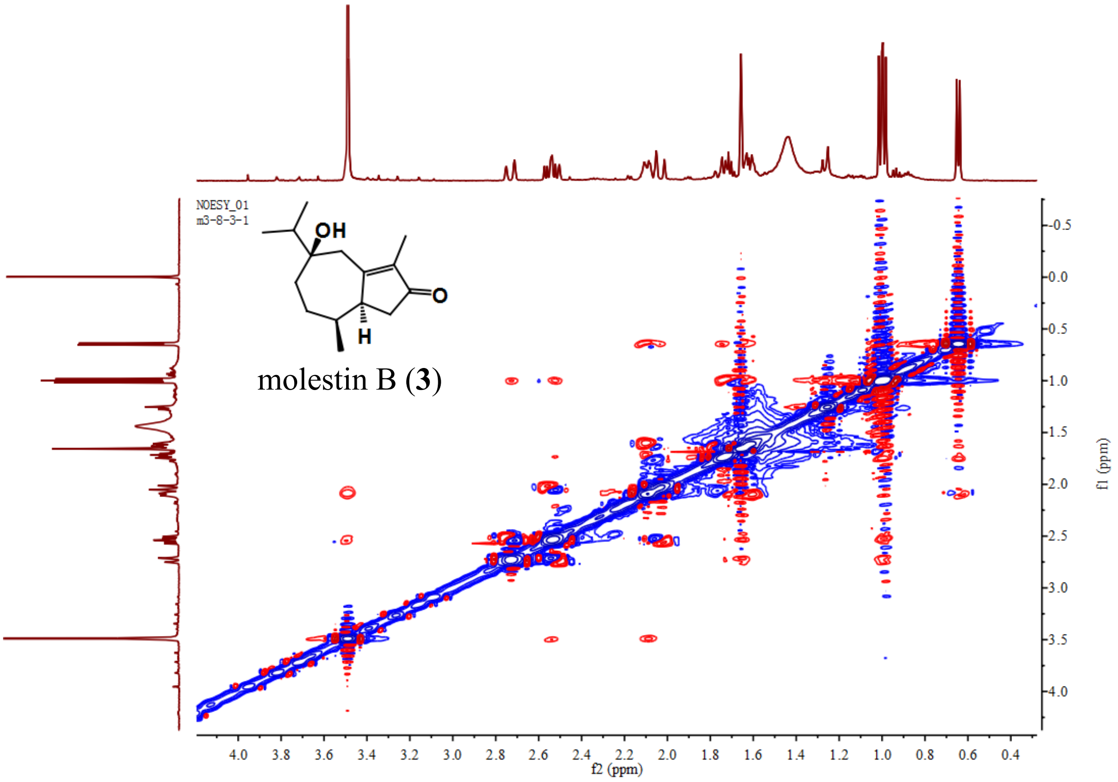

Figure S23. NOESY ($CDCl_3$) spectrum of **3**

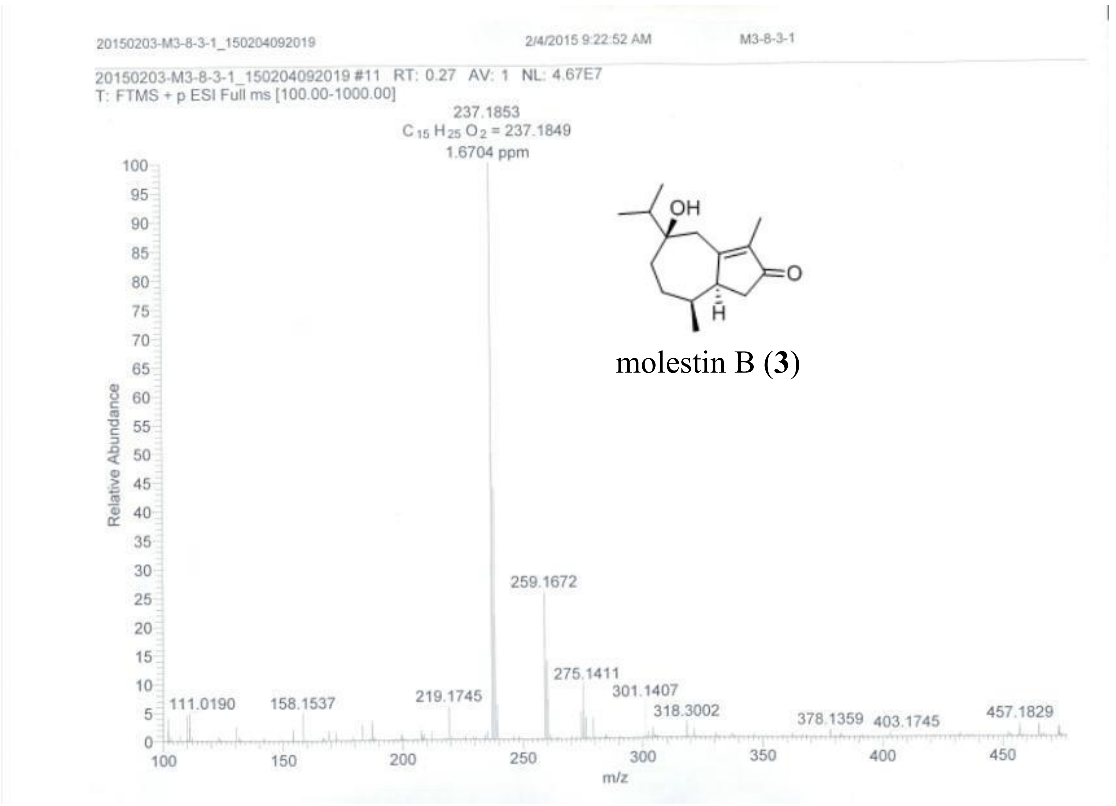

Figure S24. HRESIMS spectrum of **3**

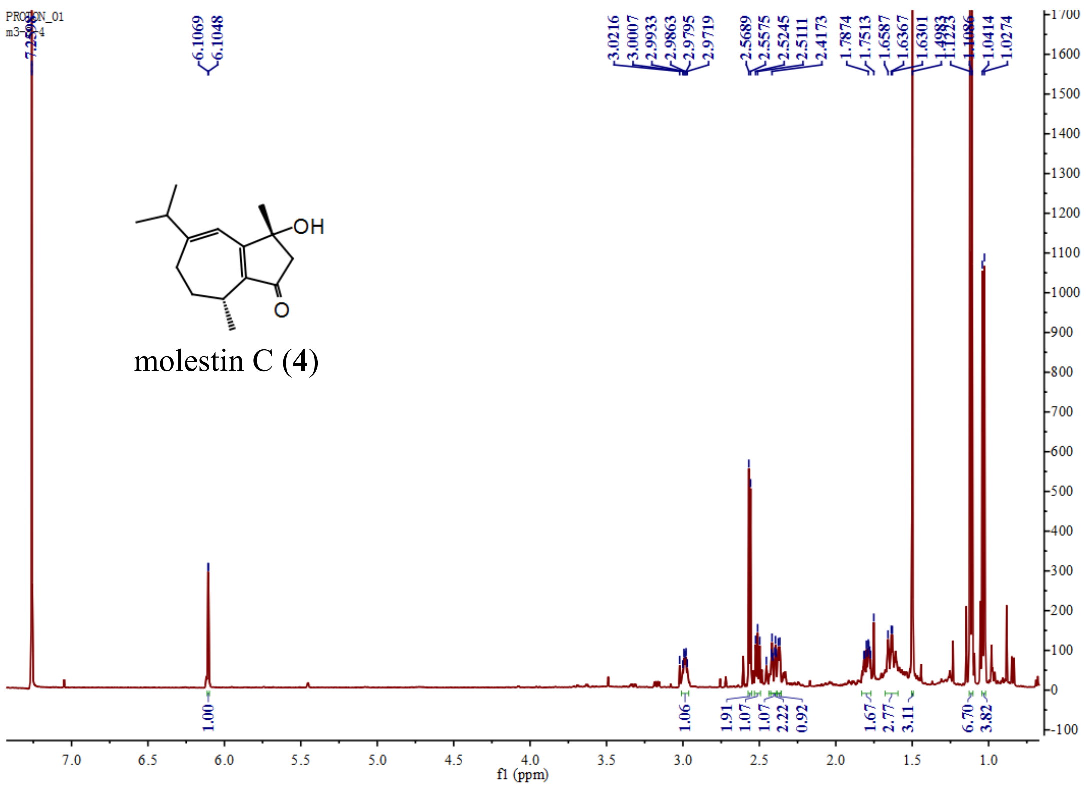

Figure S25. ^{1}H NMR (500 MHz, $CDCl_3$) spectrum of **4**

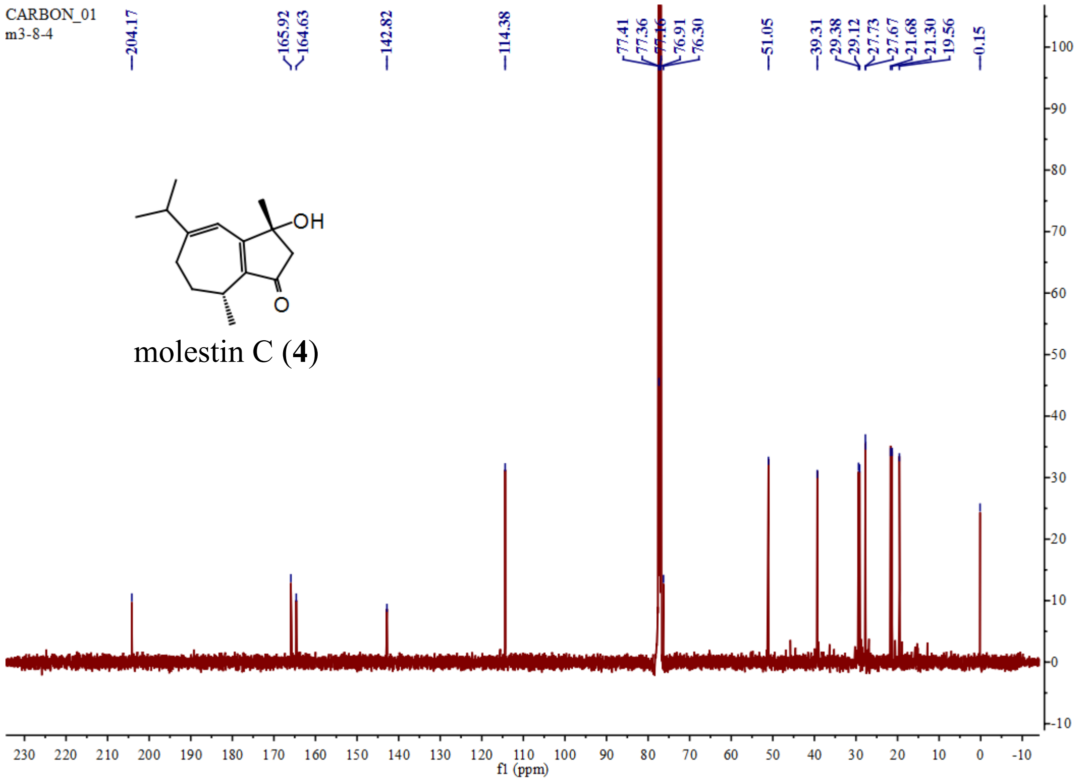

Figure S26. ^{13}C NMR (125 MHz, $CDCl_3$) spectrum of **4**

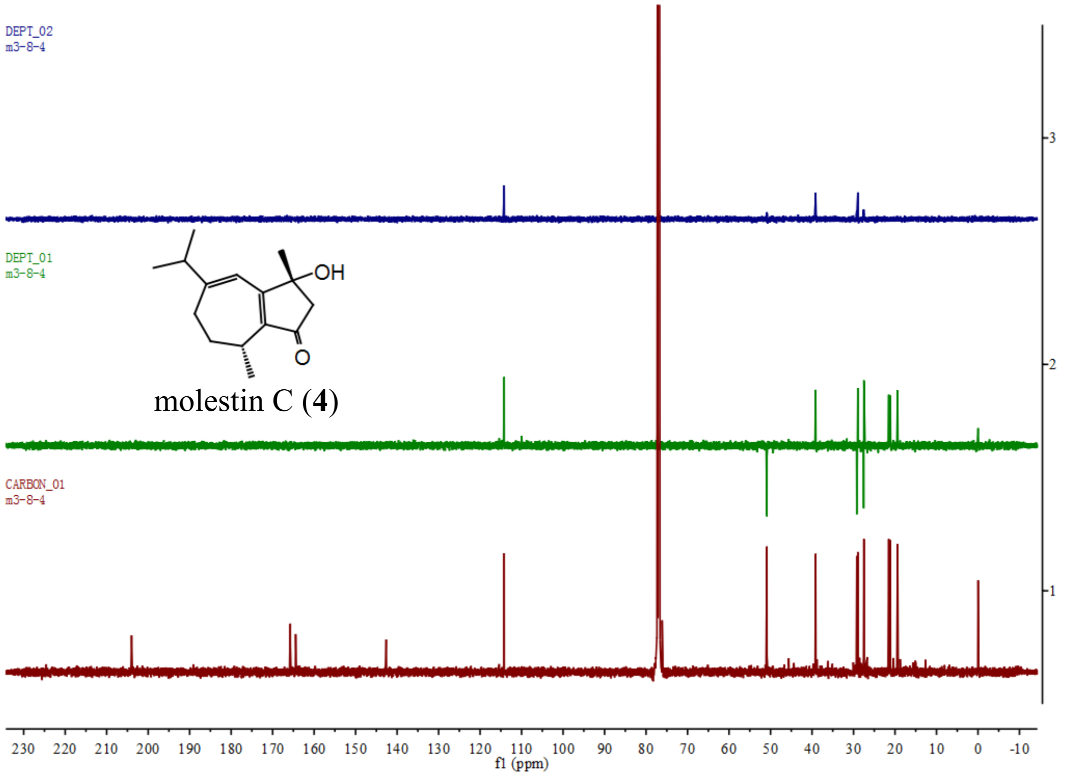

Figure S27. DEPT (125 MHz, $CDCl_3$) spectrum of **4**

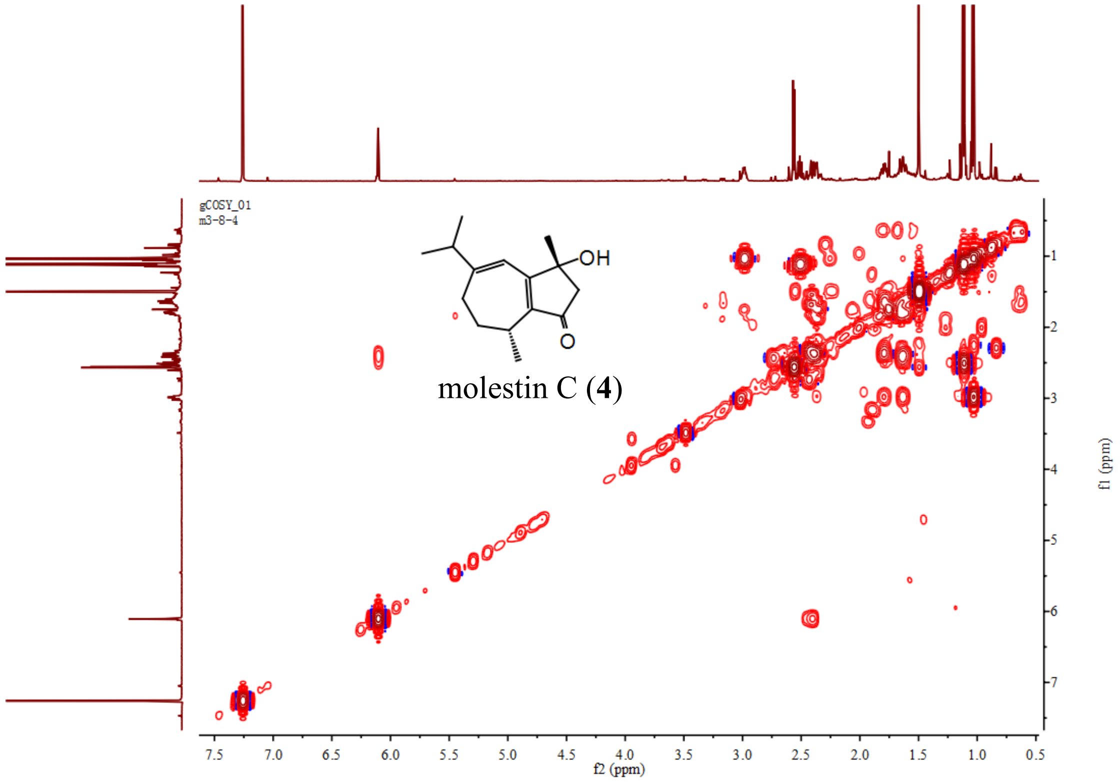

Figure S28. ^{1}H-^{1}H COSY ($CDCl_3$) spectrum of **4**

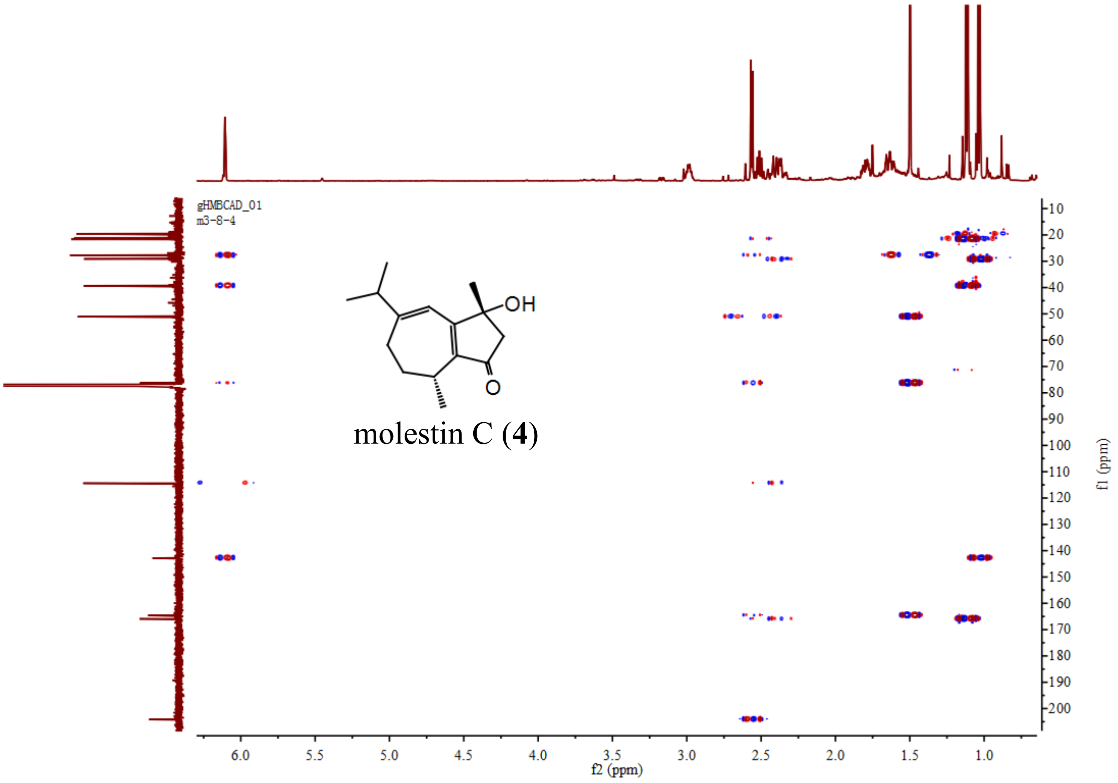

Figure S29. HMBC ($CDCl_3$) spectrum of **4**

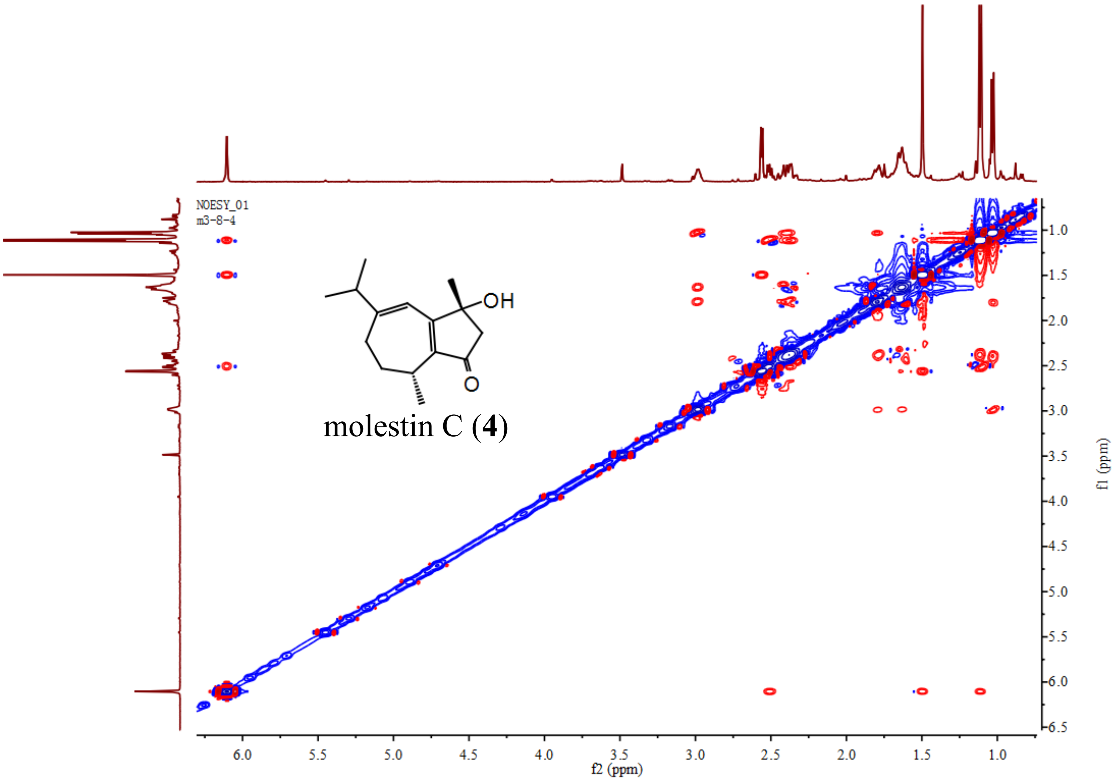

Figure S30. NOESY ($CDCl_3$) spectrum of **4**

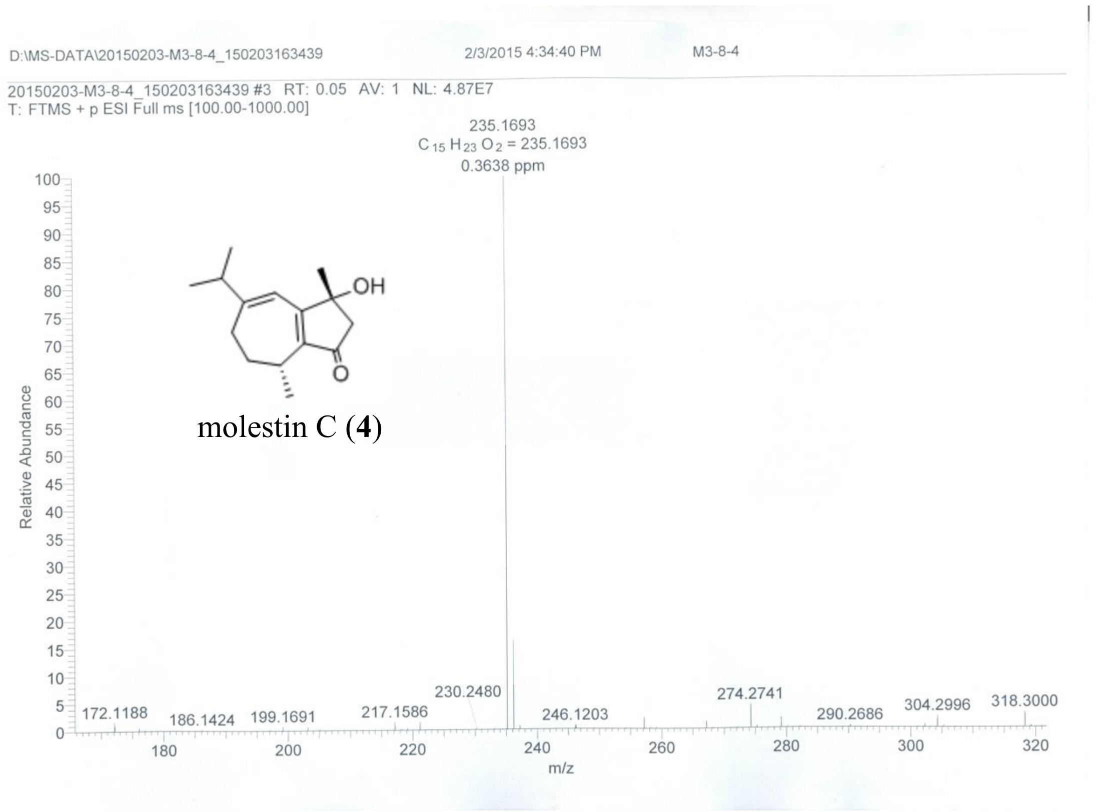

Figure S31. HRESIMS spectrum of **4**

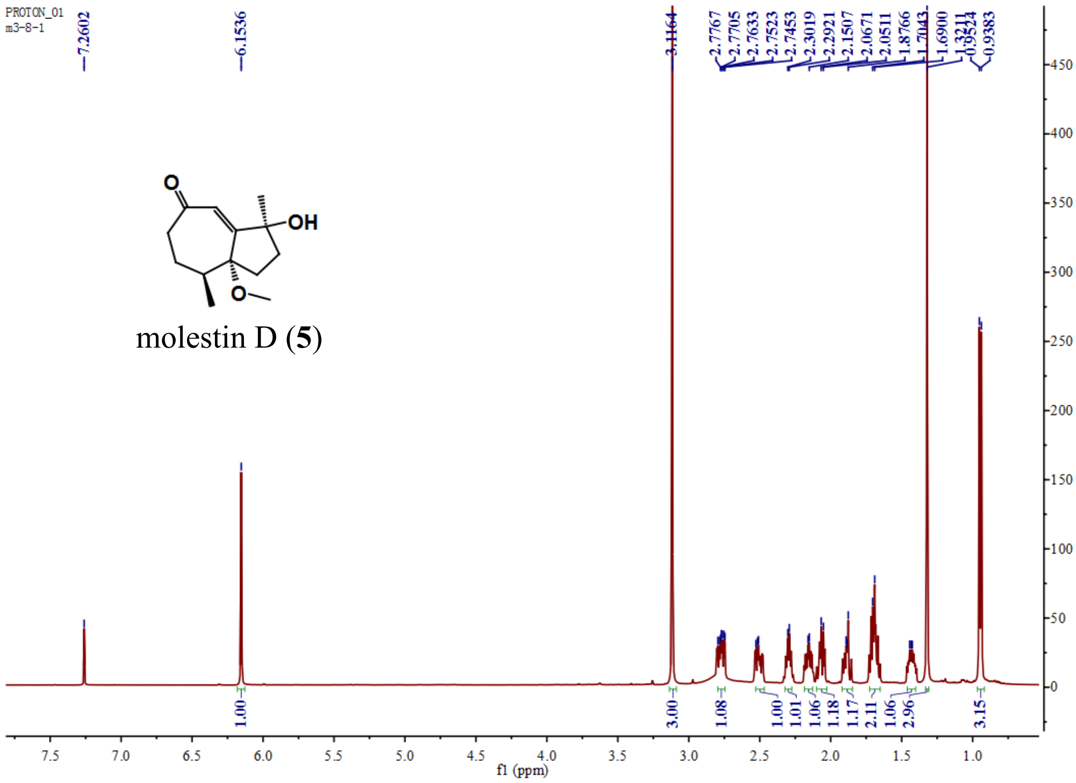

Figure S32. ^{1}H NMR ($CDCl_3$, 500 MHz) spectrum of **5**

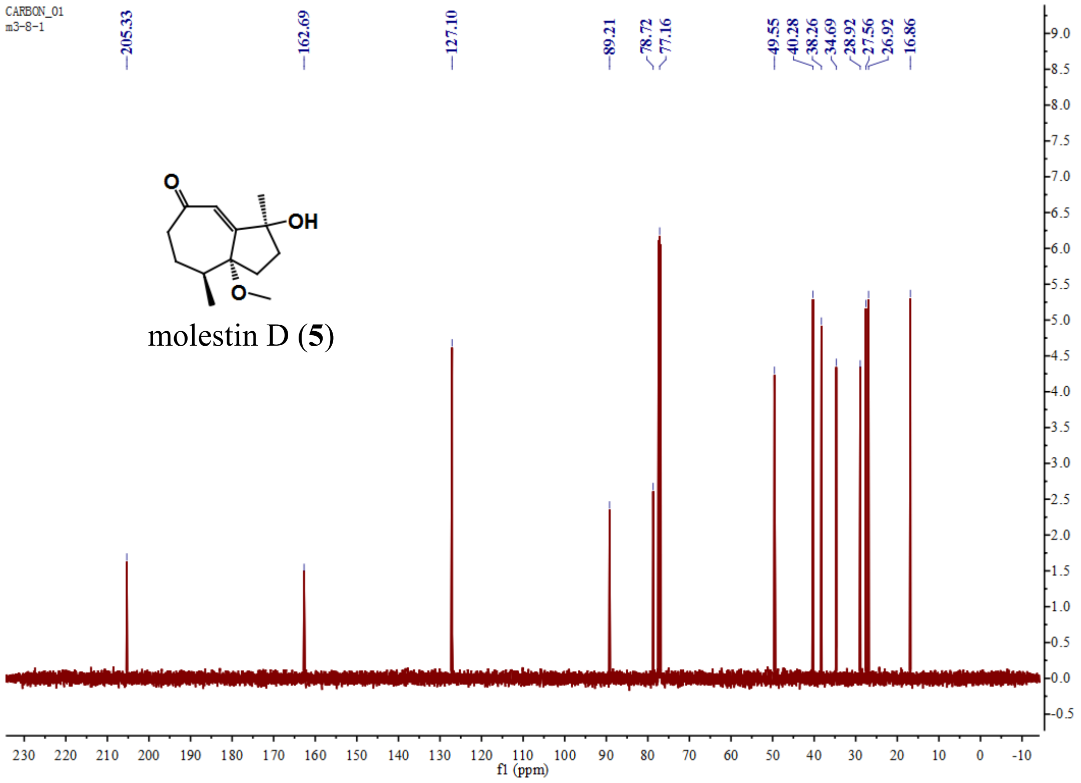

Figure S33. ^{13}C NMR ($CDCl_3$, 125 MHz) spectrum of **5**

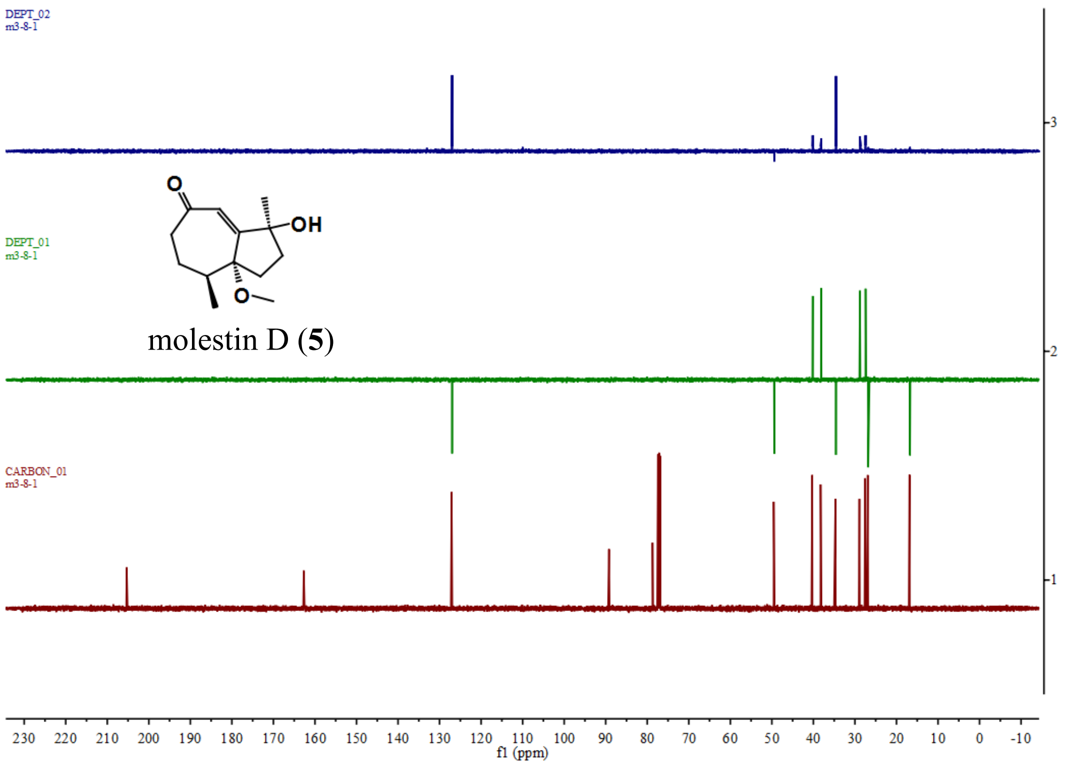

Figure S34. DEPT ($CDCl_3$, 125 MHz) spectrum of **5**

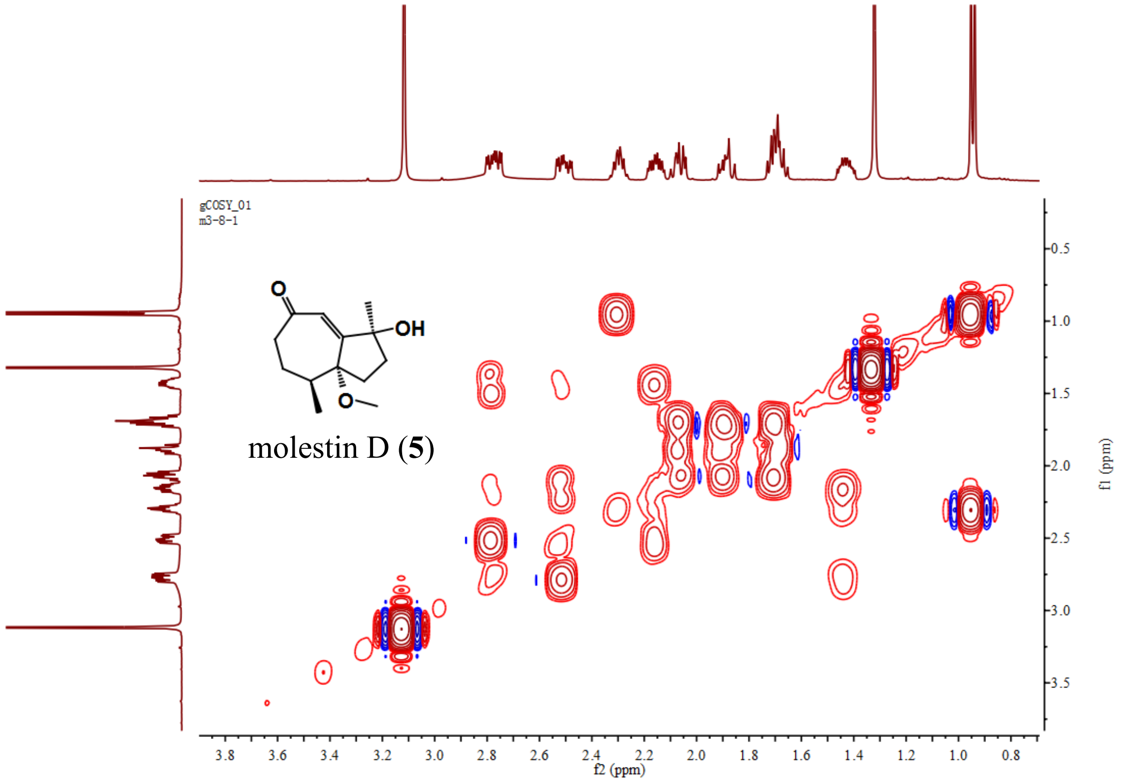

Figure S35. ^{1}H-^{1}H COSY ($CDCl_3$) spectrum of **5**

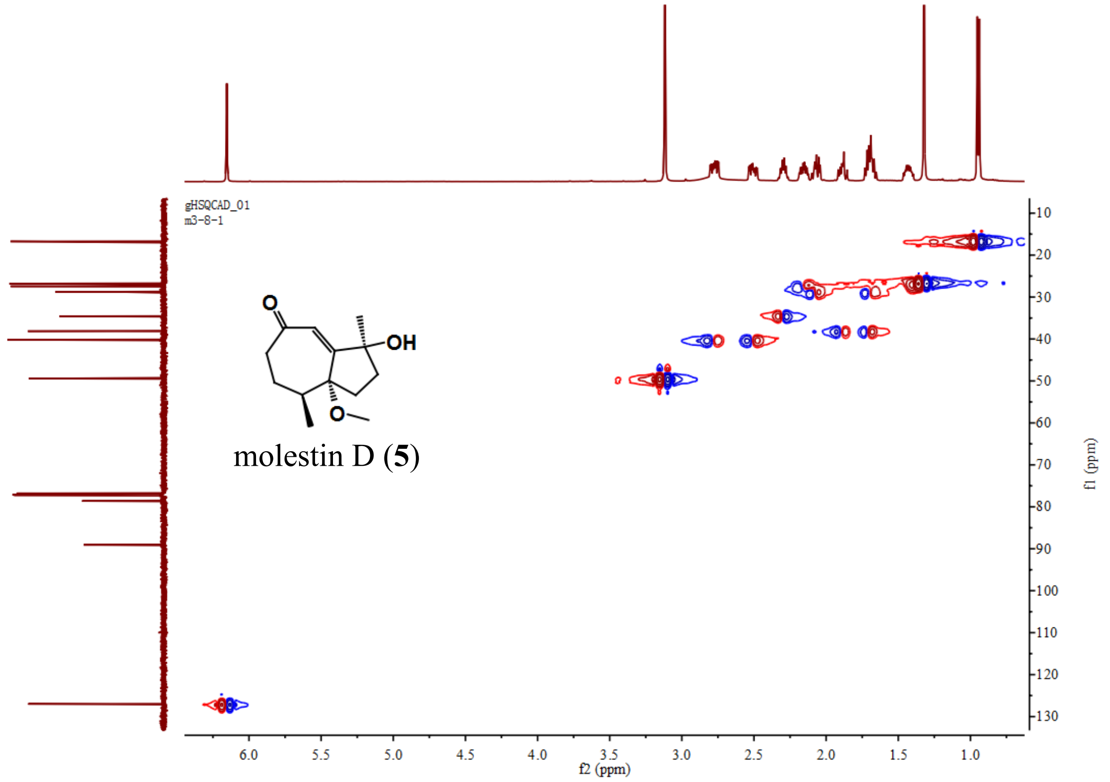

Figure S36. HSQC ($CDCl_3$) spectrum of **5**

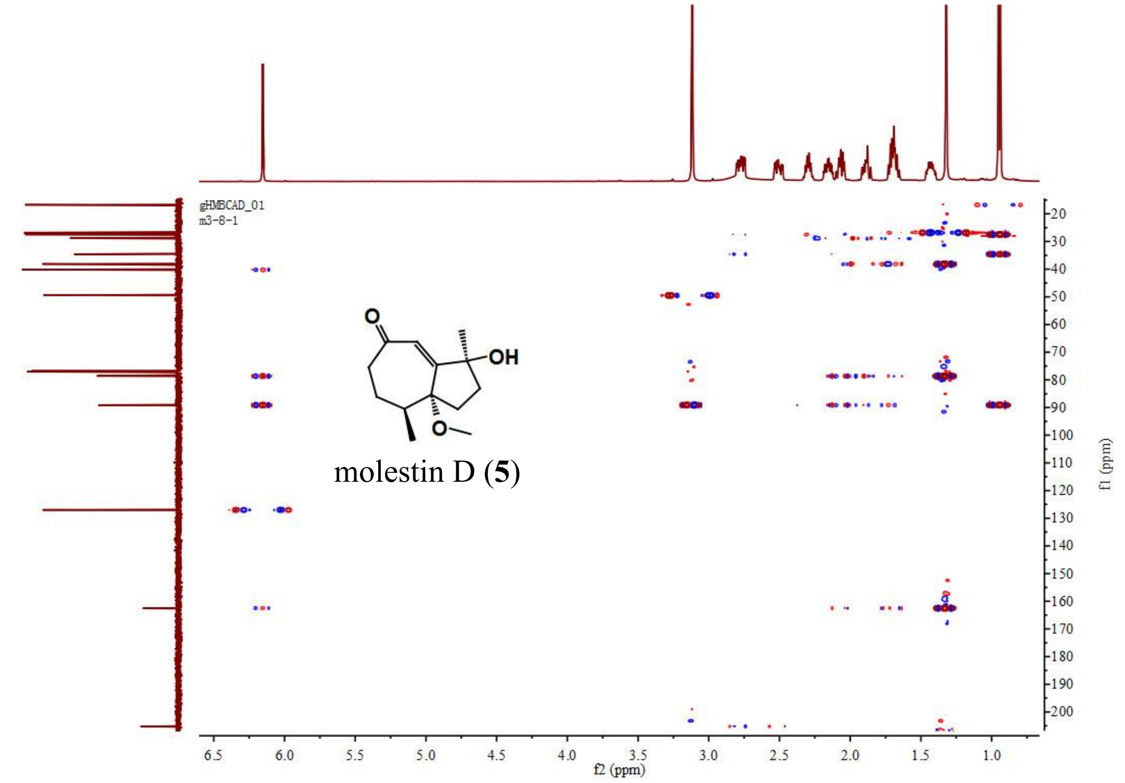

Figure S37. HMBC ($CDCl_3$) spectrum of **5**

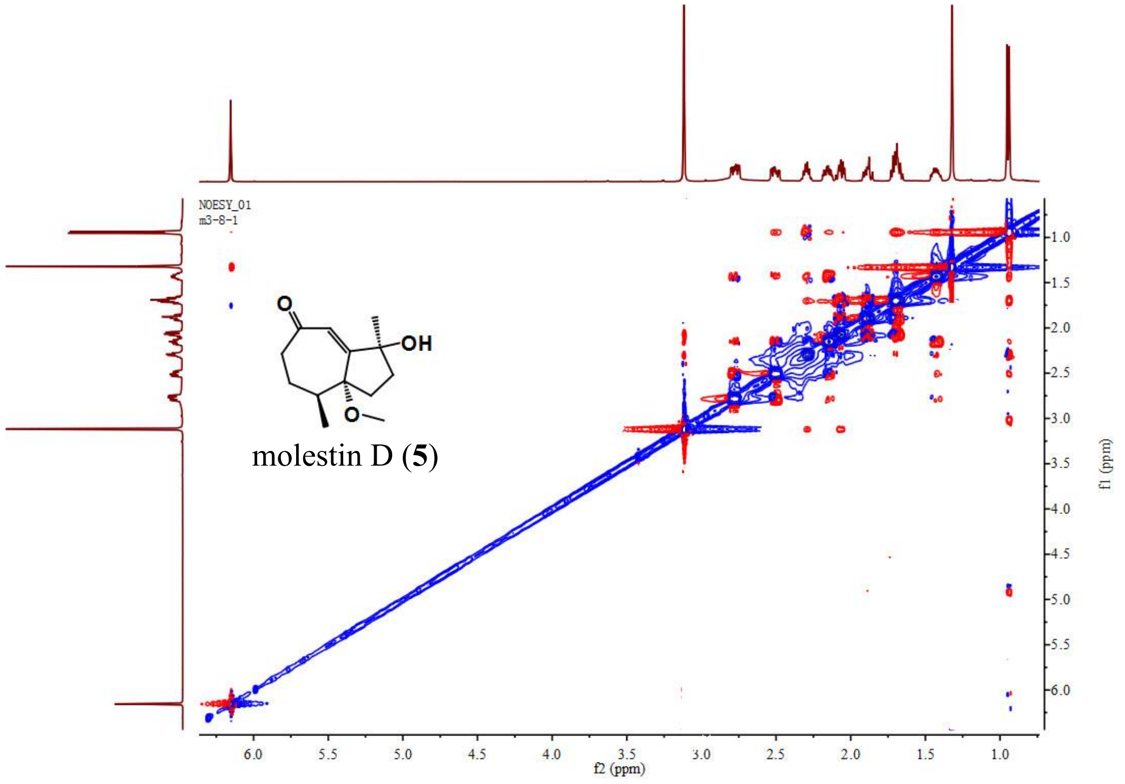

Figure S38. NOESY ($CDCl_3$) spectrum of **5**

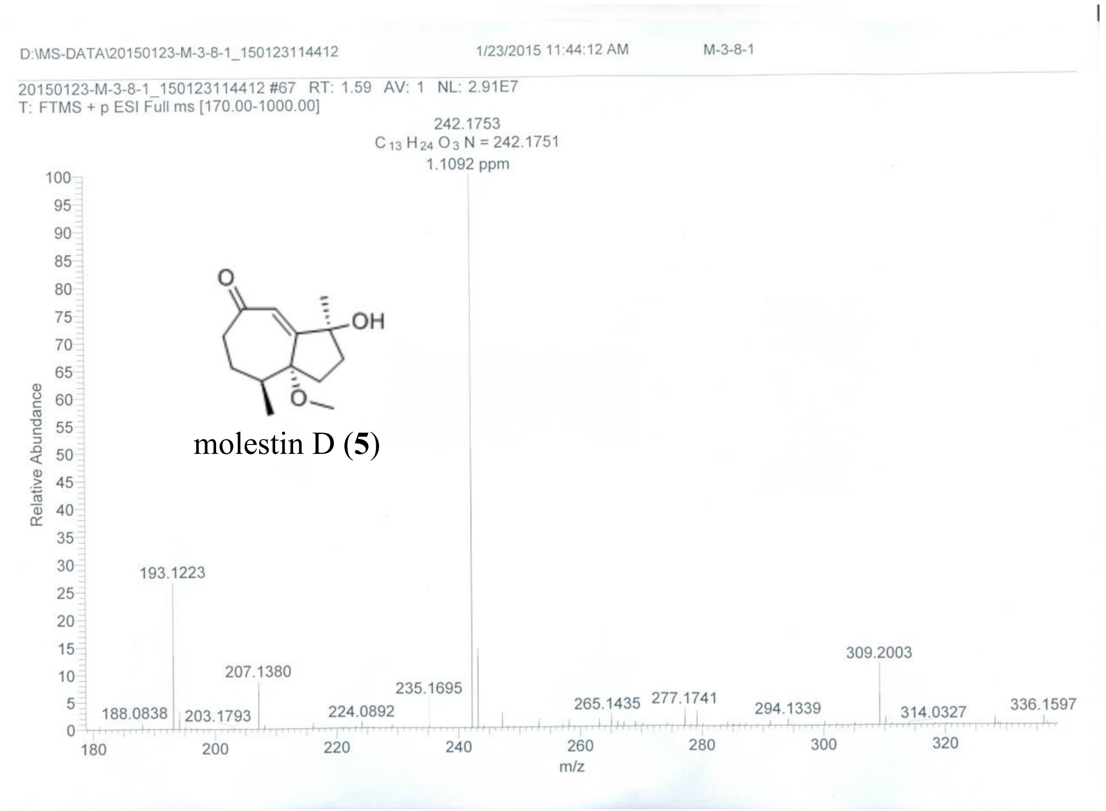

Figure S39. HRESIMS spectrum of **5**

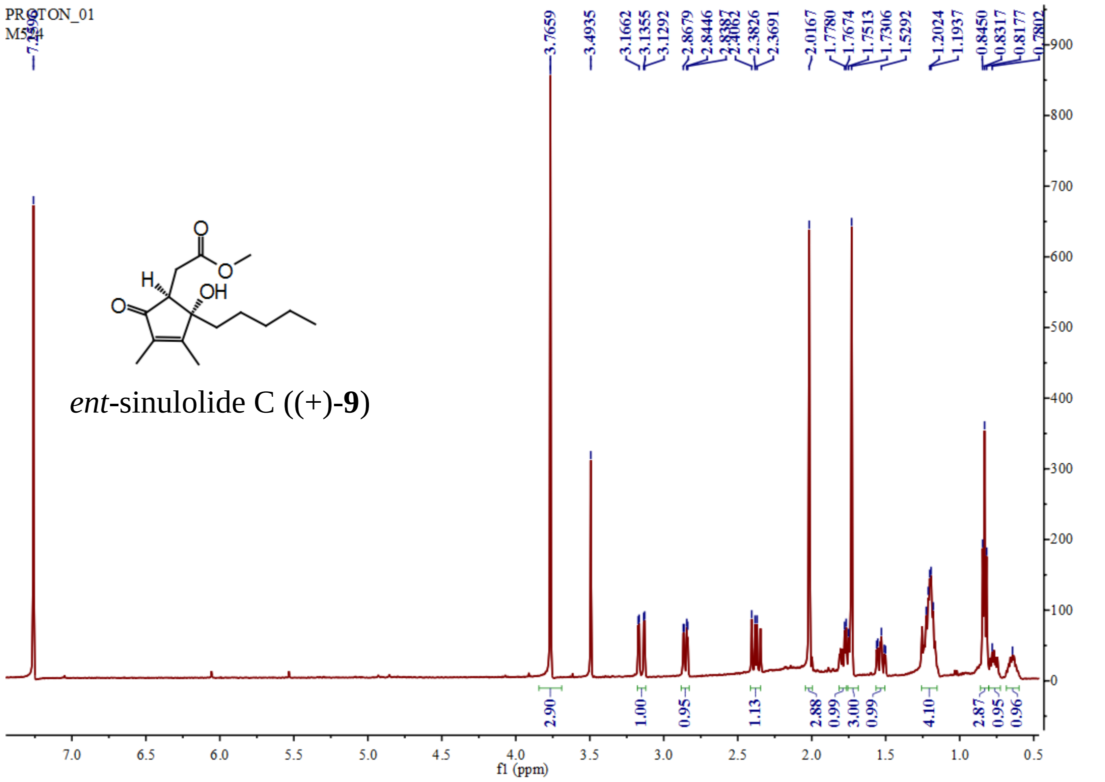

Figure S40. ^{1}H NMR ($CDCl_3$, 500 MHz) spectrum of (+)-**9**

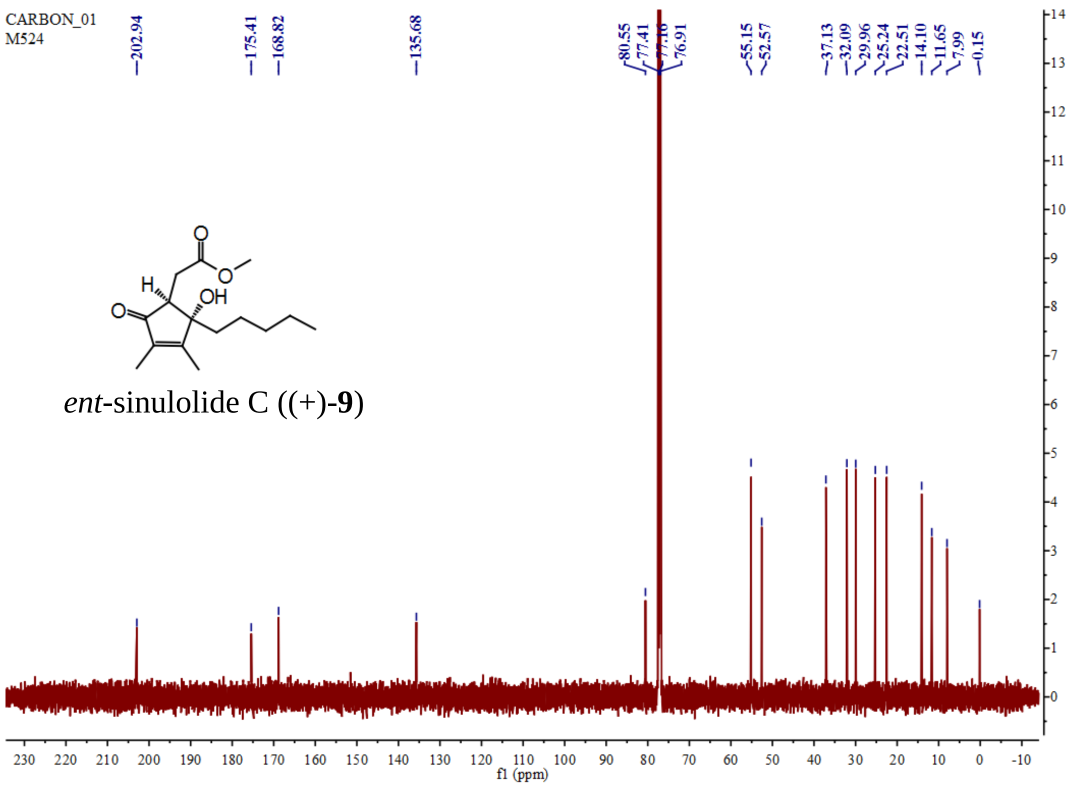

Figure S41. ^{13}C NMR ($CDCl_3$, 125 MHz) spectrum of (+)-**9**

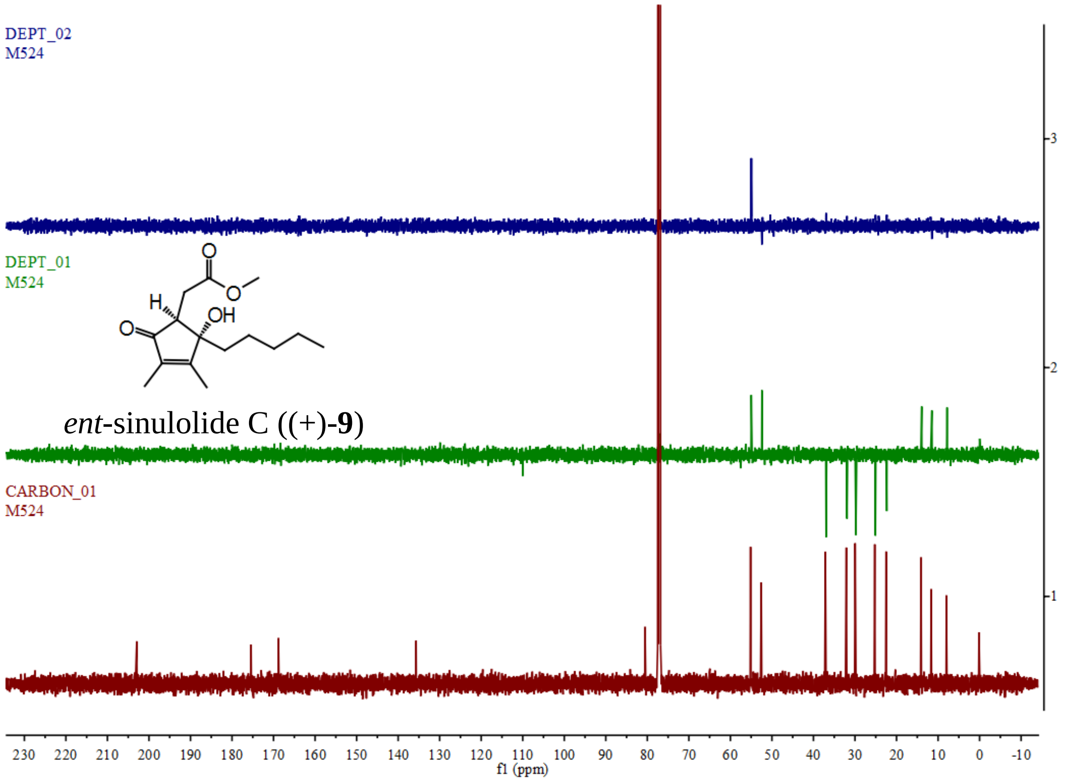

Figure S42. DEPT ($CDCl_3$, 125 MHz) spectrum of (+)-**9**

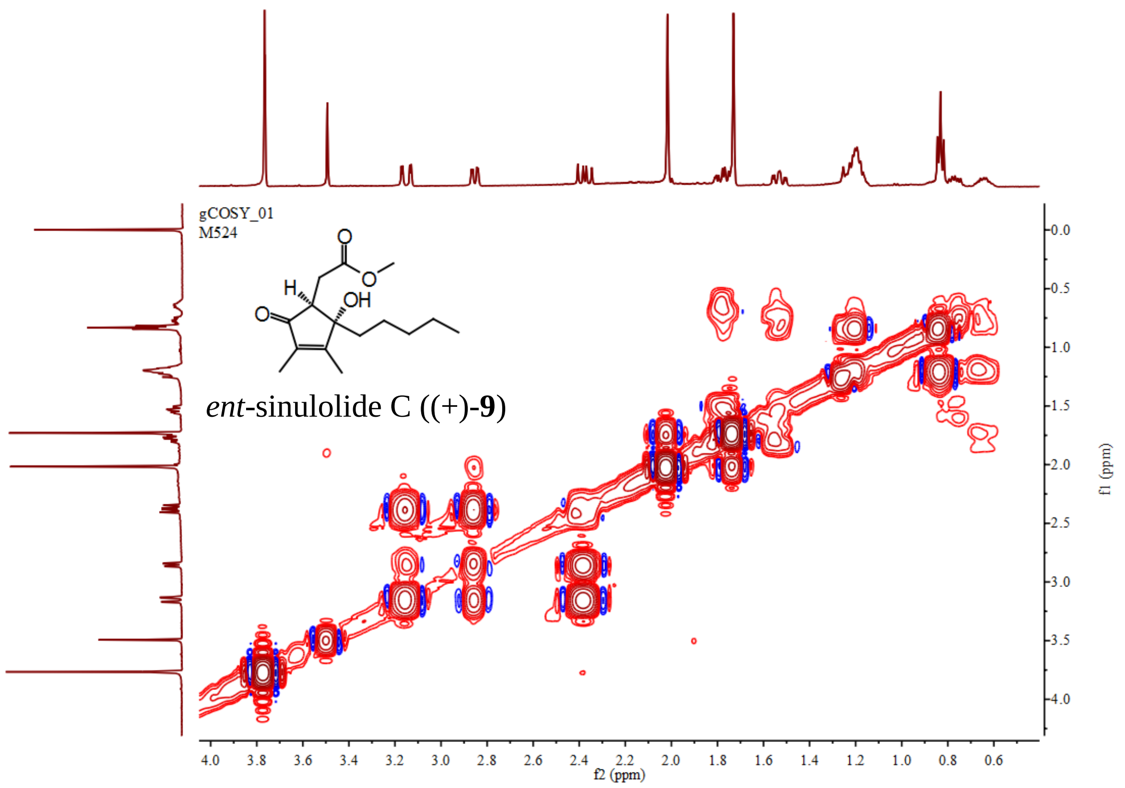

Figure S43. ^{1}H-^{1}H COSY ($CDCl_3$) spectrum of (+)-**9**

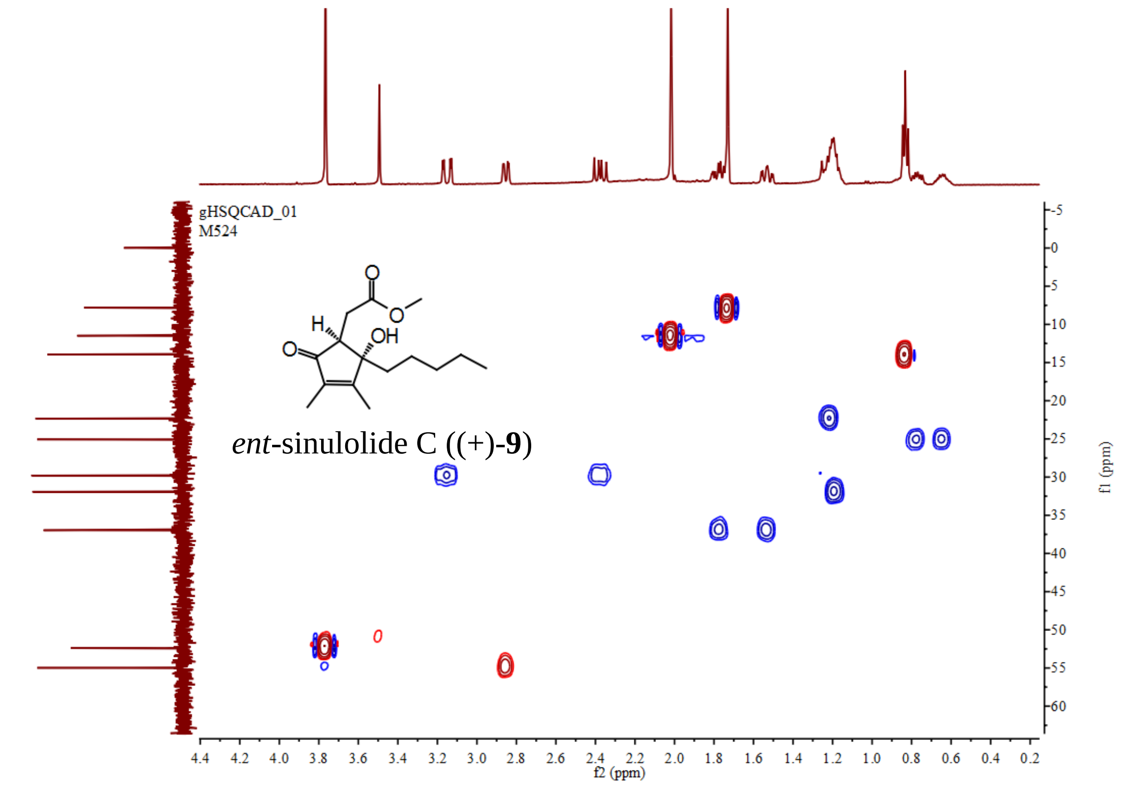

Figure S44. HSQC ($CDCl_3$) spectrum of (+)-**9**

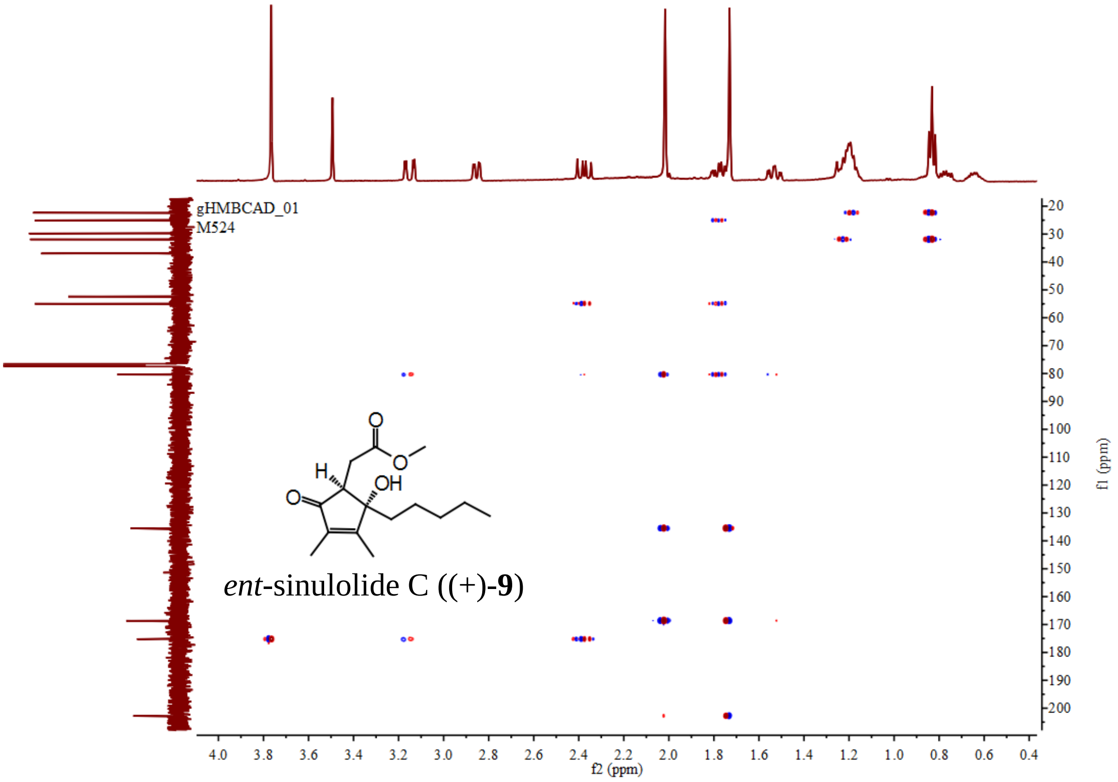

Figure S45. HMBC ($CDCl_3$) spectrum of (+)-**9**

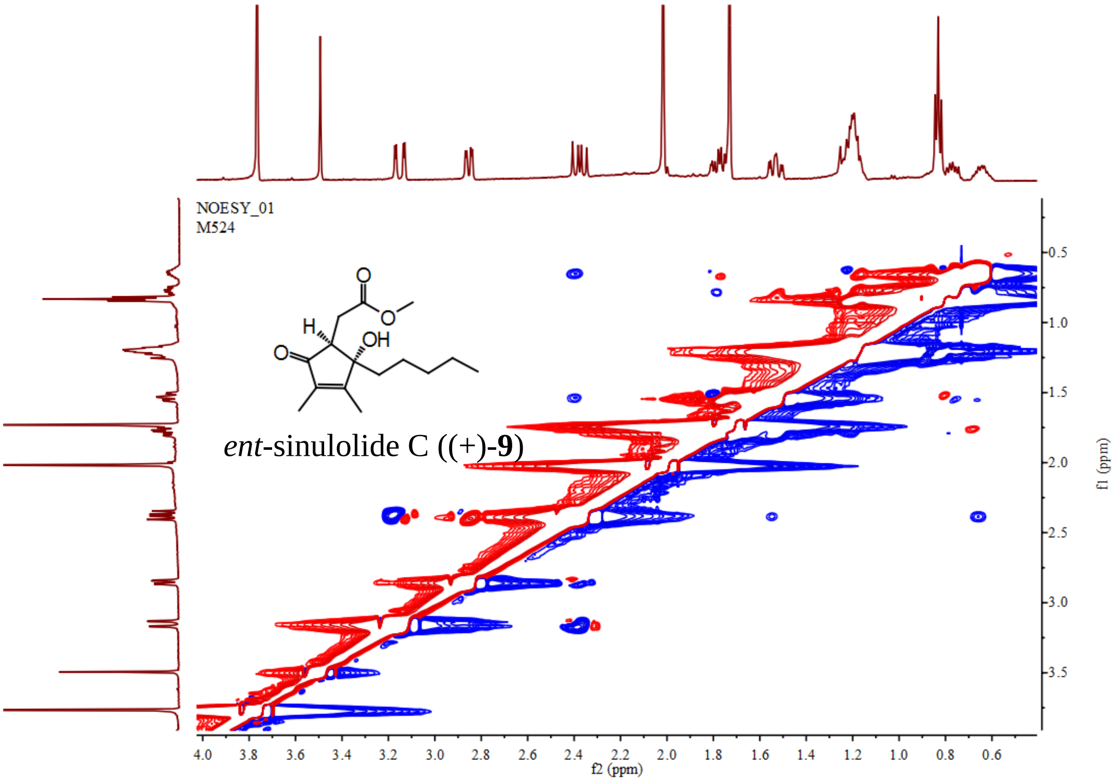

Figure S46. NOESY ($CDCl_3$) spectrum of (+)-**9**

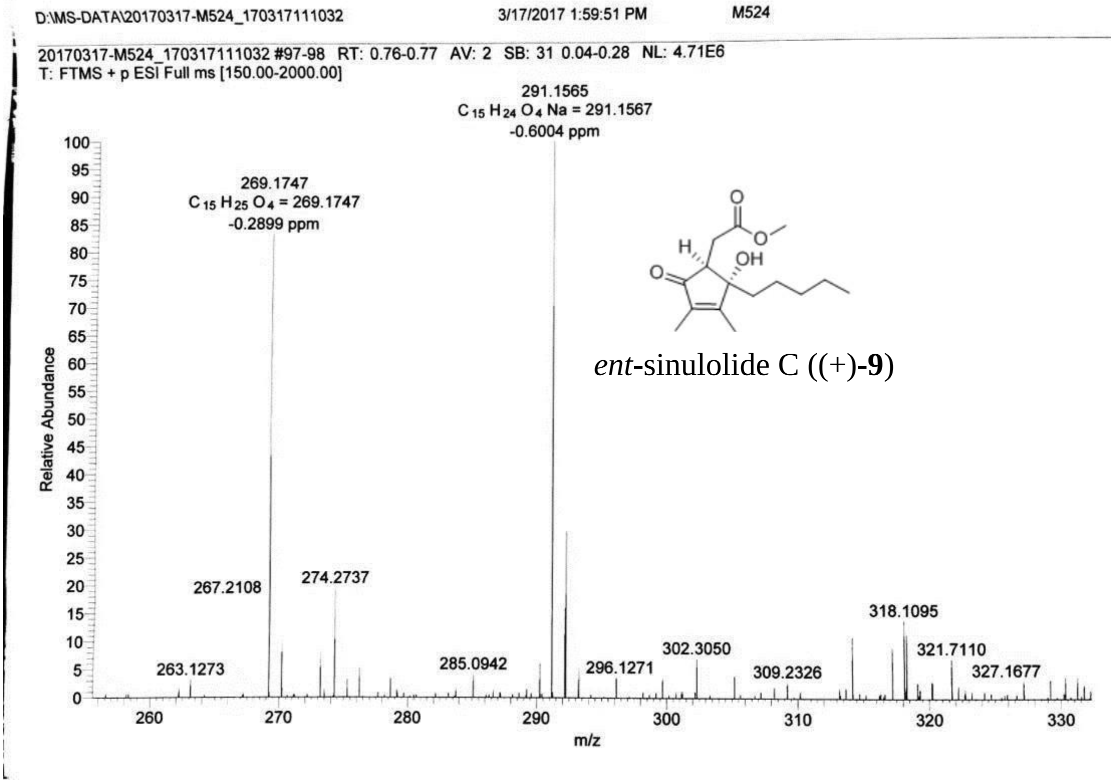

Figure S47. HRESIMS spectrum of (+)-**9**

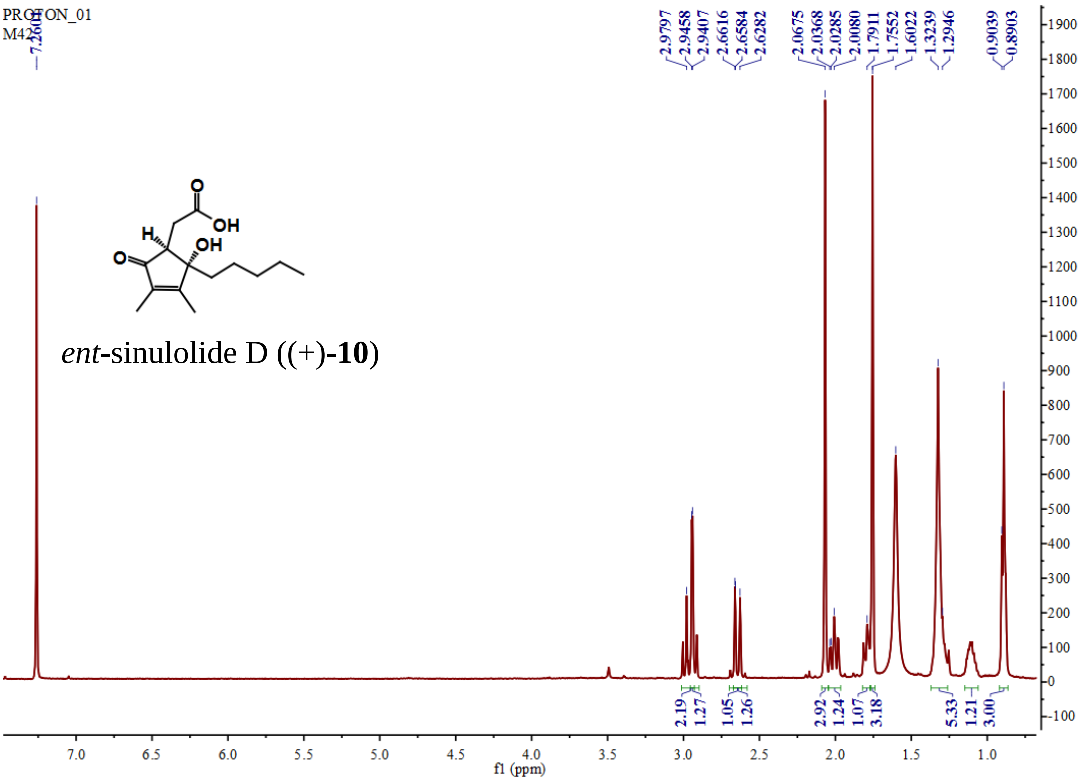

Figure S48. ^{1}H NMR ($CDCl_3$, 500 MHz) spectrum of (+)-**10**

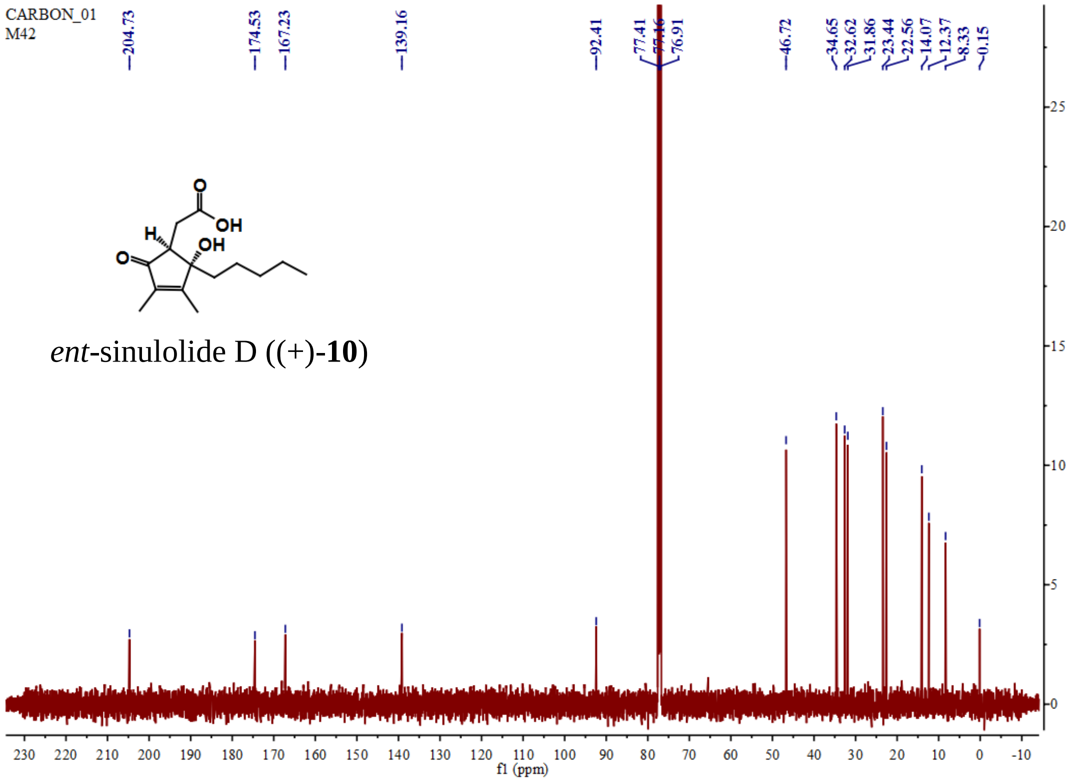

Figure S49. ^{13}C NMR ($CDCl_3$, 125 MHz) spectrum of (+)-**10**

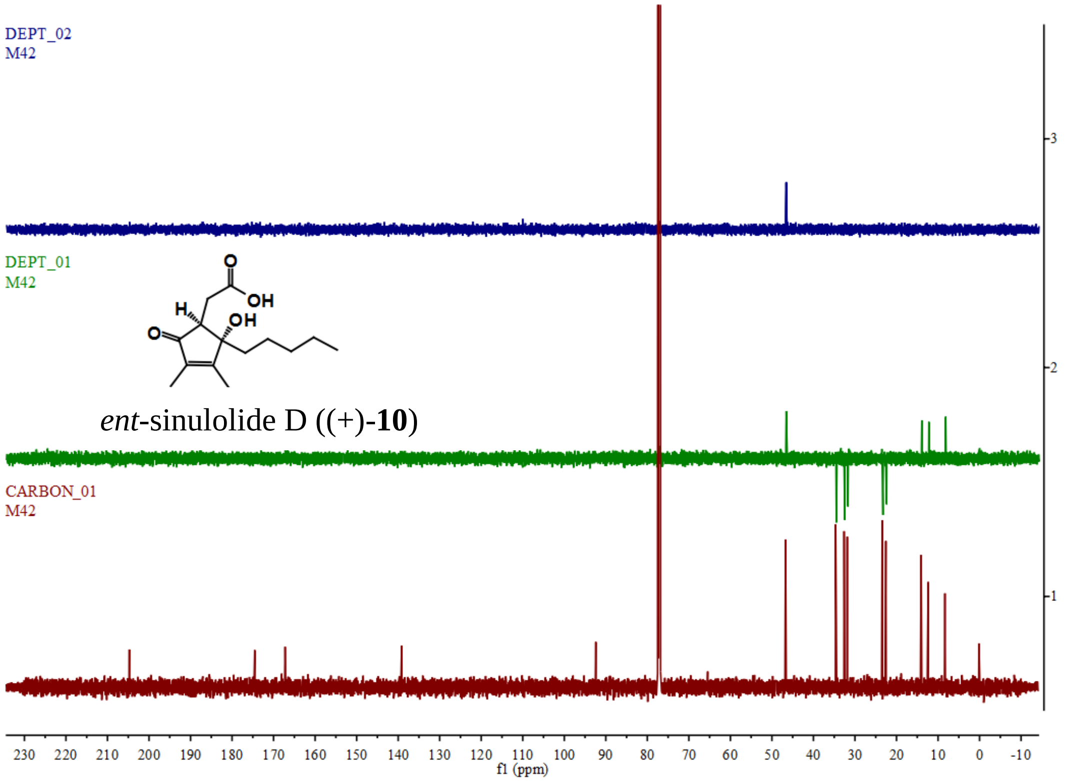

Figure S50. DEPT ($CDCl_3$, 125 MHz) spectrum of (+)-**10**

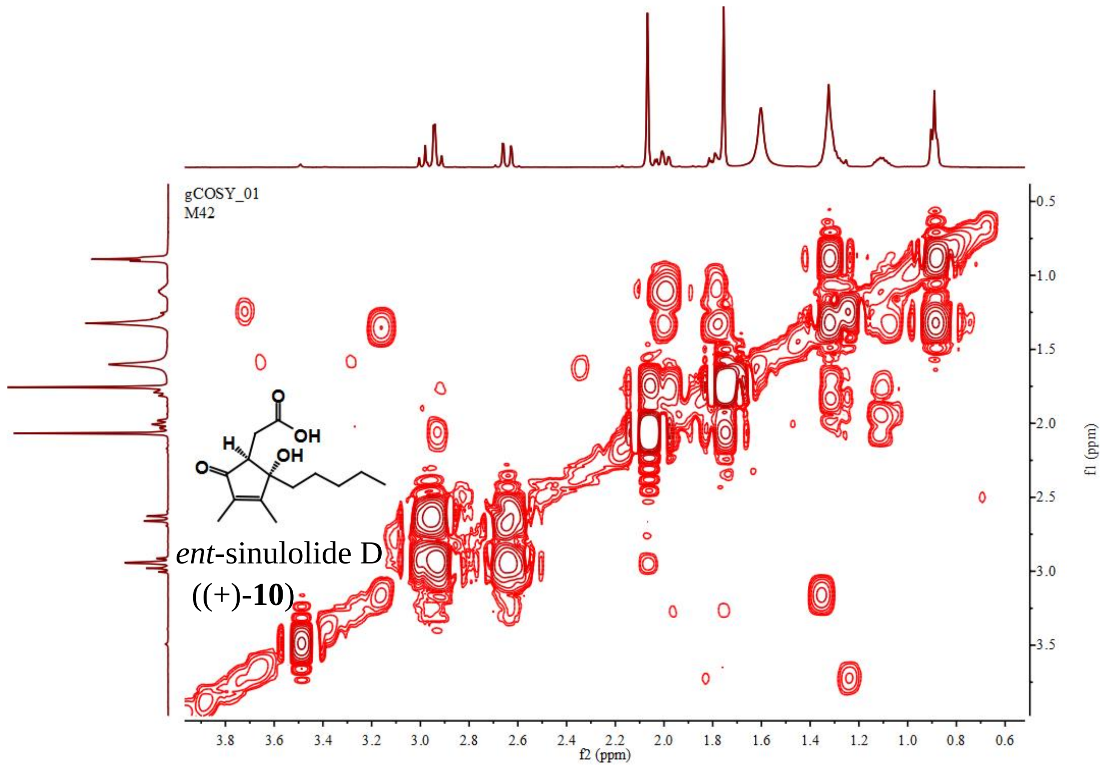

Figure S51. ^1H-^1H COSY ($CDCl_3$) spectrum of (+)-**10**

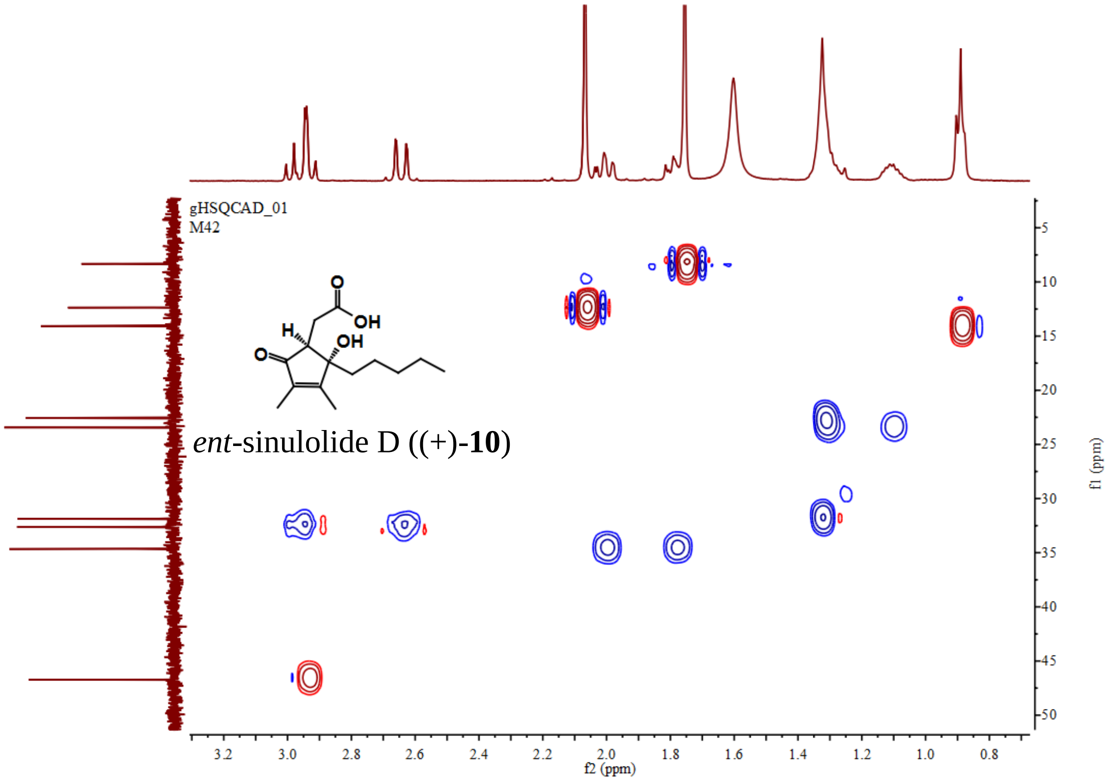

Figure S52. HSQC ($CDCl_3$) spectrum of (+)-**10**

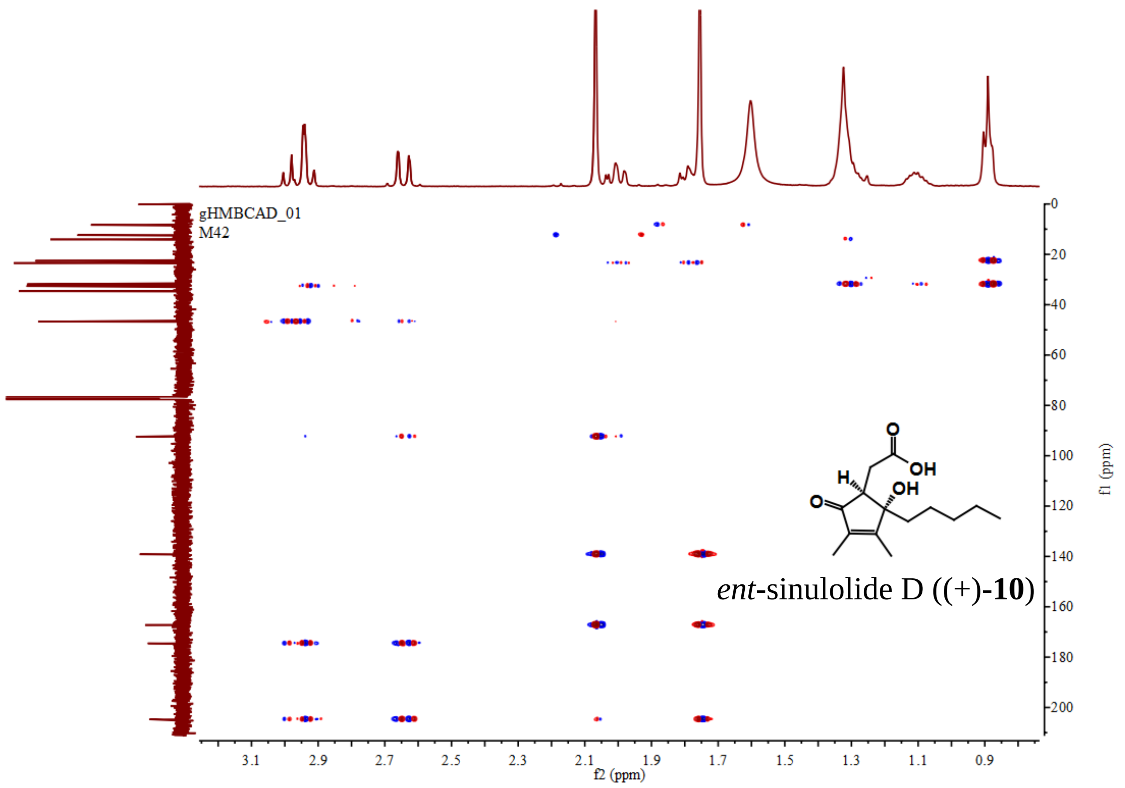

Figure S53. HMBC ($CDCl_3$) spectrum of (+)-**10**

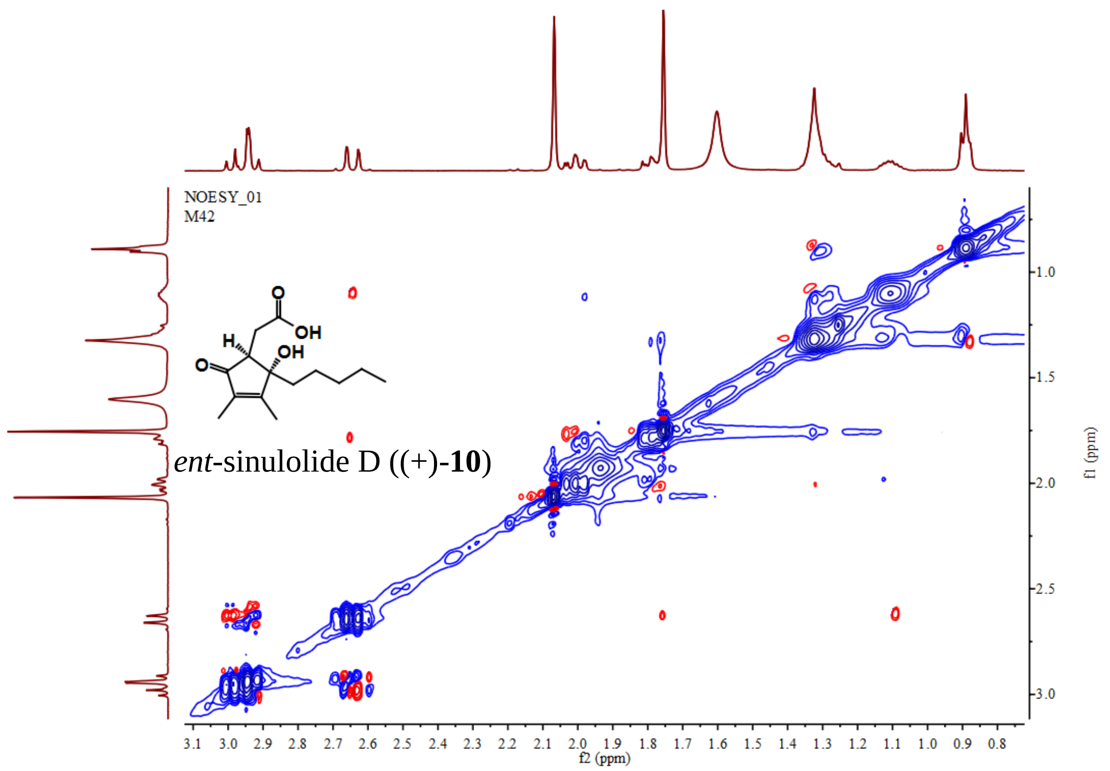

Figure S54. NOESY ($CDCl_3$) spectrum of (+)-**10**

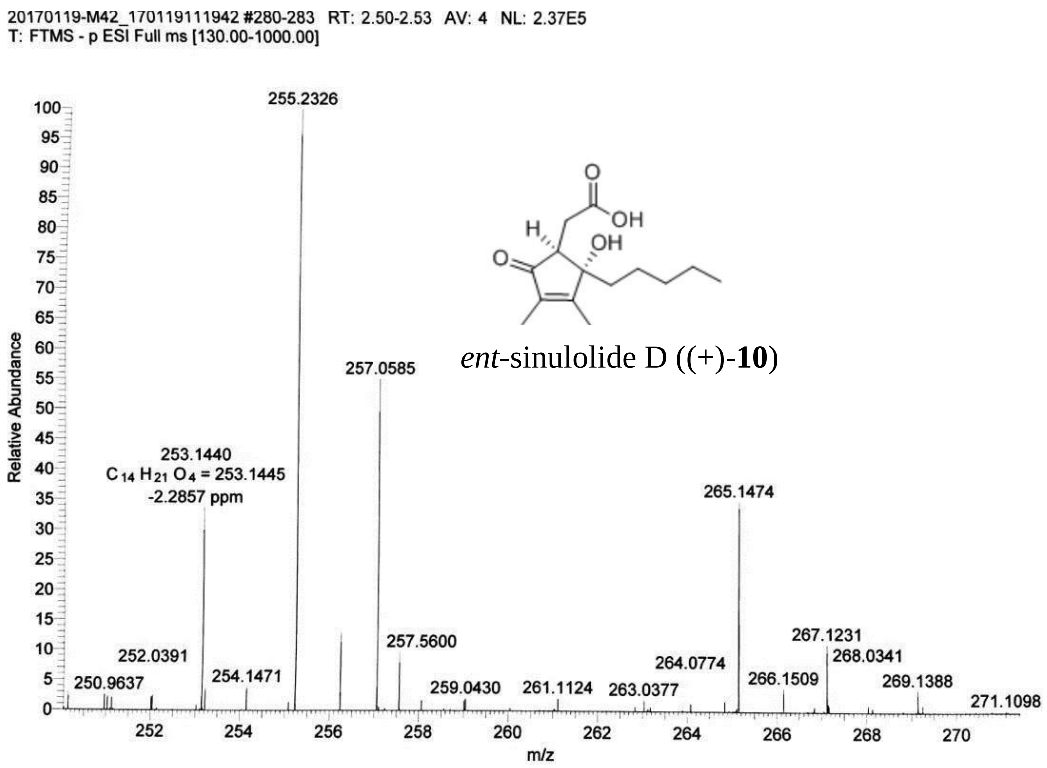

Figure S55. HRESIMS spectrum of (+)-**10**

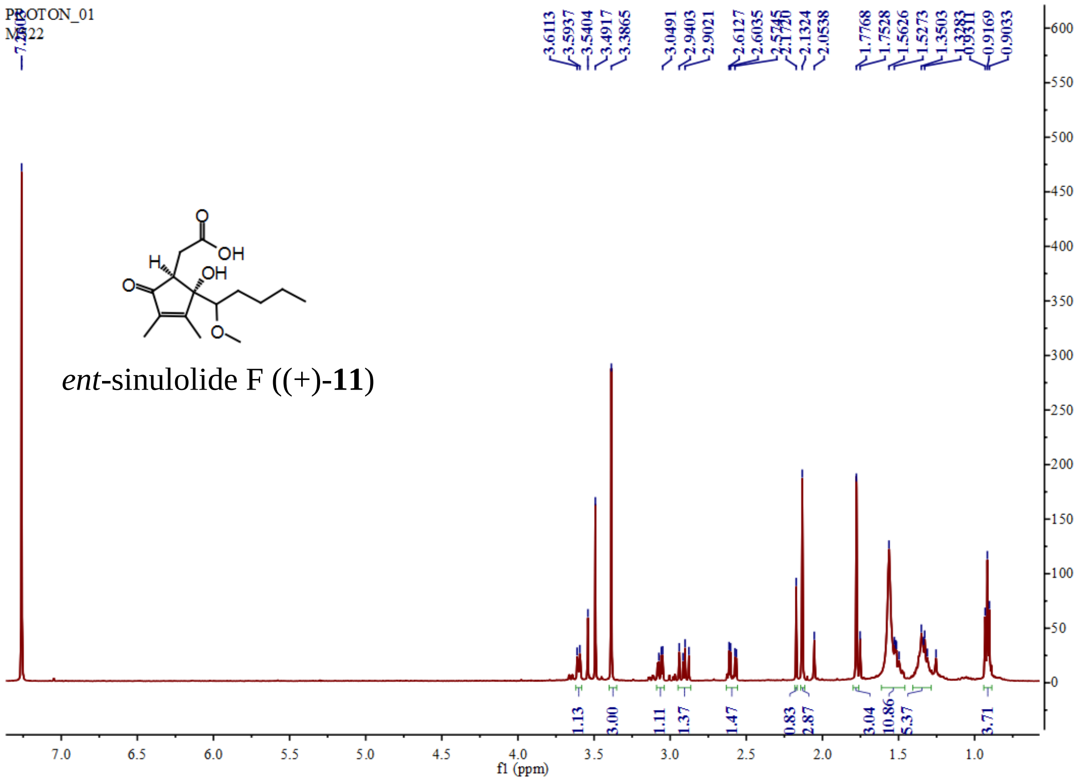

Figure S56. ^{1}H NMR ($CDCl_3$, 500 MHz) spectrum of (+)-**11**

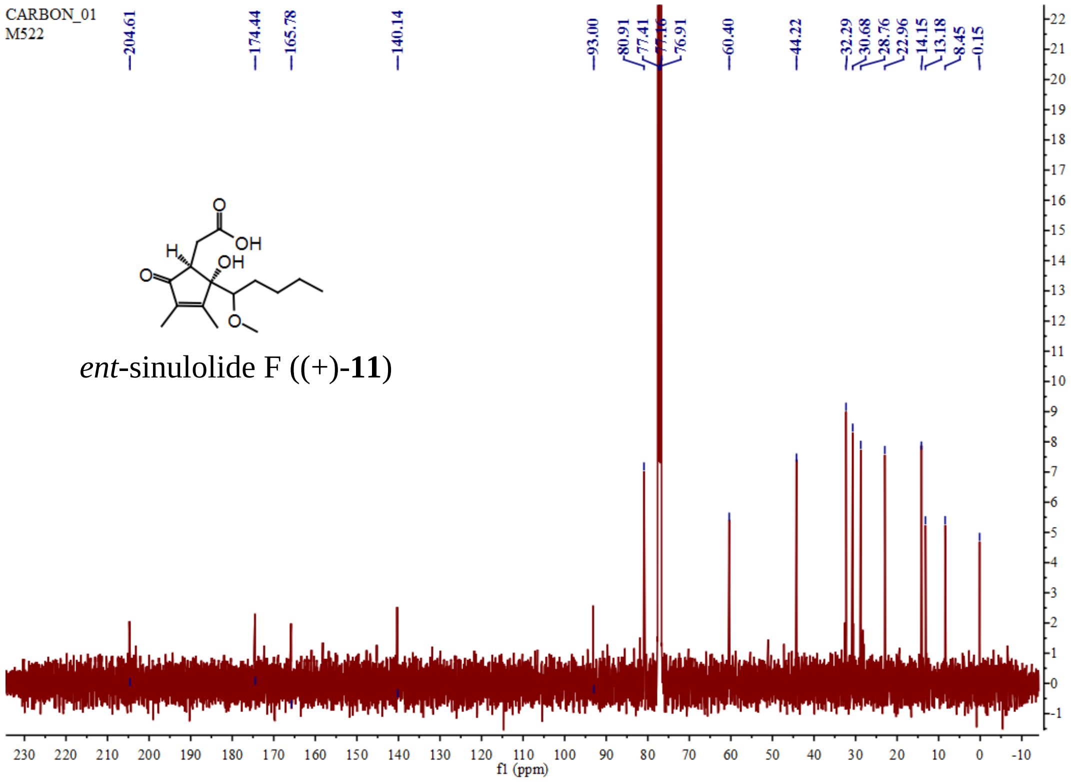

Figure S57. ^{13}C NMR ($CDCl_3$, 125 MHz) spectrum of (+)-**11**

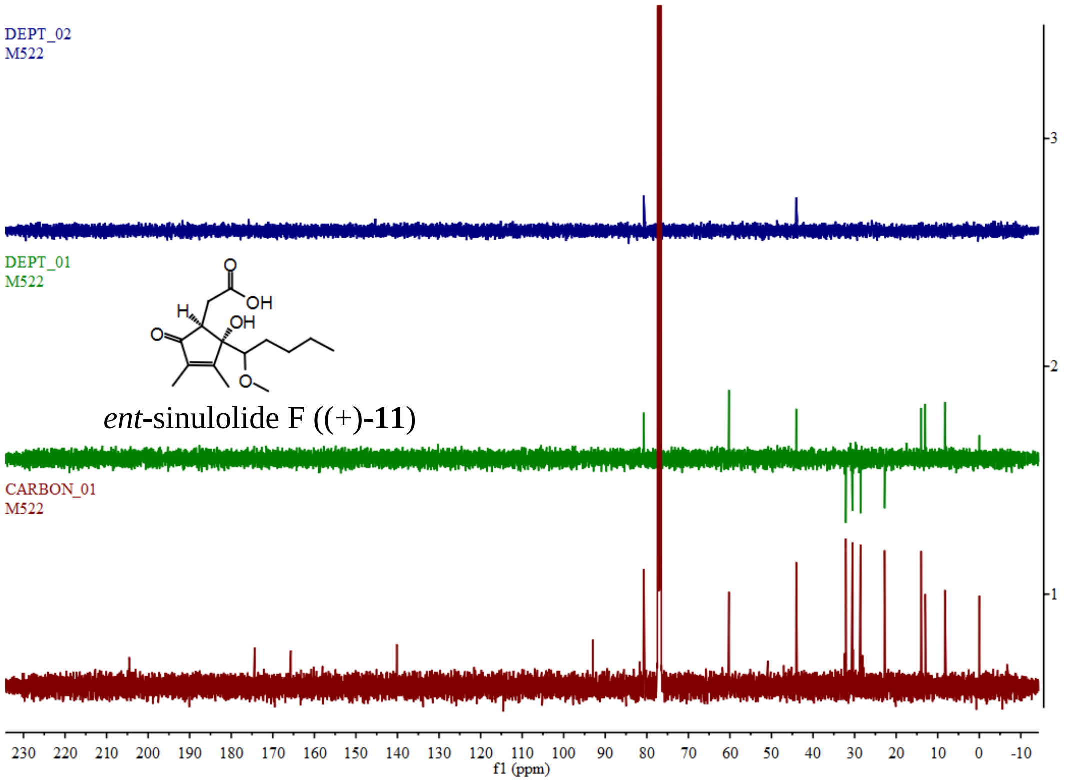

Figure S58. DEPT ($CDCl_3$, 125 MHz) spectrum of (+)-**11**

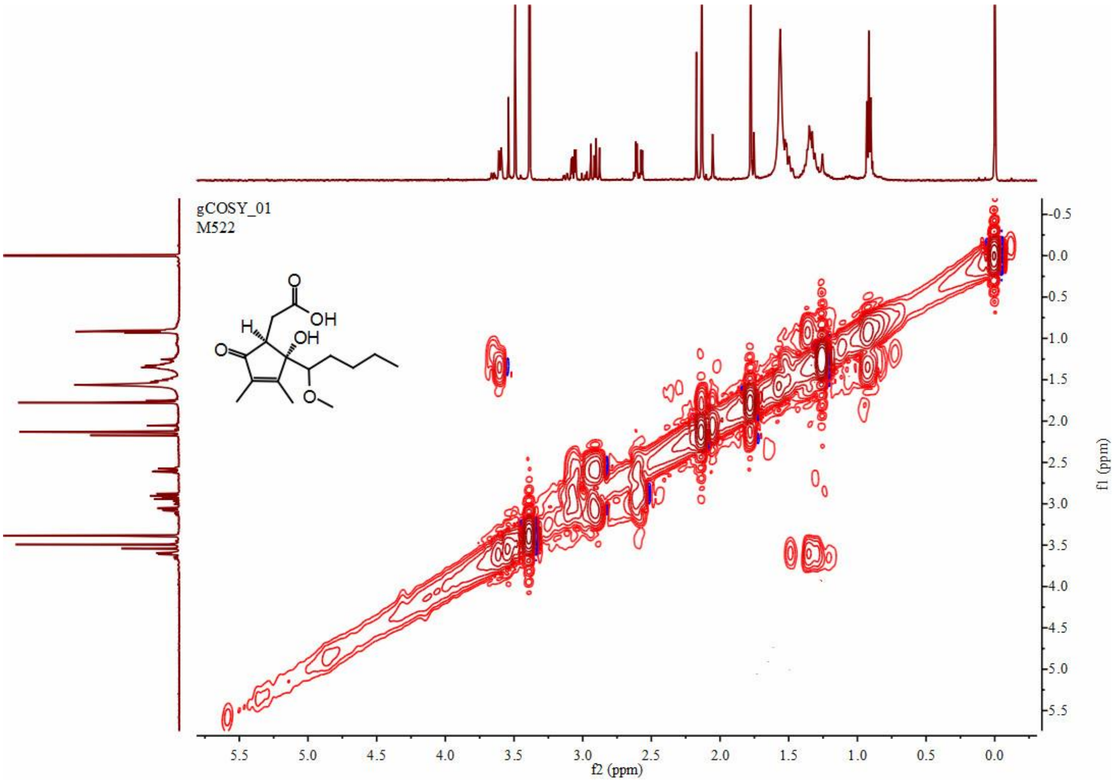

Figure S59. ^{1}H-^{1}H COSY ($CDCl_3$) spectrum of (+)-**11**

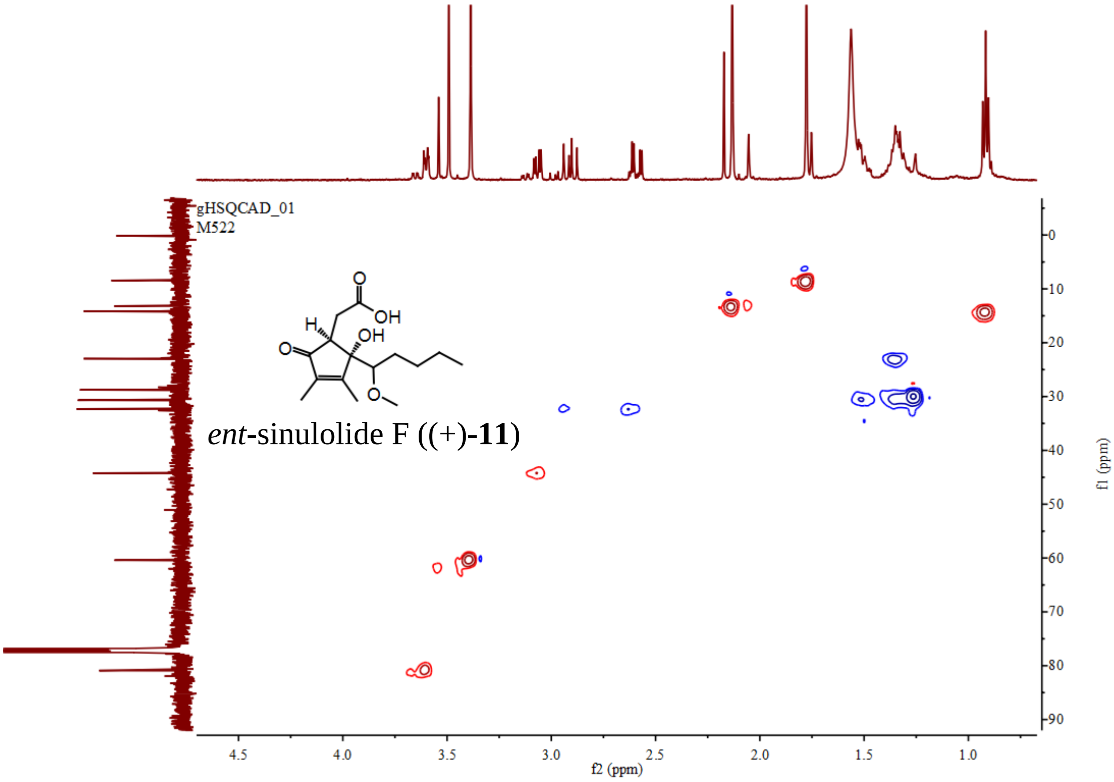

Figure S60. HSQC ($CDCl_3$) spectrum of (+)-**11**

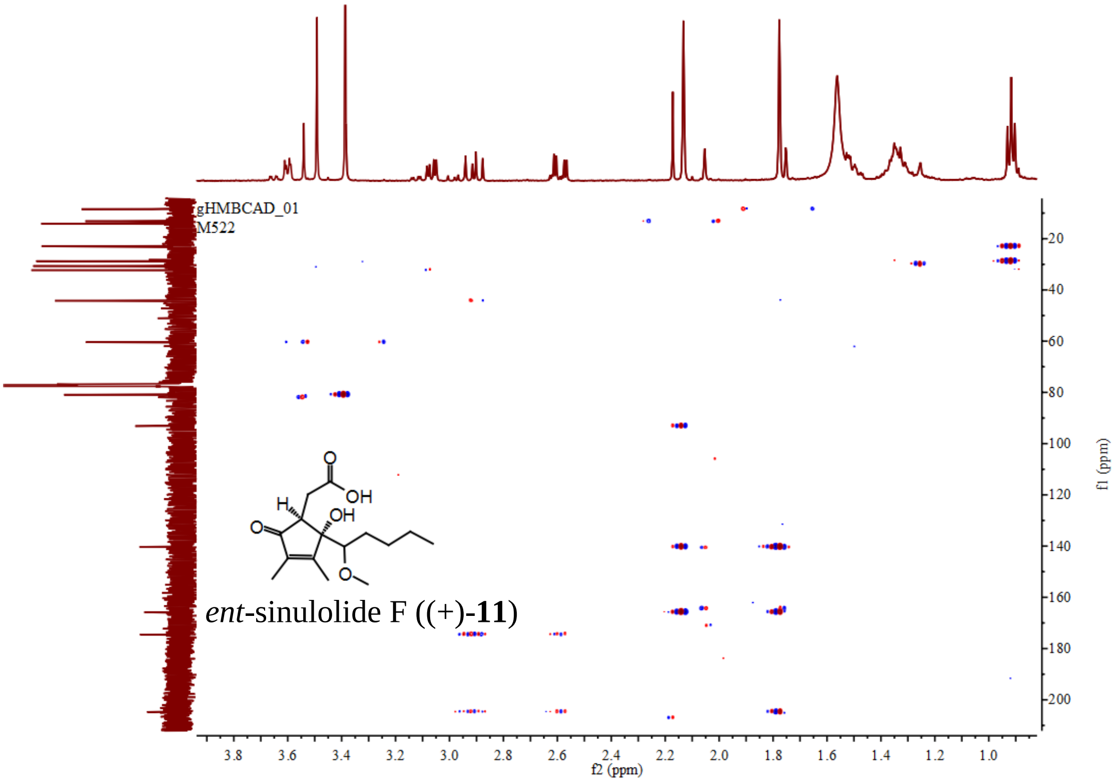

Figure S61. HMBC ($CDCl_3$) spectrum of (+)-**11**

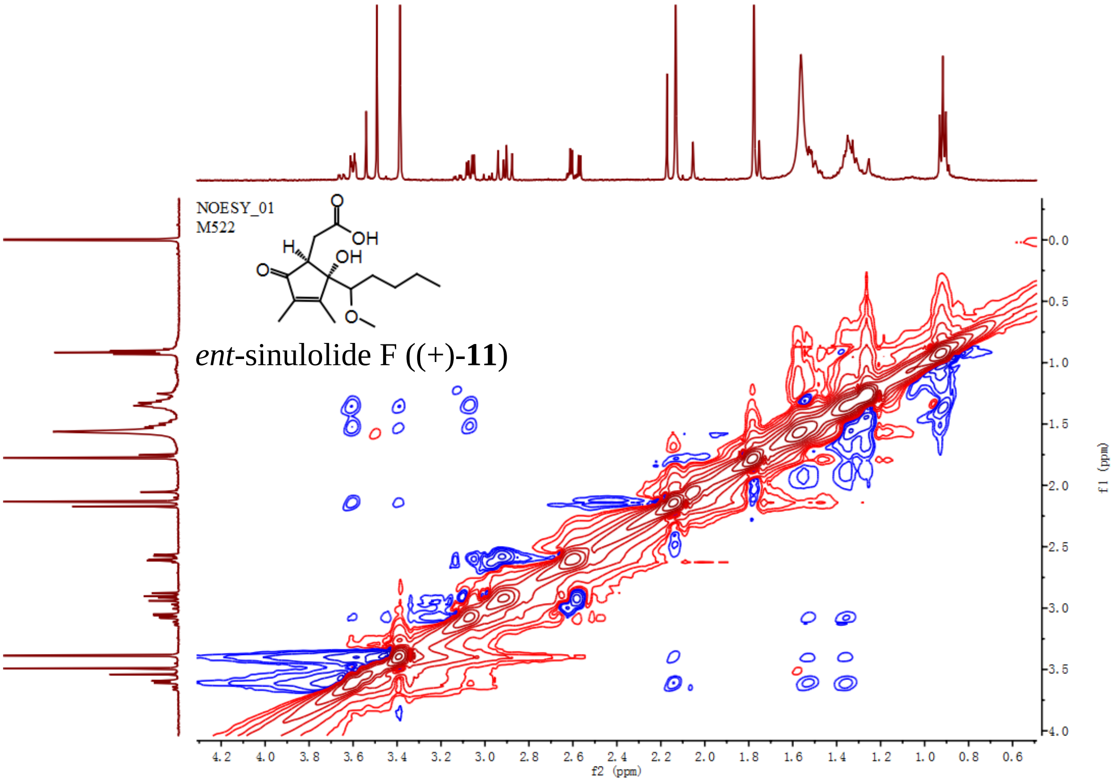

Figure S62. NOESY ($CDCl_3$) spectrum of (+)-**11**

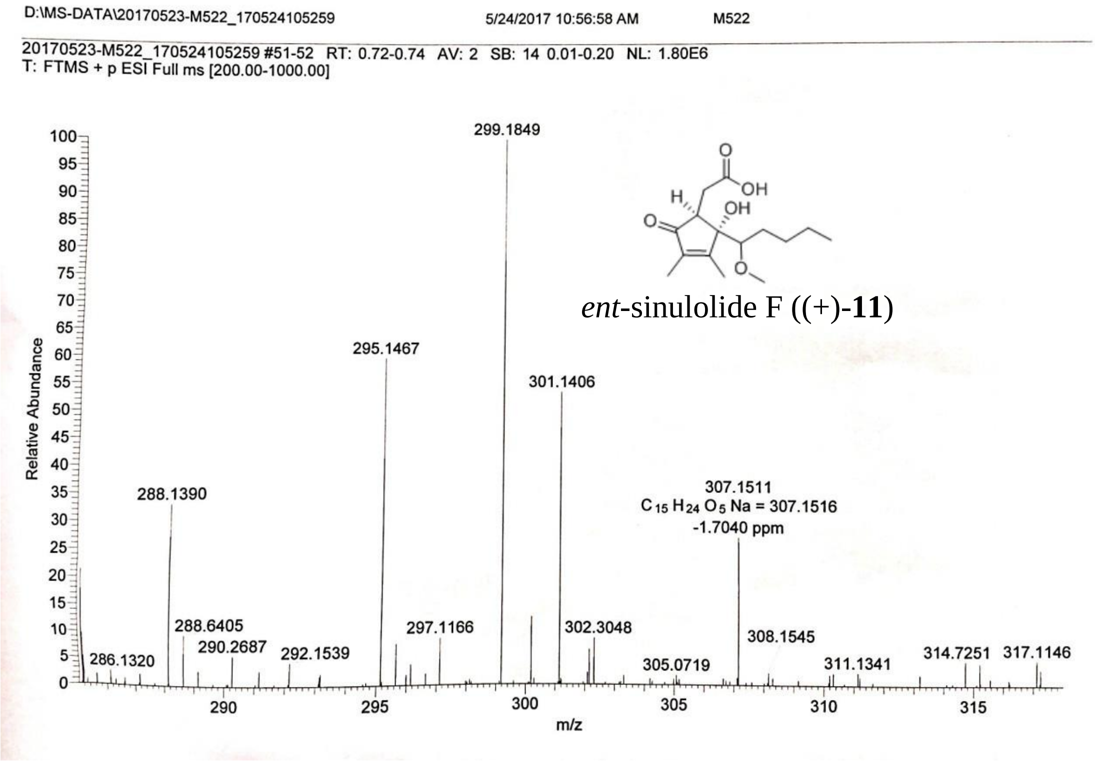

Figure S63. HRESIMS spectrum of **(+)-11**

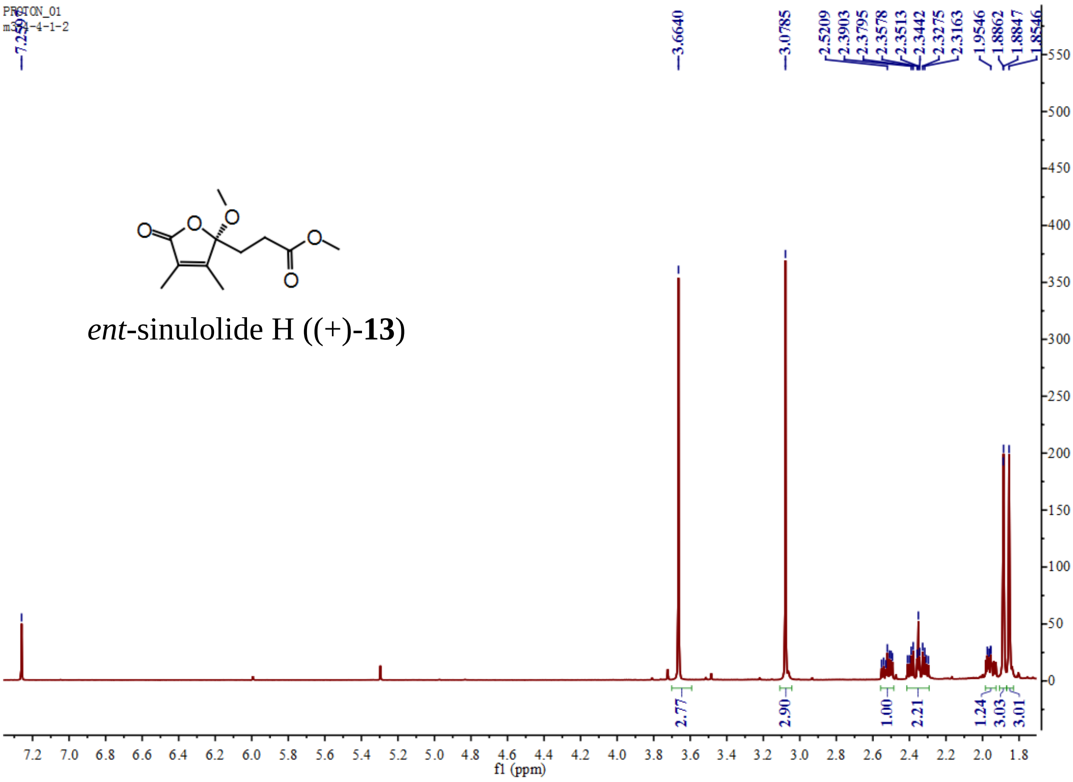

Figure S64. ^{1}H NMR ($CDCl_3$, 500 MHz) spectrum of (+)-**13**

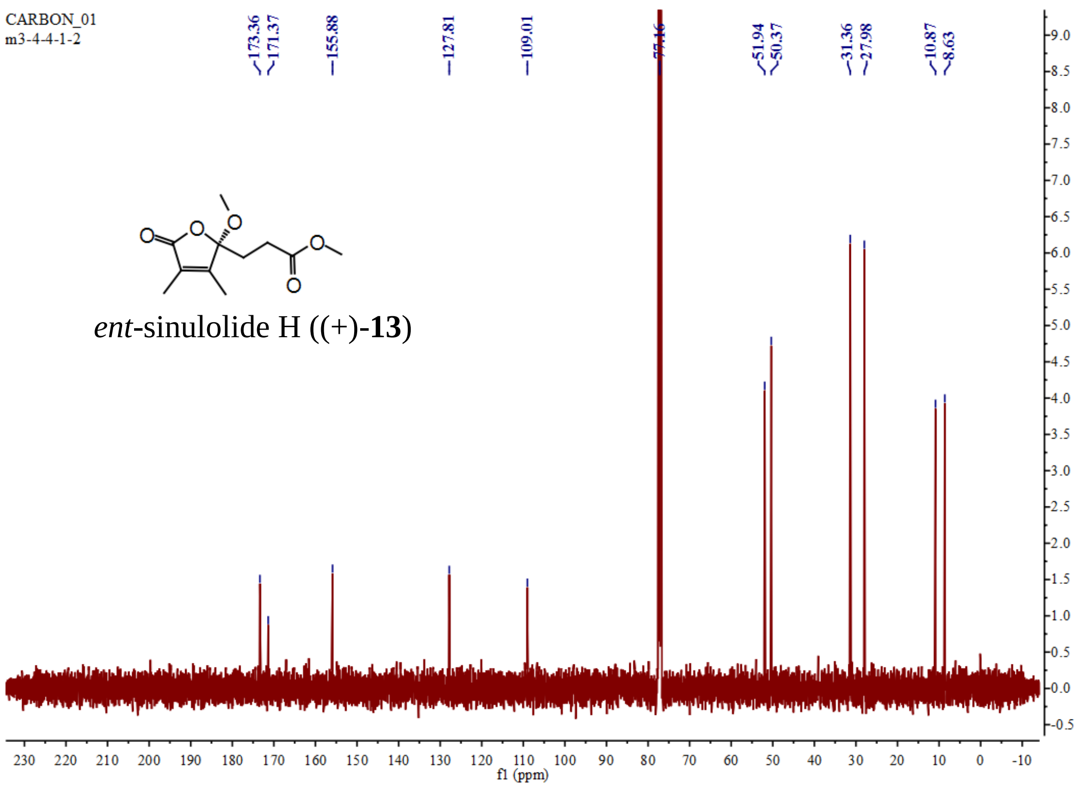

Figure S65. ^{13}C NMR ($CDCl_3$, 125 MHz) spectrum of (+)-**13**

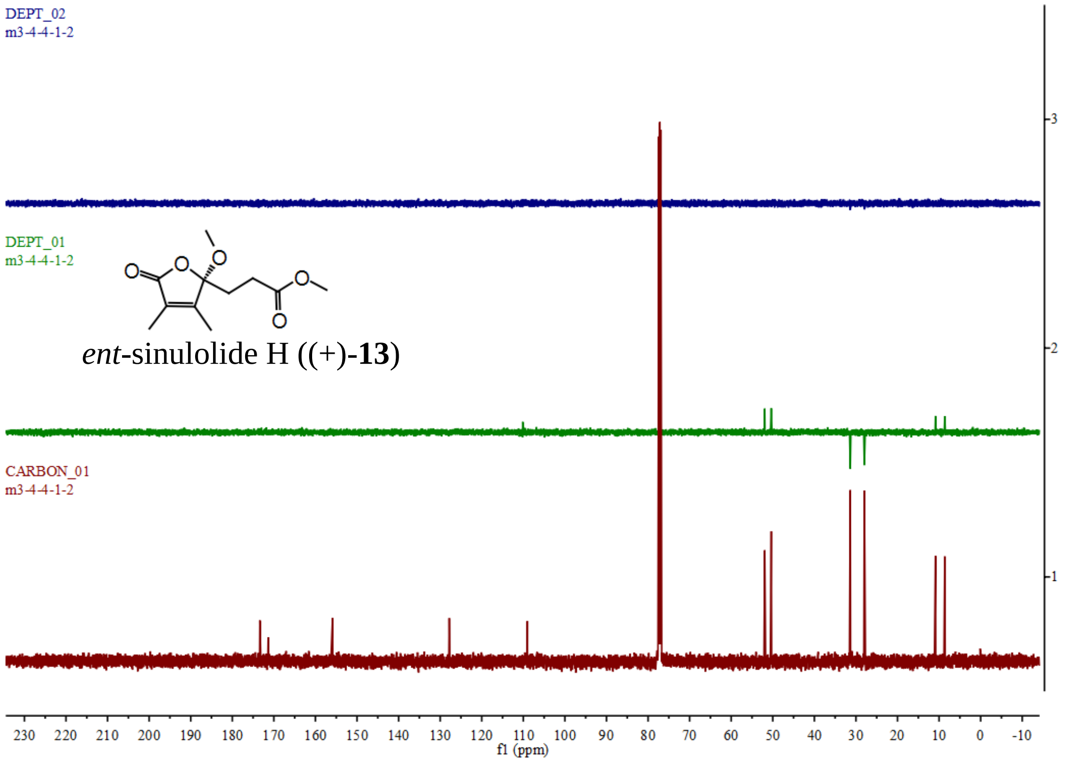

Figure S66. DEPT ($CDCl_3$, 125 MHz) spectrum of (+)-**13**

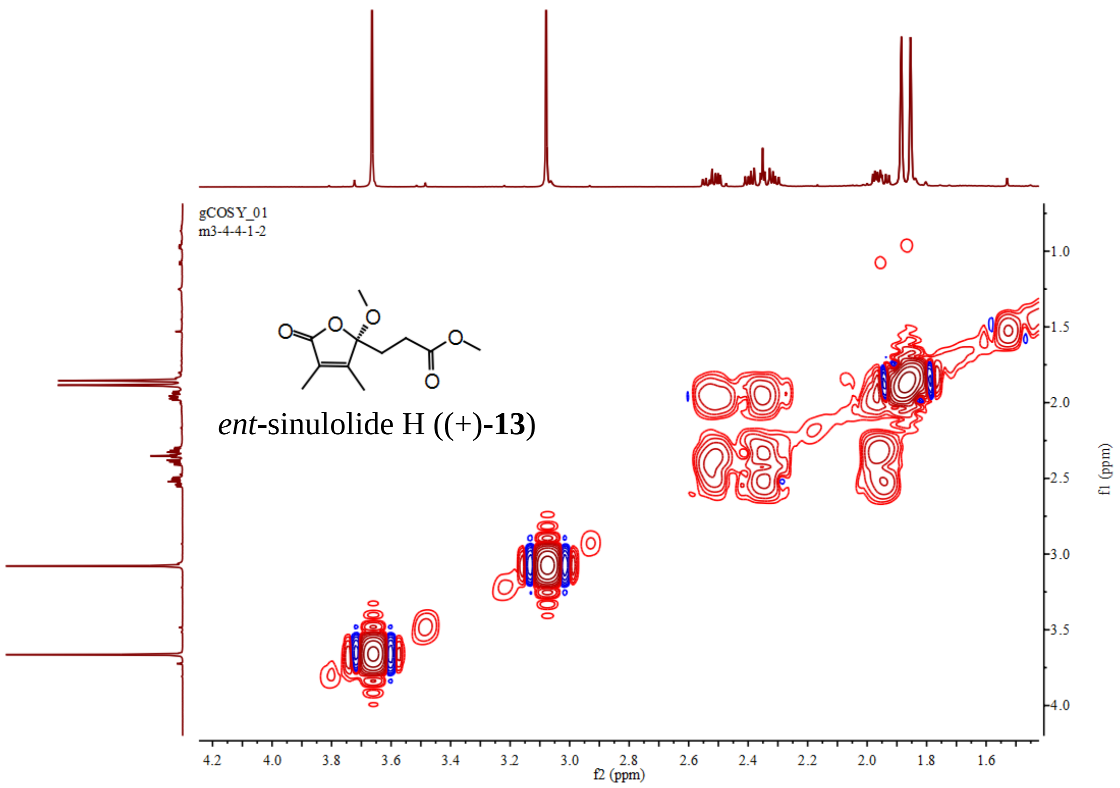

Figure S67. ^{1}H-^{1}H COSY ($CDCl_3$) spectrum of (+)-**13**

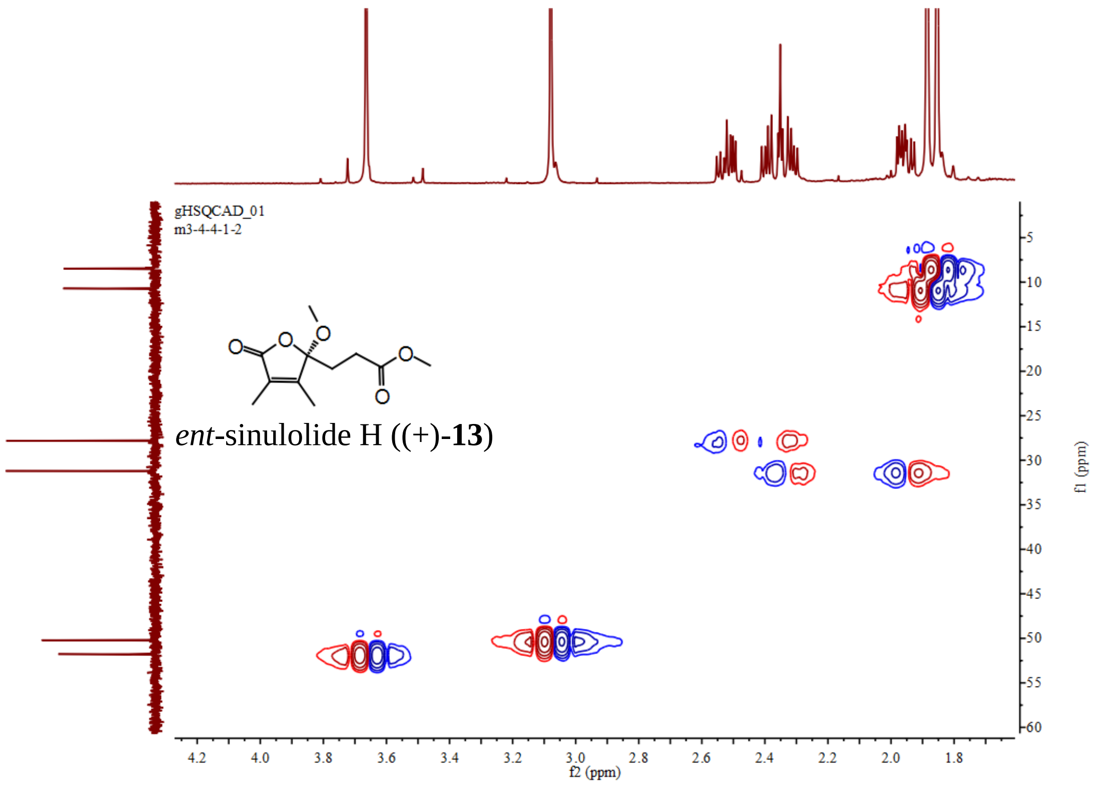

Figure S68. HSQC ($CDCl_3$) spectrum of (+)-**13**

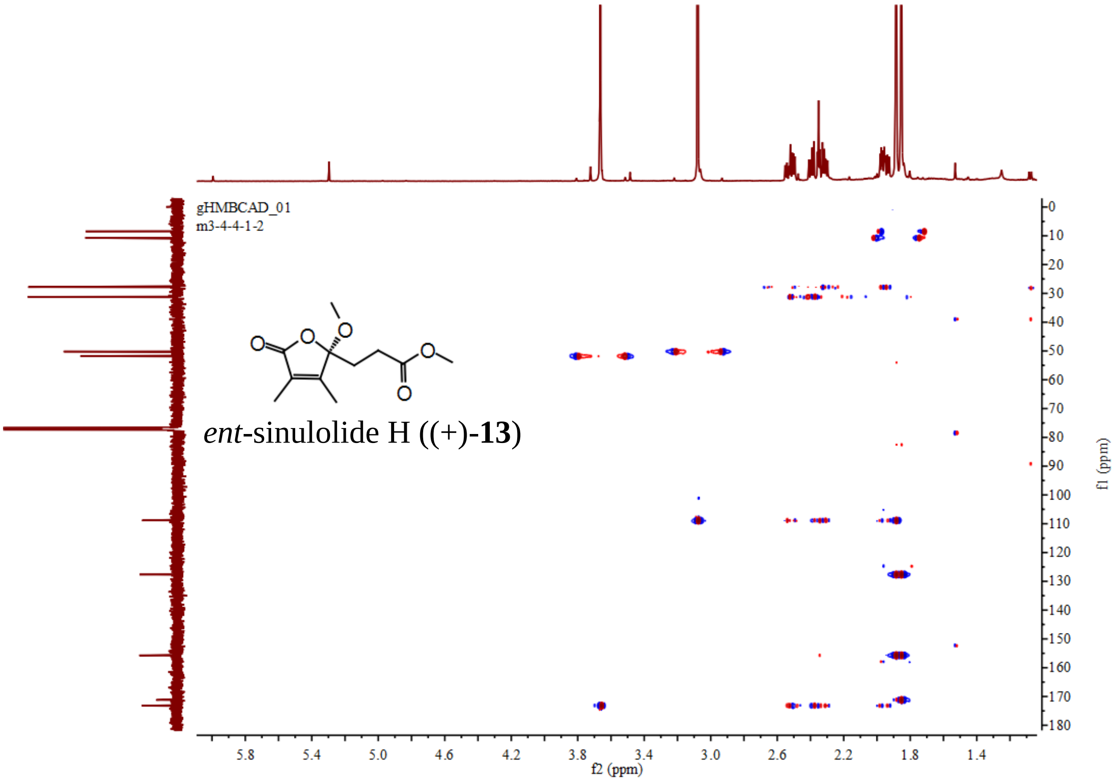

Figure S69. HMBC ($CDCl_3$) spectrum of (+)-**13**

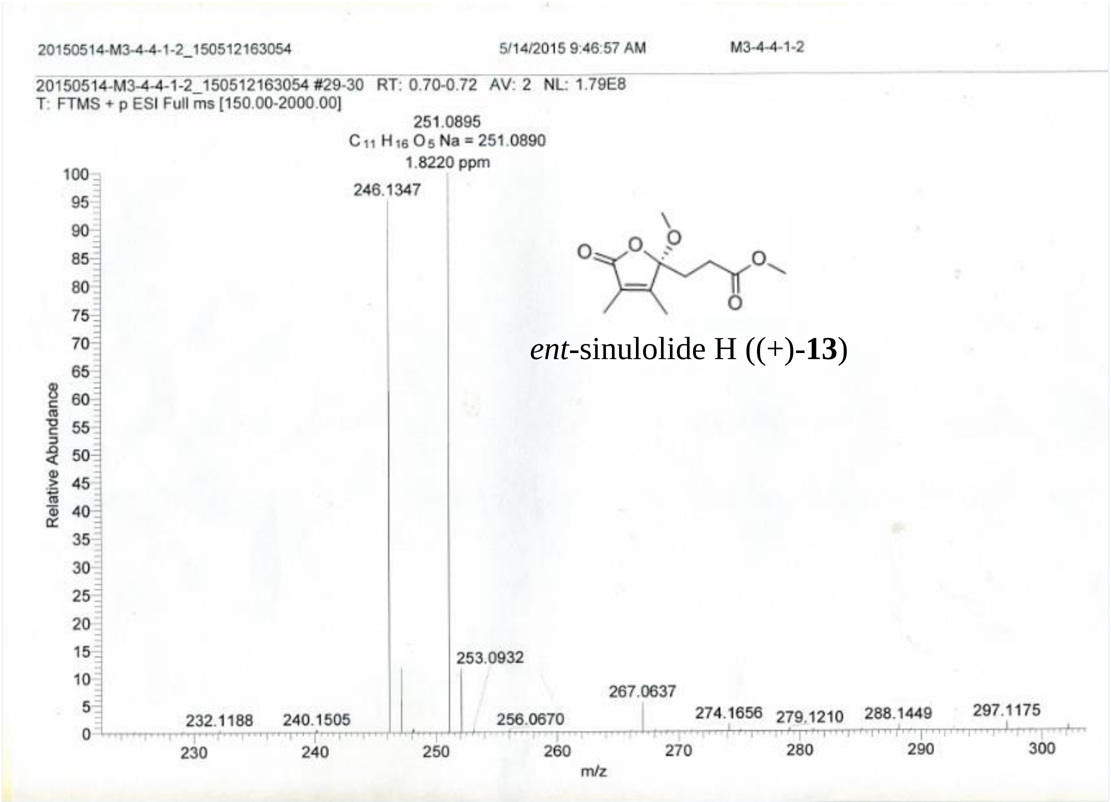

Figure S70. HRESIMS spectrum of (+)-**13**

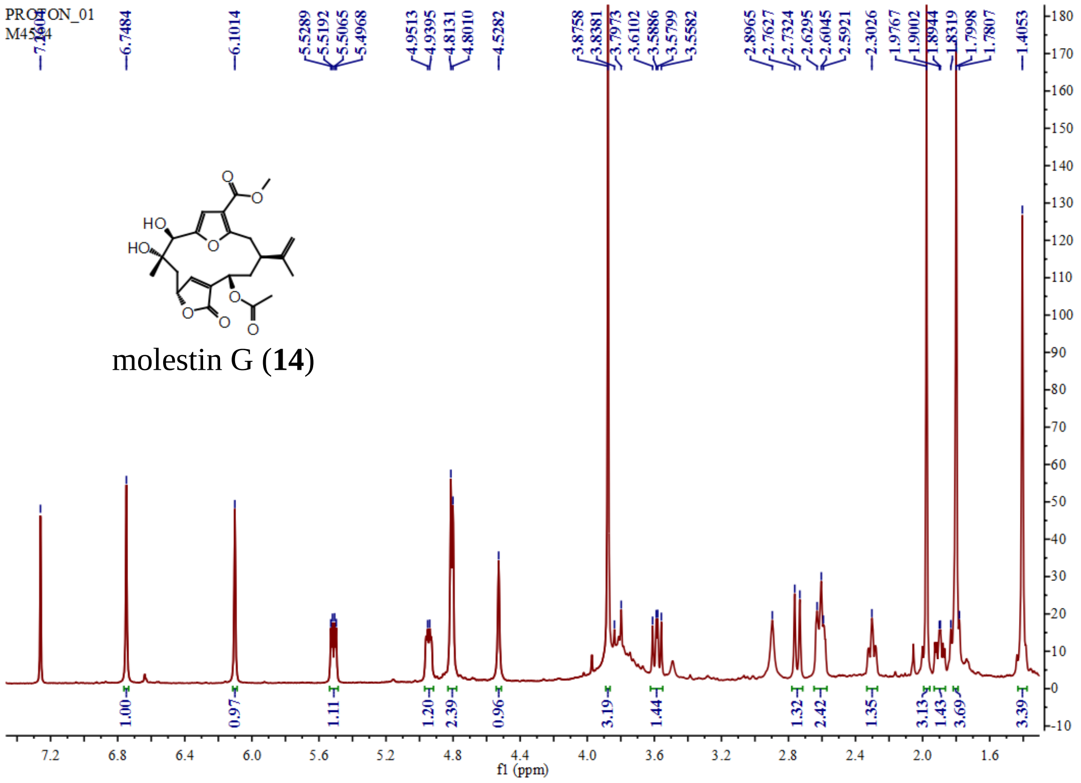

Figure S71. ^{1}H NMR ($CDCl_3$, 500 MHz) spectrum of **14**

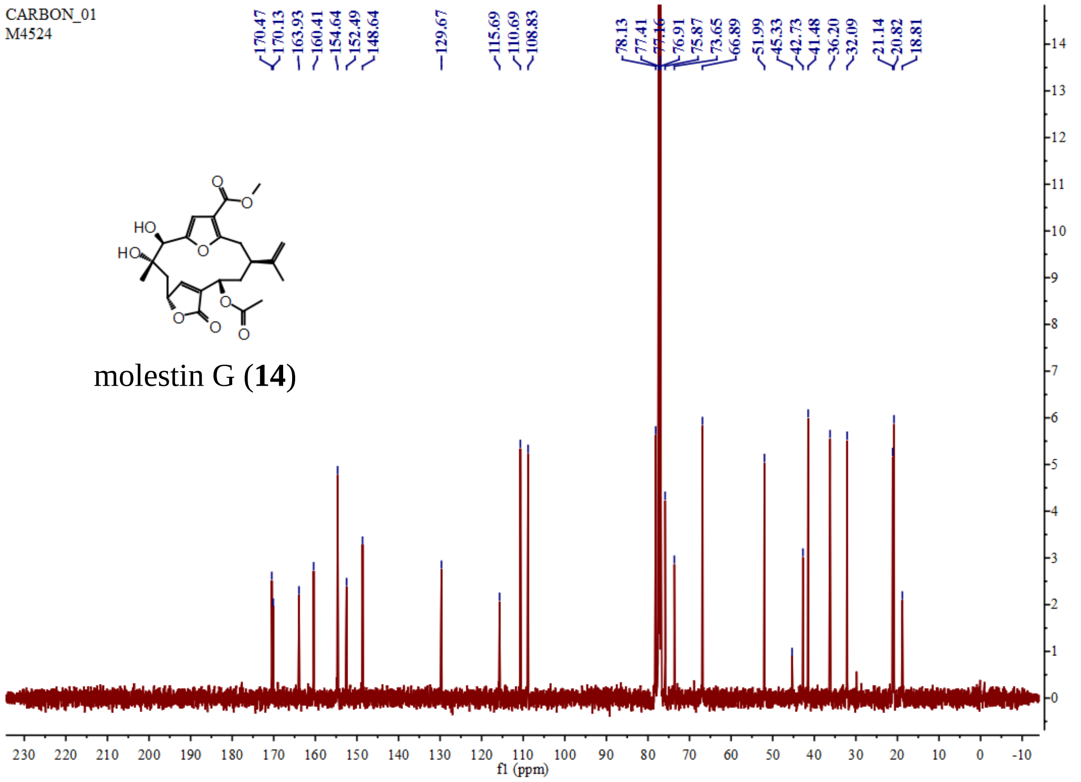

Figure S72. ^{13}C NMR ($CDCl_3$, 125 MHz) spectrum of **14**

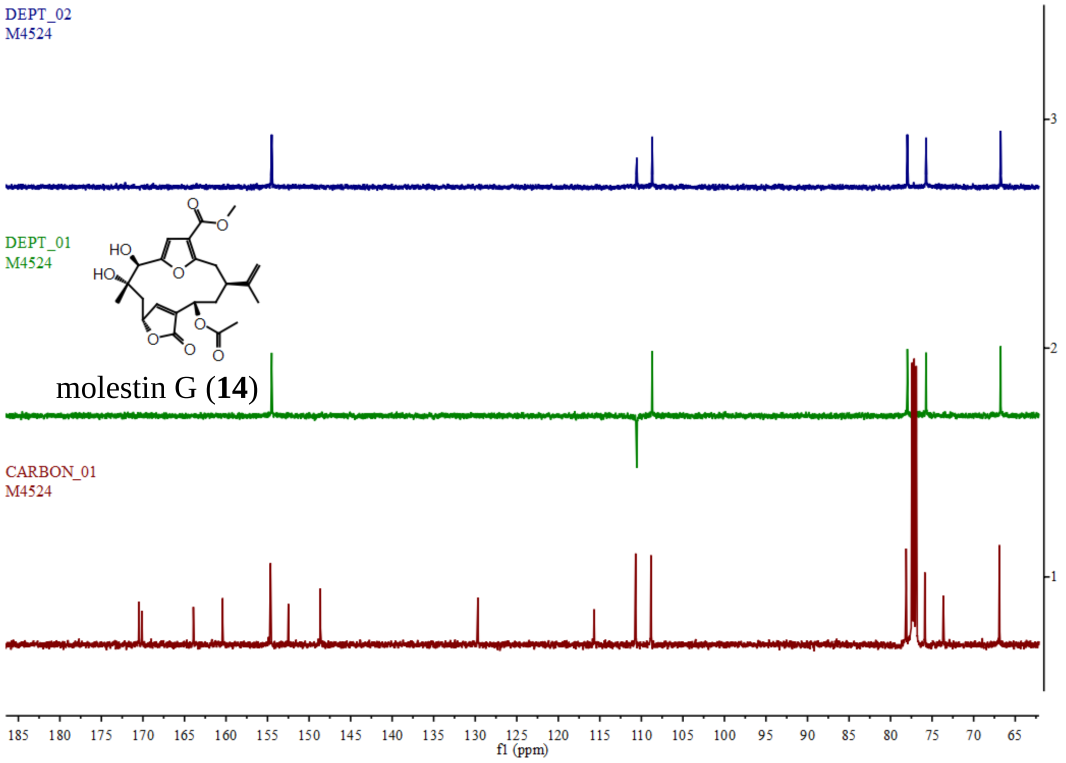

Figure S73. DEPT ($CDCl_3$, 125 MHz) spectrum of **14**

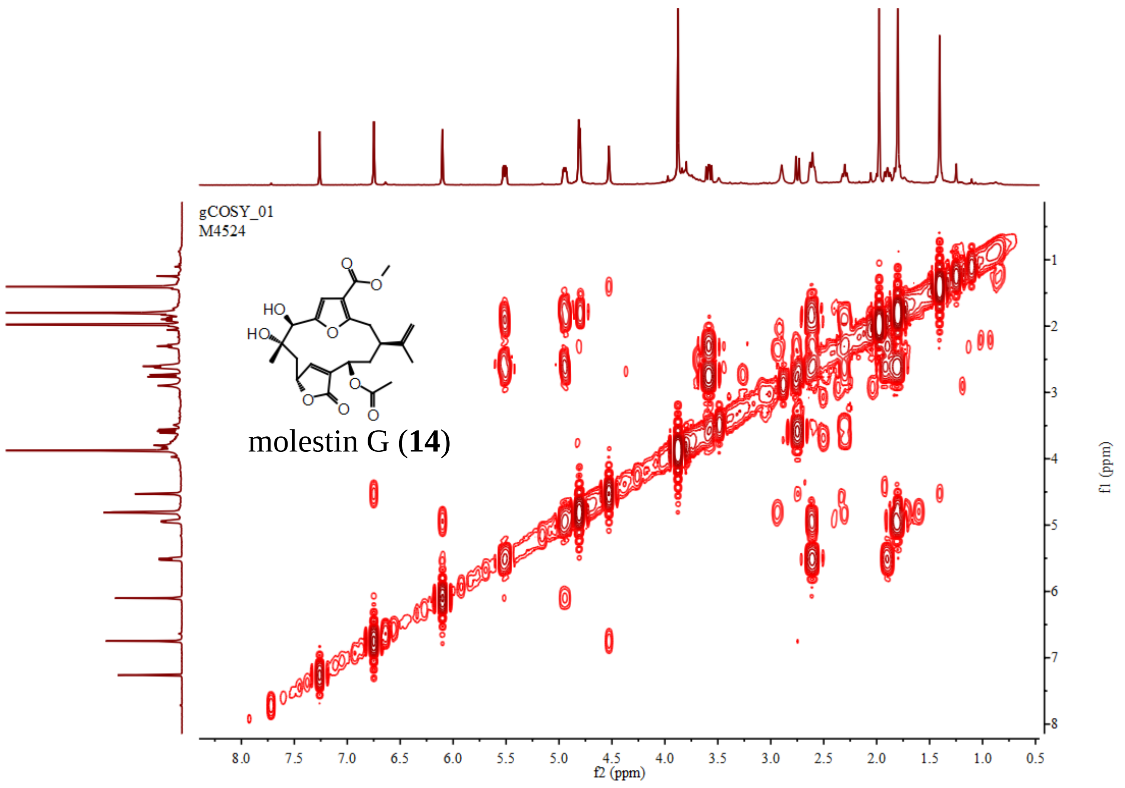

Figure S74. ^1H-^1H COSY ($CDCl_3$) spectrum of **14**

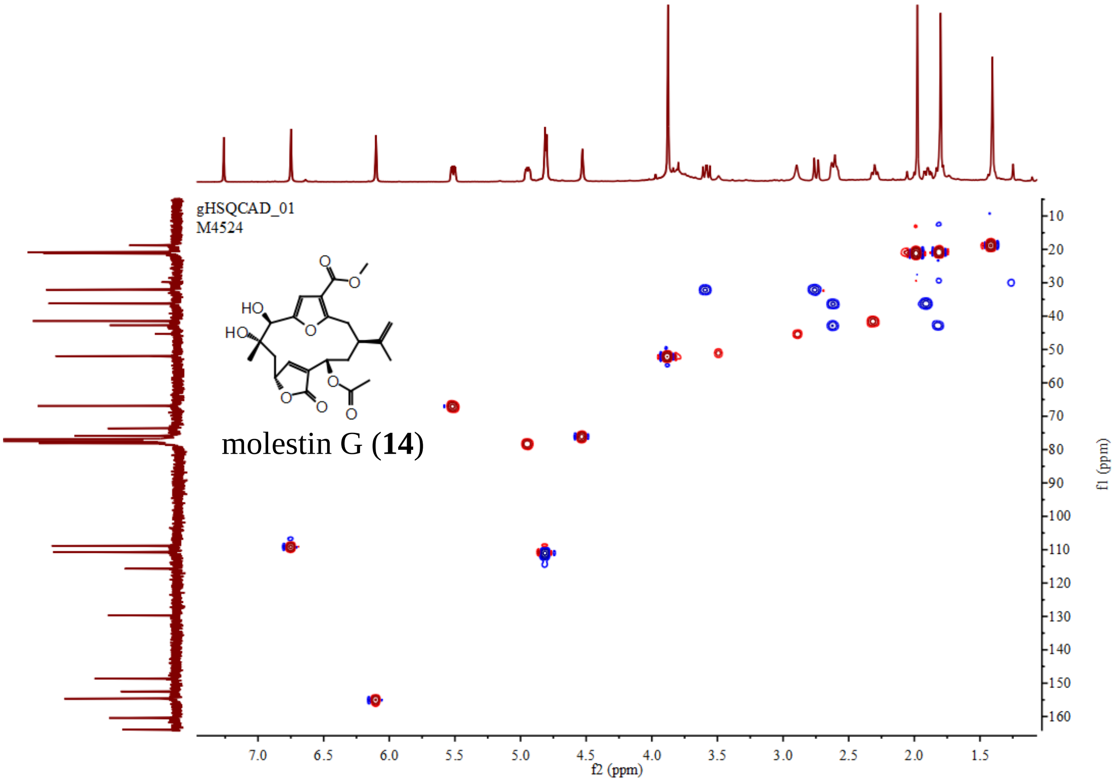

Figure S75. HSQC ($CDCl_3$) spectrum of **14**

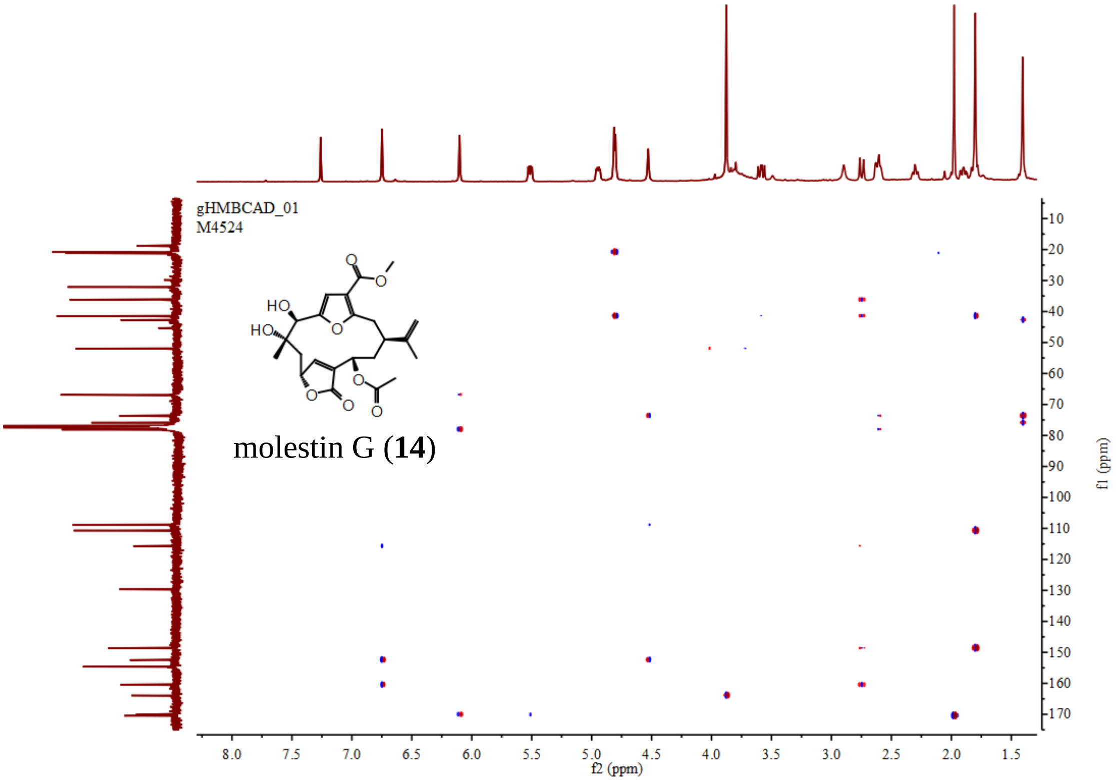

Figure S76. HMBC ($CDCl_3$) spectrum of **14**

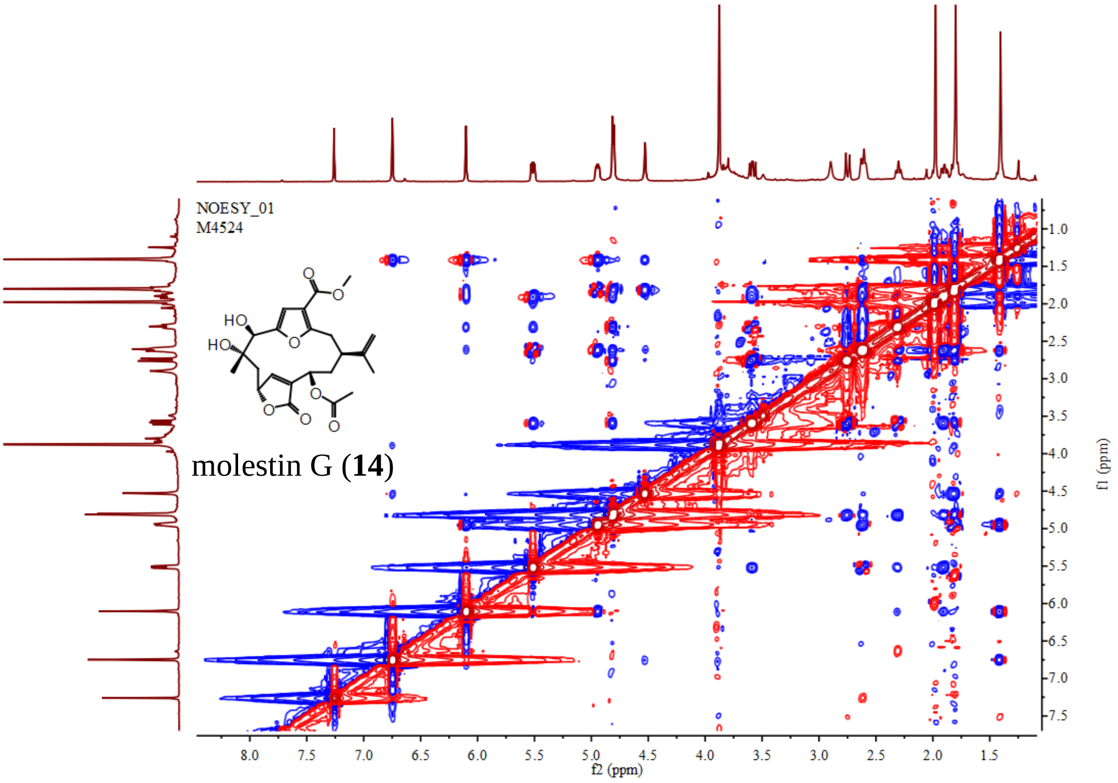

Figure S77. NOESY ($CDCl_3$) spectrum of **14**

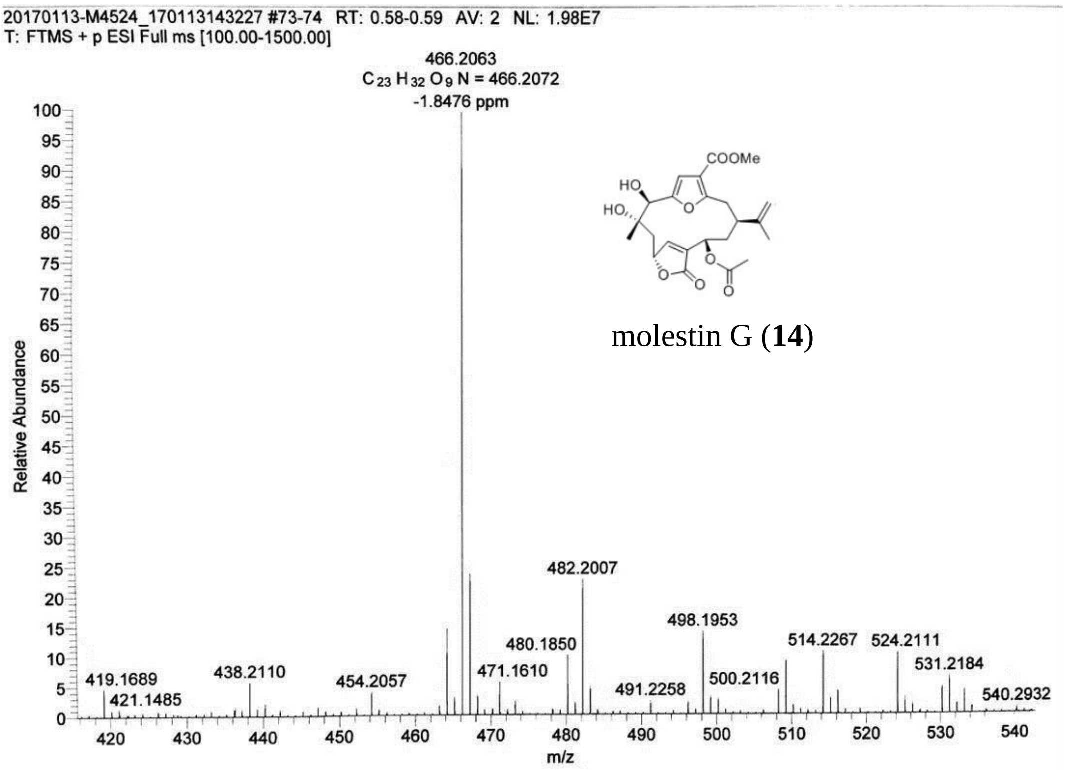

Figure S78. HRESIMS spectrum of **14**

Chiral HPLC chromatograms of compounds (±)-9–(±)-13

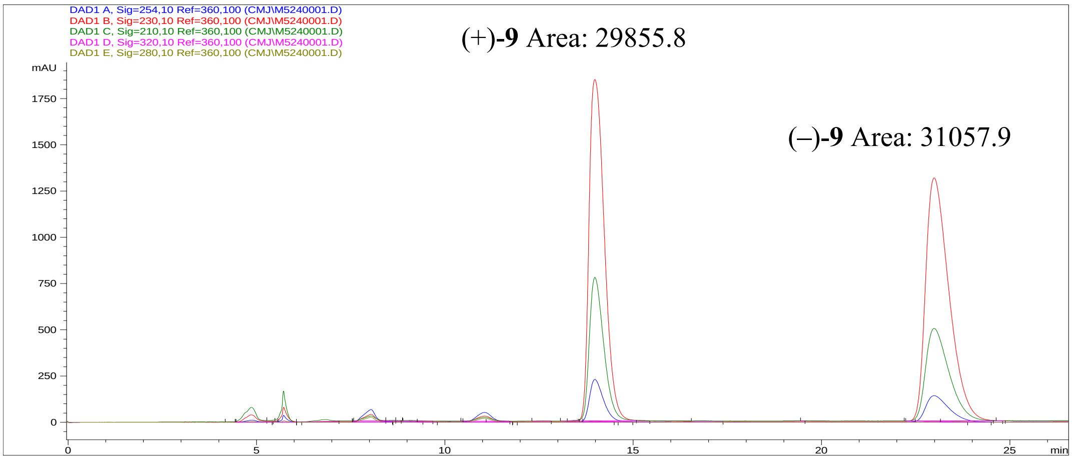

Figure S79. Chiral HPLC (n-hexane/i-PrOH 80:20) chromatogram of (+)-**9** and (–)-**9**

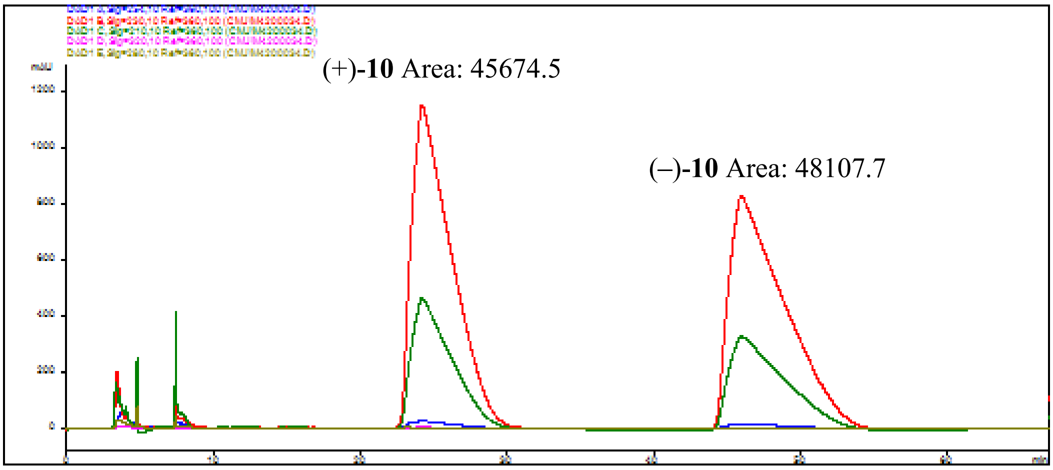

Figure S80. Chiral HPLC (n-hexane/i-PrOH 90:10) chromatogram of (+)-**10** and (–)-**10**

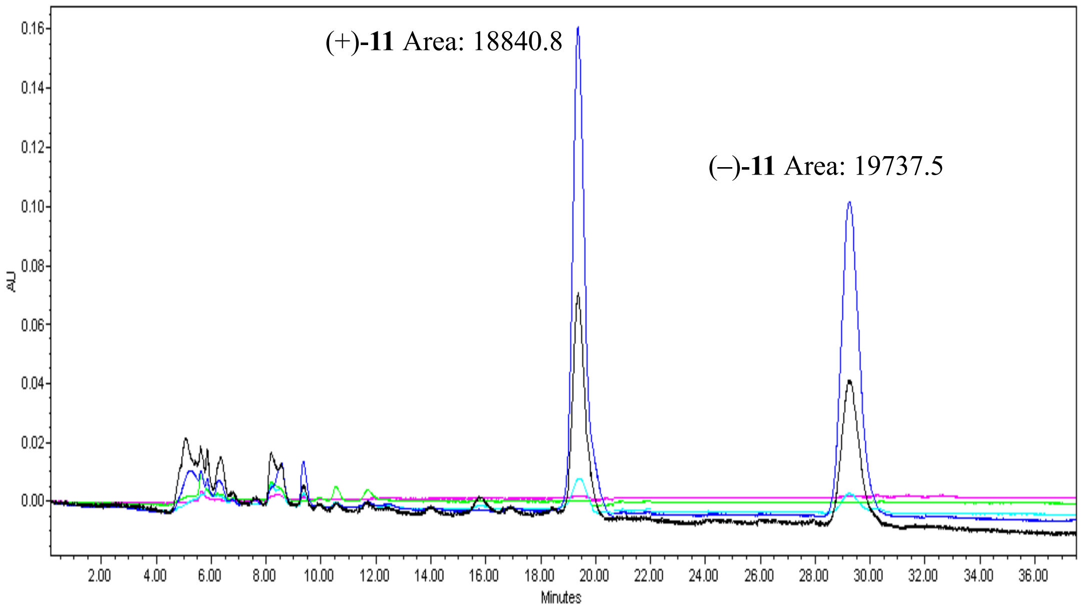

Figure S81. Chiral HPLC (n-hexane/i-PrOH 70:30) chromatogram of (+)-**11** and (–)-**11**

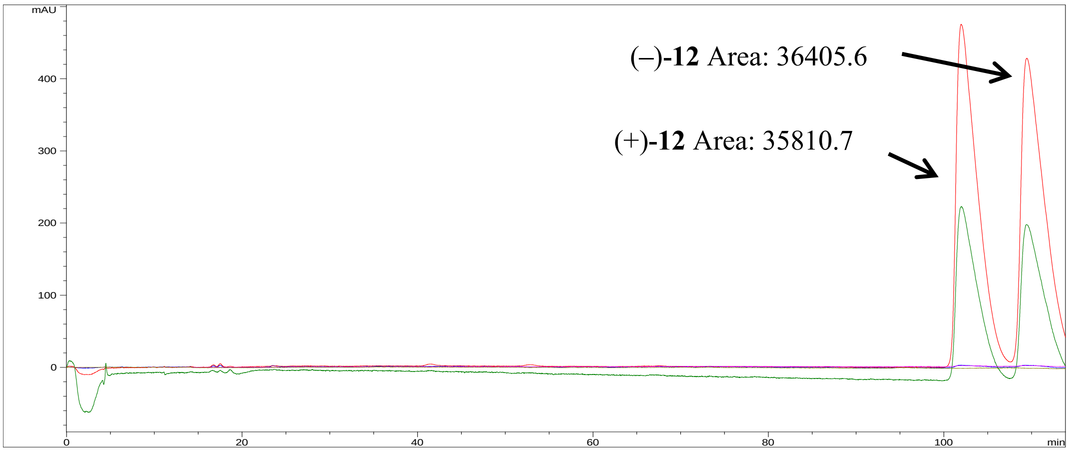

Figure S82. Chiral HPLC (n-hexane/i-PrOH 97:3) chromatogram of (+)-**12** and (–)-**12**

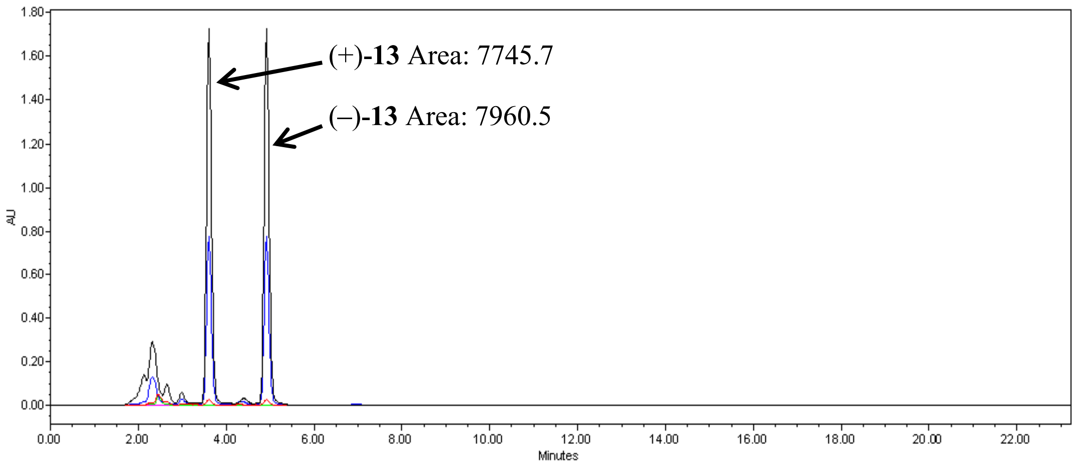

Figure S83. Chiral HPLC (n-hexane/i-PrOH 85:15) chromatogram of (+)-**13** and (–)-**13**

Computational details of compounds 1−5

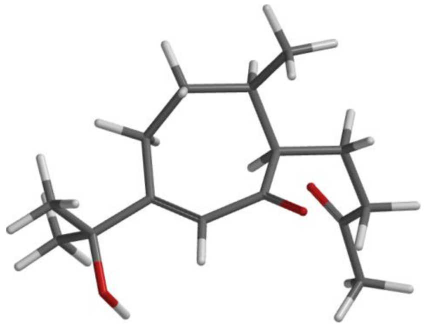

1a1 (29.01%)

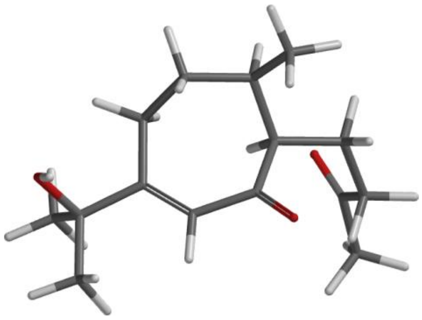

1a2 (22.88%)

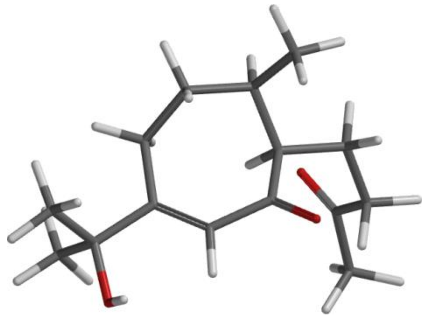

1a3 (17.95%)

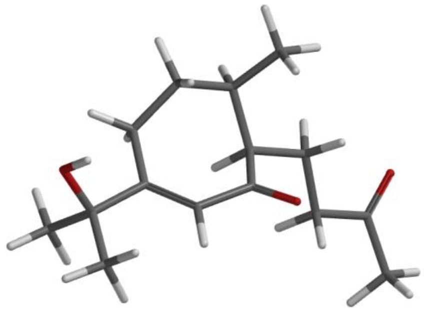

1a4 (15.44%)

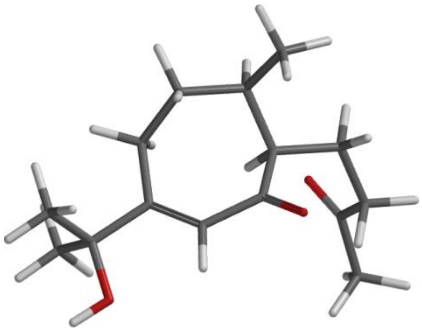

1a5 (11.11%)

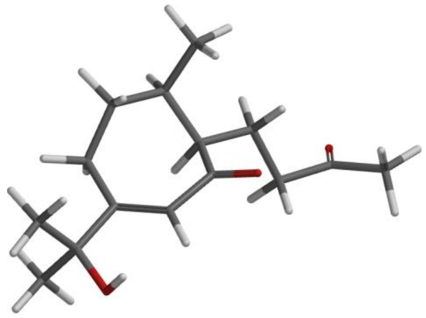

1a6 (2.41%)

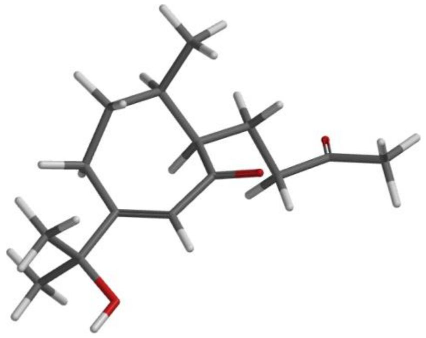

1a7 (1.21%)

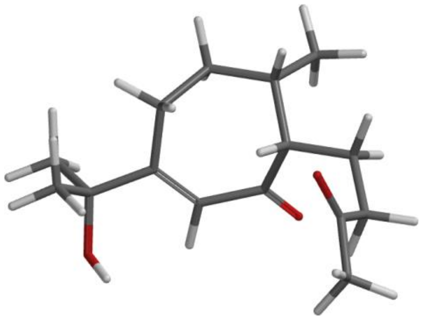

1b1 (29.01%)

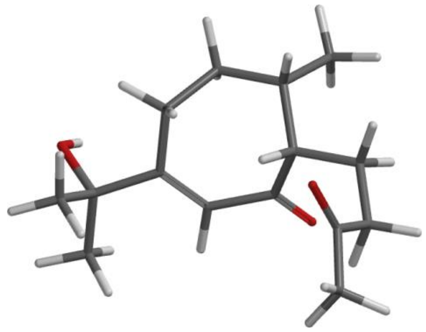

1b2 (22.88%)

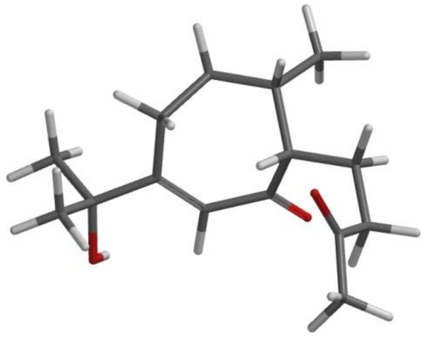

1b3 (17.94%)

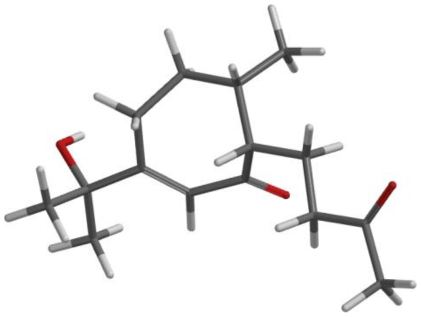

1b4 (15.44%)

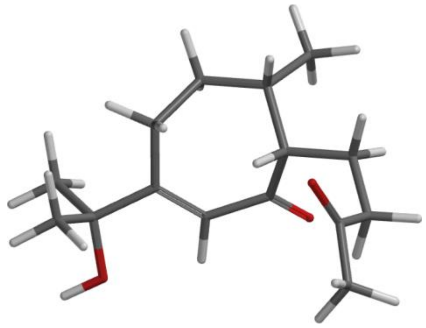

1b5 (11.11%)

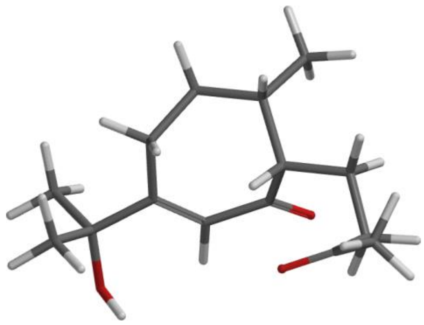

1b6 (2.41%)

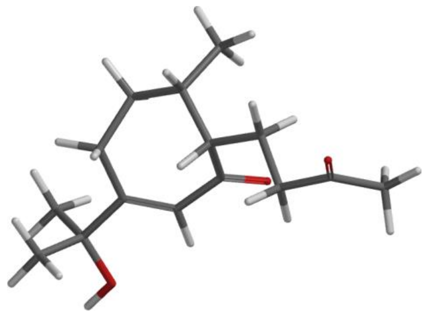

1b7 (1.21%)

Figure S84. Stable conformers of compound **1** with 1*R*,10*R* (**1a**) and 1*S*,10*S* (**1b**) configurations, respectively

Table S1. Important thermodynamic parameters (a.u.) of the optimized compound **1** at B3LYP/6-31G (d,p) level in the gas phase

conformations	E+ZPE	G	conformations	E+ZPE	G
**1a1**	-811.410490	-811.459589	**1b1**	-811.410490	-811.459589
**1a2**	-811.410136	-811.459365	**1b2**	-811.410136	-811.459365
**1a3**	-811.409851	-811.459136	**1b3**	-811.409851	-811.459136
**1a4**	-811.409589	-811.458994	**1b4**	-811.409589	-811.458994
**1a5**	-811.409391	-811.458684	**1b5**	-811.409391	-811.458684
**1a6**	-811.407779	-811.457242	**1b6**	-811.408095	-811.457244
**1a7**	-811.407362	-811.456592	**1b7**	-811.407362	-811.456592

Table S2. Optimized Z-Matrixes of compound **1** in the Gas Phase (Å) at B3LYP/6-31G (d,p) level

**1a1**				**1a2**				**1a3**			
C	1.023337	0.147292	1.078647	C	-1.027993	-0.149586	1.088848	C	-1.025667	-0.161352	1.074910
C	1.830307	0.148808	-0.004633	C	-1.834529	-0.144198	0.004799	C	-1.833222	-0.156484	-0.006703
C	1.558478	-0.662134	-1.260222	C	-1.540783	0.648764	-1.256820	C	-1.525886	0.618983	-1.276854
C	0.948037	-2.053296	-0.993259	C	-0.931494	2.041122	-0.995593	C	-0.932314	2.022118	-1.029401
C	-0.577786	-2.092974	-0.761177	C	0.591578	2.082353	-0.743762	C	0.588260	2.075424	-0.760881
C	-1.112665	-0.818509	-0.034408	C	1.121799	0.806018	-0.015899	C	1.120930	0.808164	-0.019976
C	-0.303465	-0.499357	1.223075	C	0.308290	0.482176	1.235634	C	0.299161	0.488213	1.228601
C	-2.622756	-0.898039	0.246420	C	2.630697	0.882948	0.272735	C	2.626883	0.896402	0.280214
C	-3.268264	0.438708	0.627618	C	3.274431	-0.458991	0.638285	C	3.272605	-0.443408	0.649821

C	-3.340457	1.474495	-0.485765	C	3.356067	-1.475174	-0.492797	C	3.367670	-1.456355	-0.483269
C	-3.857692	2.848731	-0.098447	C	3.852533	-2.861593	-0.122612	C	3.842542	-2.848692	-0.108629
O	-3.005870	1.225314	-1.633781	O	3.044000	-1.200762	-1.641209	O	3.080840	-1.174354	-1.636587
O	-0.757787	-0.697219	2.348953	O	0.760348	0.661557	2.365703	O	0.739310	0.690893	2.359290
C	3.147219	0.945945	0.027864	C	-3.173088	-0.907711	0.001018	C	-3.163074	-0.933663	0.022476
C	3.270711	1.839655	-1.224230	C	-3.530171	-1.582214	1.331824	C	-3.165310	-2.002547	-1.083689
C	4.352602	-0.000685	0.155157	C	-3.169233	-1.954039	-1.125226	C	-4.371851	0.015157	-0.119786
O	3.231977	1.779538	1.190905	O	-4.232820	0.012855	-0.351615	O	-3.316840	-1.679927	1.236265
C	-0.961980	-3.407599	-0.063997	C	0.968323	3.394424	-0.037967	C	0.945922	3.394558	-0.058383
H	1.358617	0.643857	1.984813	H	-1.338680	-0.647953	2.001784	H	-1.333119	-0.695248	1.968651
H	2.500747	-0.798975	-1.798973	H	-0.884121	0.068965	-1.922194	H	-0.849061	0.031220	-1.914044
H	0.915957	-0.090736	-1.946711	H	-2.483616	0.774343	-1.794631	H	-2.443284	0.727593	-1.860957
H	1.177168	-2.708571	-1.842452	H	-1.149517	2.688567	-1.853530	H	-1.468640	2.475202	-0.185535
H	1.461084	-2.488243	-0.125709	H	-1.454790	2.487326	-0.139479	H	-1.143584	2.655688	-1.899375
H	-1.063825	-2.076037	-1.746961	H	1.089815	2.068067	-1.723302	H	1.096666	2.060063	-1.735112
H	-0.946758	0.009328	-0.732593	H	0.957224	-0.019952	-0.716863	H	0.968938	-0.024606	-0.715693
H	-2.804059	-1.604148	1.060859	H	3.130691	1.280986	-0.617244	H	3.132375	1.297058	-0.605461
H	-3.119536	-1.285441	-0.650241	H	2.808671	1.579302	1.096260	H	2.793266	1.593167	1.105794
H	-2.768526	0.886434	1.496977	H	4.307465	-0.295036	0.980425	H	4.302439	-0.276674	1.000305
H	-4.303485	0.270058	0.960498	H	2.769397	-0.921652	1.496608	H	2.761876	-0.909549	1.502785
H	-4.031818	3.448571	-0.993340	H	4.752066	-2.807608	0.500387	H	4.081880	-3.417351	-1.008862

H	-3.117444	3.353079	0.535212	H	3.085636	-3.376868	0.469315	H	4.712595	-2.807590	0.555672
H	-4.779269	2.772970	0.489162	H	4.054954	-3.439766	-1.025798	H	3.045988	-3.366664	0.440368
H	3.303306	1.261604	-2.151515	H	-2.803332	-2.351708	1.608191	H	-3.050872	-1.557899	-2.075628
H	4.191281	2.425766	-1.151233	H	-3.587298	-0.857353	2.151433	H	-2.348692	-2.711143	-0.919320
H	2.423373	2.532644	-1.290420	H	-4.509814	-2.056680	1.229270	H	-4.110800	-2.551707	-1.053695
H	4.403010	-0.707190	-0.677263	H	-2.391794	-2.703771	-0.950062	H	-4.376206	0.556523	-1.069292
H	4.286253	-0.562054	1.090956	H	-4.141938	-2.453689	-1.158566	H	-5.292420	-0.572884	-0.059007
H	5.275018	0.587320	0.167879	H	-2.994676	-1.489642	-2.098426	H	-4.378254	0.756216	0.688477
H	2.539892	2.454167	1.143316	H	-4.340744	0.647988	0.370373	H	-3.500602	-1.068767	1.963141
H	-0.597172	-3.430795	0.969345	H	2.050348	3.548164	-0.000923	H	2.026329	3.555632	-0.009050
H	-2.044266	-3.561864	-0.042000	H	0.533514	4.247452	-0.571495	H	0.511978	4.241752	-0.601950
H	-0.519863	-4.259055	-0.594083	H	0.589743	3.415523	0.990616	H	0.554819	3.417965	0.965337
**1a4**				**1a5**				**1a6**			
C	-1.222844	-0.604119	0.817861	C	-1.020374	-0.153679	1.082781	C	1.208141	-0.678231	-0.730502
C	-2.163643	-0.208703	-0.067009	C	-1.823591	-0.150371	-0.001829	C	2.170177	-0.188520	0.079432
C	-1.988169	0.970977	-1.008415	C	-1.542726	0.649962	-1.262661	C	1.988856	1.050543	0.941301
C	-1.249028	2.171586	-0.384145	C	-0.934572	2.043758	-1.003003	C	1.208999	2.190223	0.252655
C	0.293324	2.093430	-0.370597	C	0.590292	2.083463	-0.762622	C	-0.331559	2.077518	0.289751
C	0.828514	0.635522	-0.194724	C	1.119160	0.811685	-0.027600	C	-0.835177	0.599060	0.243839
C	0.156519	-0.076557	0.979930	C	0.302340	0.501066	1.227937	C	-0.180653	-0.186314	-0.895751
C	2.361945	0.577683	-0.116861	C	2.627717	0.887818	0.261743	C	-2.368032	0.498413	0.207597
C	2.927730	-0.835429	-0.275662	C	3.262554	-0.454687	0.640046	C	-2.889029	-0.909384	0.574775

C	4.447463	-0.889062	-0.369400	C	3.342552	-1.479645	-0.482973	C	-4.410337	-0.948204	0.508910
C	5.083306	-2.262323	-0.247119	C	3.799679	-2.874284	-0.094228	C	-5.035414	-1.421527	-0.788287
O	5.124674	0.112221	-0.531868	O	3.058572	-1.206204	-1.639056	O	-5.089998	-0.593514	1.459024
O	0.753773	-0.284742	2.033832	O	0.748740	0.715974	2.353859	O	-0.803360	-0.469871	-1.916881
C	-3.540508	-0.897877	-0.134229	C	-3.141002	-0.934069	0.022038	C	3.553159	-0.866021	0.129261
C	-3.764164	-1.987918	0.921673	C	-3.226189	-1.882240	-1.192376	C	3.842877	-1.350663	1.559988
C	-3.758341	-1.485516	-1.538146	C	-4.347275	0.028166	0.063194	C	4.661473	0.084591	-0.370940
O	-4.570730	0.107844	0.010462	O	-3.147363	-1.727321	1.219090	O	3.589460	-2.058431	-0.663406
C	0.856713	3.082326	0.661859	C	0.971968	3.400342	-0.068481	C	-0.943889	2.967307	-0.803281
H	-1.441836	-1.384719	1.539520	H	-1.346175	-0.676580	1.975571	H	1.426012	-1.534959	-1.360414
H	-1.474252	0.648219	-1.926384	H	-0.890867	0.073232	-1.935660	H	1.497907	0.775721	1.886640
H	-2.984255	1.295749	-1.318447	H	-2.477413	0.781116	-1.815622	H	2.971437	1.429330	1.233591
H	-1.615225	2.301465	0.642758	H	-1.158183	2.692916	-1.858433	H	1.544319	2.251193	-0.790666
H	-1.535358	3.082317	-0.923877	H	-1.451655	2.485352	-0.141218	H	1.486971	3.143569	0.717997
H	0.648859	2.403956	-1.363849	H	1.081722	2.062184	-1.745655	H	-0.667474	2.459305	1.264599
H	0.513962	0.091907	-1.096930	H	0.954660	-0.019384	-0.722387	H	-0.490604	0.132302	1.176472
H	2.782530	1.217464	-0.900291	H	3.131708	1.276163	-0.630364	H	-2.790403	1.219405	0.917403
H	2.701641	0.984977	0.838678	H	2.805767	1.589739	1.080497	H	-2.722385	0.769776	-0.791323
H	2.608797	-1.482408	0.551136	H	4.295849	-0.294869	0.983755	H	-2.462370	-1.649616	-0.110090
H	2.542812	-1.313068	-1.191456	H	2.751587	-0.907405	1.500033	H	-2.591791	-1.157131	1.599721
H	4.539160	-3.008987	-0.836055	H	2.996925	-3.375660	0.461098	H	-6.108890	-1.225268	-0.786873

H	6.127466	-2.221133	-0.561956	H	4.030752	-3.455475	-0.988624	H	-4.558906	-0.941764	-1.650605
H	5.039218	-2.585906	0.800496	H	4.670851	-2.837510	0.568900	H	-4.864646	-2.500433	-0.896216
H	-3.066532	-2.822084	0.802540	H	-3.219107	-1.343391	-2.143944	H	3.839063	-0.524340	2.275578
H	-3.656049	-1.595580	1.938893	H	-2.387843	-2.584335	-1.178363	H	3.091025	-2.084490	1.863699
H	-4.781323	-2.373745	0.812868	H	-4.158063	-2.457880	-1.145210	H	4.824983	-1.831216	1.586866
H	-3.017779	-2.263271	-1.747659	H	-4.382706	0.688729	-0.807586	H	4.760695	0.981768	0.245269
H	-4.758506	-1.924974	-1.595503	H	-5.282188	-0.545899	0.074369	H	5.617790	-0.446491	-0.353463
H	-3.684802	-0.713776	-2.307828	H	-4.304750	0.641536	0.967485	H	4.460178	0.405676	-1.400003
H	-4.542971	0.448111	0.915875	H	-4.013656	-2.146842	1.300882	H	3.612749	-1.817555	-1.599904
H	0.410767	4.072635	0.514803	H	2.054474	3.552107	-0.039356	H	-0.519326	3.976248	-0.749450
H	0.628139	2.761899	1.684897	H	0.535449	4.250590	-0.605295	H	-0.734602	2.568756	-1.802557
H	1.940889	3.194243	0.578258	H	0.600069	3.428262	0.962195	H	-2.028098	3.062005	-0.698650
**1a7**				**1b1**				**1b2**			
C	1.205440	-0.666021	-0.751315	C	1.023337	-0.147292	1.078647	C	1.027993	-0.149586	1.088848
C	2.162892	-0.187949	0.070723	C	1.830307	-0.148808	-0.004633	C	1.834529	-0.144198	0.004799
C	2.003261	1.060140	0.923513	C	1.558478	0.662134	-1.260222	C	1.540783	0.648764	-1.256820
C	1.208290	2.194533	0.245478	C	0.948037	2.053296	-0.993259	C	0.931494	2.041122	-0.995593
C	-0.330396	2.080594	0.311871	C	-0.577786	2.092974	-0.761177	C	-0.591578	2.082353	-0.743762
C	-0.829090	0.601436	0.251077	C	-1.112665	0.818509	-0.034408	C	-1.121799	0.806018	-0.015899
C	-0.179635	-0.162073	-0.907553	C	-0.303465	0.499357	1.223075	C	-0.308290	0.482176	1.235634
C	-2.361523	0.494355	0.225332	C	-2.622756	0.898039	0.246420	C	-2.630697	0.882948	0.272735
C	-2.872548	-0.923782	0.566020	C	-3.268264	-0.438708	0.627618	C	-3.274431	-0.458991	0.638286

C	-4.393417	-0.971803	0.504873	C	-3.340457	-1.474495	-0.485765	C	-3.356067	-1.475174	-0.492797
C	-5.019984	-1.401172	-0.806987	C	-3.857692	-2.848731	-0.098447	C	-3.852533	-2.861593	-0.122612
O	-5.072670	-0.659203	1.470118	O	-3.005870	-1.225314	-1.633781	O	-3.044000	-1.200762	-1.641209
O	-0.806961	-0.417427	-1.933218	O	-0.757787	0.697219	2.348953	O	-0.760348	0.661557	2.365703
C	3.533464	-0.872664	0.127142	C	3.147219	-0.945945	0.027864	C	3.173088	-0.907711	0.001018
C	3.903253	-1.216677	1.585306	C	3.270711	-1.839655	-1.224230	C	3.530172	-1.582214	1.331824
C	4.614114	0.014115	-0.527021	C	4.352602	0.000685	0.155157	C	3.169233	-1.954039	-1.125226
O	3.431450	-2.095665	-0.617103	O	3.231977	-1.779538	1.190905	O	4.232820	0.012855	-0.351615
C	-0.963897	2.986033	-0.756036	C	-0.961980	3.407599	-0.063997	C	-0.968323	3.394424	-0.037967
H	1.439091	-1.505222	-1.397737	H	1.358617	-0.643857	1.984813	H	1.338680	-0.647953	2.001784
H	1.541930	0.799632	1.888046	H	2.500747	0.798975	-1.798973	H	2.483616	0.774342	-1.794631
H	2.995397	1.442610	1.179667	H	0.915957	0.090736	-1.946711	H	0.884121	0.068965	-1.922194
H	1.523761	2.251101	-0.804295	H	1.177168	2.708571	-1.842452	H	1.149517	2.688567	-1.853530
H	1.493956	3.149967	0.702041	H	1.461084	2.488243	-0.125709	H	1.454790	2.487326	-0.139479
H	-0.647771	2.447398	1.298796	H	-1.063825	2.076037	-1.746961	H	-1.089815	2.068067	-1.723302
H	-0.474470	0.123182	1.174015	H	-0.946758	-0.009328	-0.732593	H	-0.957224	-0.019952	-0.716863
H	-2.781927	1.198355	0.953282	H	-2.8040590	1.6041480	1.0608590	H	-2.808671	1.579302	1.096260
H	-2.724471	0.784919	-0.765031	H	-3.119536	1.285441	-0.650241	H	-3.130691	1.280986	-0.617244
H	-2.444118	-1.646379	-0.136470	H	-2.768526	-0.886434	1.496977	H	-2.769397	-0.921652	1.496608
H	-2.569262	-1.191285	1.584148	H	-4.303485	-0.270058	0.960498	H	-4.307465	-0.295036	0.980425
H	-4.848859	-2.475856	-0.950765	H	-4.031818	-3.448571	-0.993340	H	-4.054954	-3.439766	-1.025798

H	-6.093674	-1.206195	-0.797184	H	-3.117444	-3.353079	0.535212	H	-3.085636	-3.376868	0.469315
H	-4.545200	-0.893094	-1.653757	H	-4.779269	-2.77297	0.489162	H	-4.752066	-2.807608	0.500387
H	3.993040	-0.326557	2.214560	H	3.303306	-1.261604	-2.151515	H	2.803332	-2.351708	1.608192
H	3.148322	-1.880071	2.016635	H	4.191281	-2.425766	-1.151233	H	4.509814	-2.05668	1.229270
H	4.868944	-1.734962	1.607555	H	2.423373	-2.532644	-1.290420	H	3.587298	-0.857353	2.151433
H	4.723740	0.978400	-0.022771	H	4.403010	0.70719	-0.677263	H	2.391794	-2.703771	-0.950062
H	5.587503	-0.489623	-0.480012	H	4.286253	0.562054	1.090956	H	2.994676	-1.489642	-2.098426
H	4.365433	0.192890	-1.576723	H	5.275018	-0.58732	0.167879	H	4.141938	-2.453689	-1.158566
H	4.311140	-2.490251	-0.677566	H	2.539892	-2.454167	1.143316	H	4.340744	0.647988	0.370373
H	-0.538183	3.994196	-0.696001	H	-0.597172	3.430795	0.969345	H	-0.589743	3.415523	0.990616
H	-0.774360	2.601682	-1.764696	H	-2.044266	3.561864	-0.042000	H	-2.050348	3.548164	-0.000923
H	-2.045854	3.079137	-0.628647	H	-0.519863	4.259055	-0.594083	H	-0.533514	4.247452	-0.571495
**1b3**				**1b4**				**1b5**			
C	1.025667	-0.161352	1.074910	C	-1.222844	0.604119	0.817861	C	1.020374	-0.153679	1.082781
C	1.833222	-0.156484	-0.006703	C	-2.163643	0.208703	-0.067009	C	1.823591	-0.150371	-0.001829
C	1.525886	0.618983	-1.276854	C	-1.988169	-0.970977	-1.008415	C	1.542726	0.649962	-1.262661
C	0.932314	2.022118	-1.029401	C	-1.249028	-2.171586	-0.384145	C	0.934572	2.043758	-1.003003
C	-0.588260	2.075424	-0.760881	C	0.293324	-2.093430	-0.370597	C	-0.590292	2.083463	-0.762622
C	-1.120930	0.808164	-0.019976	C	0.828514	-0.635522	-0.194724	C	-1.119160	0.811685	-0.027600
C	-0.299161	0.488213	1.228601	C	0.156519	0.076557	0.979930	C	-0.302340	0.501066	1.227937
C	-2.626883	0.896402	0.280214	C	2.361945	-0.577683	-0.116861	C	-2.627717	0.887818	0.261743
C	-3.272605	-0.443408	0.649821	C	2.927730	0.835429	-0.275662	C	-3.262554	-0.454687	0.640046

C	-3.367670	-1.456355	-0.483269	C	4.447463	0.889062	-0.369400	C	-3.342552	-1.479645	-0.482973
C	-3.842542	-2.848692	-0.108629	C	5.083306	2.262323	-0.247119	C	-3.799679	-2.874284	-0.094228
O	-3.080840	-1.174354	-1.636587	O	5.124674	-0.112221	-0.531868	O	-3.058572	-1.206204	-1.639056
O	-0.739310	0.690893	2.359290	O	0.753773	0.284742	2.033832	O	-0.748740	0.715974	2.353859
C	3.163074	-0.933663	0.022476	C	-3.540508	0.897877	-0.134229	C	3.141002	-0.934069	0.022038
C	3.165310	-2.002547	-1.083689	C	-3.764164	1.987918	0.921673	C	3.226189	-1.882240	-1.192376
C	4.371851	0.015157	-0.119786	C	-3.758341	1.485516	-1.538146	C	4.347275	0.028166	0.063194
O	3.316840	-1.679927	1.236265	O	-4.570730	-0.107844	0.010462	O	3.147363	-1.727321	1.219090
C	-0.945922	3.394558	-0.058383	C	0.856713	-3.082326	0.661859	C	-0.971968	3.400342	-0.068481
H	1.333119	-0.695248	1.968651	H	-1.441836	1.384719	1.539520	H	1.346175	-0.676580	1.975571
H	2.443284	0.727593	-1.860957	H	-1.474252	-0.648219	-1.926384	H	2.477413	0.781116	-1.815622
H	0.849061	0.031220	-1.914044	H	-2.984255	-1.295749	-1.318447	H	0.890867	0.073232	-1.935660
H	1.143584	2.655688	-1.899375	H	-1.615225	-2.301465	0.642758	H	1.158183	2.692916	-1.858433
H	1.468640	2.475202	-0.185535	H	-1.535358	-3.082317	-0.923877	H	1.451655	2.485352	-0.141218
H	-1.096666	2.060063	-1.735112	H	0.648859	-2.403956	-1.363849	H	-1.081722	2.062184	-1.745655
H	-0.968938	-0.024606	-0.715693	H	0.513962	-0.091907	-1.096930	H	-0.954660	-0.019384	-0.722387
H	-2.793266	1.593167	1.105794	H	2.7825300	-1.2174640	-0.9002910	H	-2.805767	1.589739	1.080497
H	-3.132375	1.297058	-0.605461	H	2.701641	-0.984977	0.838678	H	-3.131708	1.276163	-0.630364
H	-2.761876	-0.909549	1.502785	H	2.608797	1.482408	0.551136	H	-2.751587	-0.907405	1.500033
H	-4.302439	-0.276674	1.000305	H	2.542812	1.313068	-1.191456	H	-4.295849	-0.294869	0.983755
H	-4.081880	-3.417351	-1.008862	H	4.539160	3.008987	-0.836055	H	-2.996925	-3.37566	0.461098

H	-3.045988	-3.366664	0.440368	H	6.127466	2.221133	-0.561956	H	-4.670851	-2.83751	0.568900
H	-4.712595	-2.80759	0.555672	H	5.039218	2.585906	0.800496	H	-4.030752	-3.455475	-0.988624
H	3.050872	-1.557899	-2.075628	H	-3.066532	2.822084	0.802540	H	3.219107	-1.343391	-2.143944
H	4.110800	-2.551707	-1.053695	H	-3.656049	1.59558	1.938893	H	4.158063	-2.45788	-1.145210
H	2.348692	-2.711143	-0.919320	H	-4.781323	2.373745	0.812868	H	2.387843	-2.584335	-1.178363
H	4.376206	0.556523	-1.069292	H	-3.017779	2.263271	-1.747659	H	4.382706	0.688729	-0.807586
H	4.378254	0.756216	0.688477	H	-4.758506	1.924974	-1.595503	H	4.304750	0.641536	0.967485
H	5.292420	-0.572884	-0.059007	H	-3.684802	0.713776	-2.307828	H	5.282188	-0.545899	0.074369
H	3.500602	-1.068767	1.963141	H	-4.542971	-0.448111	0.915875	H	4.013656	-2.146842	1.300882
H	-0.554819	3.417965	0.965337	H	0.410767	-4.072635	0.514803	H	-0.600069	3.428262	0.962195
H	-2.026329	3.555632	-0.009050	H	0.628139	-2.761899	1.684897	H	-2.054474	3.552107	-0.039356
H	-0.511978	4.241752	-0.601950	H	1.940889	-3.194243	0.578258	H	-0.535449	4.25059	-0.605295
**1b6**				**1b7**							
C	1.099425	0.004280	1.100929	C	-1.205440	-0.666021	-0.751315				
C	1.849425	-0.089037	-0.017827	C	-2.162892	-0.187949	0.070723				
C	1.483625	0.569842	-1.337110	C	-2.003261	1.060140	0.923513				
C	0.801808	1.946143	-1.195768	C	-1.208290	2.194533	0.245478				
C	-0.713722	1.928100	-0.899388	C	0.330396	2.080594	0.311871				
C	-1.131888	0.719091	-0.006637	C	0.829090	0.601436	0.251077				
C	-0.246871	0.605866	1.238511	C	0.179635	-0.162073	-0.907553				
C	-2.624453	0.738652	0.358820	C	2.361523	0.494355	0.225332				
C	-3.165976	-0.617134	0.884121	C	2.872548	-0.923782	0.566020				
C	-3.210431	-1.673863	-0.208582	C	4.393417	-0.971803	0.504873				

C	-4.538623	-1.895963	-0.906825	C	5.019984	-1.401172	-0.806987
O	-2.211880	-2.303455	-0.527806	O	5.072670	-0.659203	1.470118
O	-0.657339	0.935166	2.349542	O	0.806961	-0.417427	-1.933218
C	3.190734	-0.843203	0.024756	C	-3.533464	-0.872664	0.127142
C	3.279124	-1.854165	-1.138200	C	-3.903253	-1.216677	1.585306
C	4.371998	0.140742	-0.004522	C	-4.614114	0.014115	-0.527021
O	3.355464	-1.553972	1.258073	O	-3.431450	-2.095665	-0.617103
C	-1.150788	3.290974	-0.339738	C	0.963897	2.986033	-0.756036
H	1.496841	-0.379915	2.035897	H	-1.439091	-1.505222	-1.397737
H	2.396926	0.700443	-1.925351	H	-2.995397	1.442610	1.179667
H	0.854111	-0.108209	-1.932718	H	-1.541930	0.799632	1.888046
H	0.958073	2.515278	-2.120317	H	-1.493956	3.149967	0.702041
H	1.320092	2.502514	-0.404072	H	-1.523761	2.251101	-0.804295
H	-1.235884	1.771244	-1.855054	H	0.647771	2.447398	1.298796
H	-0.937670	-0.181722	-0.599134	H	0.474470	0.123182	1.174015
H	-2.797969	1.486229	1.137187	H	2.7244710	0.7849190	-0.7650310
H	-3.204711	1.045702	-0.521923	H	2.781927	1.198355	0.953282
H	-2.524306	-0.974708	1.694729	H	2.569262	-1.191285	1.584148
H	-4.171061	-0.45972	1.291094	H	2.444118	-1.646379	-0.136470
H	-5.247688	-2.348342	-0.201874	H	6.093674	-1.206195	-0.797184
H	-4.976157	-0.941965	-1.224104	H	4.848859	-2.475856	-0.950765

H	-4.411780	-2.556396	-1.766186	H	4.545200	-0.893094	-1.653757
H	3.256790	-1.369633	-2.118045	H	-3.993040	-0.326557	2.214560
H	4.216751	-2.410556	-1.050791	H	-4.868944	-1.734962	1.607555
H	2.447213	-2.566995	-1.096323	H	-3.148322	-1.880071	2.016635
H	4.359707	0.763496	-0.903098	H	-4.723740	0.9784	-0.022771
H	4.336213	0.78985	0.874546	H	-4.365433	0.19289	-1.576723
H	5.311134	-0.420027	0.015778	H	-5.587503	-0.489623	-0.480012
H	2.673874	-2.238731	1.317007	H	-4.311140	-2.490251	-0.677566
H	-0.736634	3.460018	0.660712	H	2.045854	3.079137	-0.628647
H	-2.238842	3.379893	-0.271542	H	0.538183	3.994196	-0.696001
H	-0.795299	4.098402	-0.990125	H	0.774360	2.601682	-1.764696

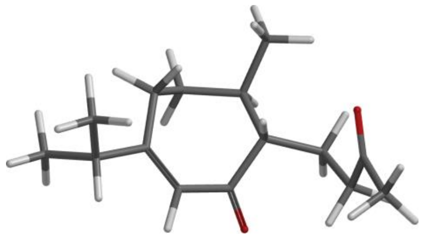
2a1 (41.40%)

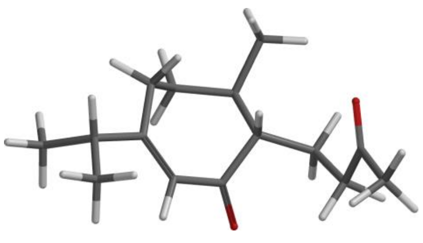
2a2 (25.69%)

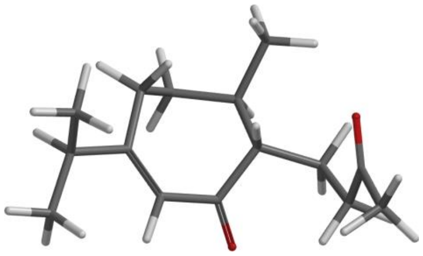
2a3 (14.06%)

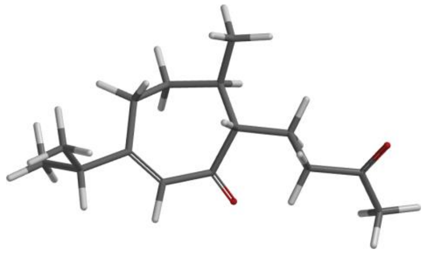
2a4 (12.85%)

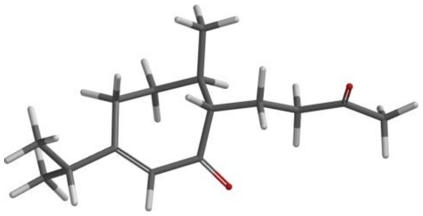
2a5 (3.16%)

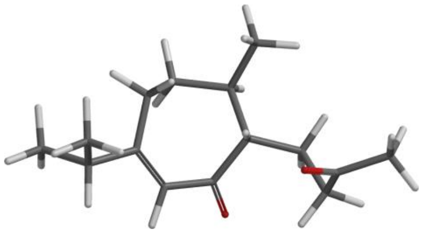
2a6 (2.84%)

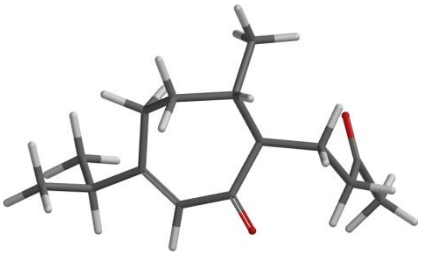

2b1 (41.40%)

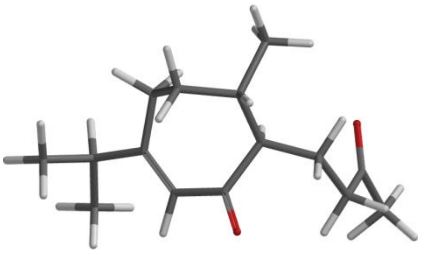

2b2 (25.70%)

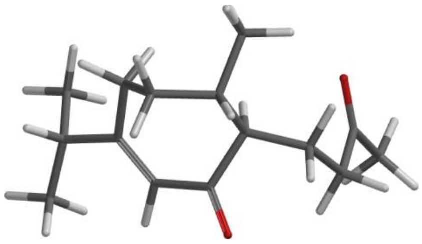

2b3 (14.06%)

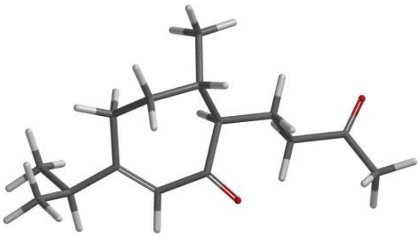

2b4 (12.85%)

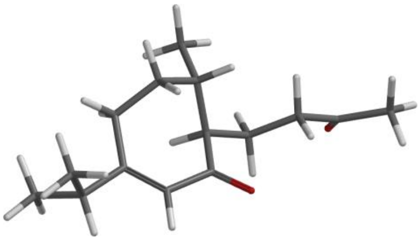

2b5 (3.15%)

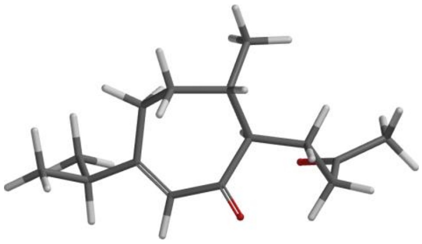

2b6 (2.84%)

Figure S85. Stable conformers of compound **2** with 1*R*,10*S* (**2a**) and 1*S*,10*R* (**2b**) configurations, respectively

Table S3. Important thermodynamic parameters (a.u.) of the optimized compound **2** at B3LYP/6-31G (d,p) level in the gas phase

conformations	E+ZPE	G	conformations	E+ZPE	G
**2a1**	-736.192276	-736.240804	**2b1**	-736.192276	-736.240804
**2a2**	-736.191726	-736.240354	**2b2**	-736.191726	-736.240354
**2a3**	-736.191506	-736.239785	**2b3**	-736.191506	-736.239785
**2a4**	-736.190763	-736.239700	**2b4**	-736.190763	-736.239700
**2a5**	-736.189789	-736.238376	**2b5**	-736.189791	-736.238375
**2a6**	-736.189545	-736.238278	**2b6**	-736.189545	-736.238278

Table S4. Optimized Z-Matrixes of compound **1** in the Gas Phase (Å) at B3LYP/6-31G (d,p) level

**2a1**				**2a2**				**2a3**			
C	1.163702	-0.732866	1.069014	C	1.275768	-0.743947	0.726770	C	1.314614	-0.561057	0.983039
C	2.076012	-0.424215	0.123020	C	2.093597	-0.245676	-0.223709	C	2.170754	-0.093077	0.050128
C	2.003877	0.830355	-0.724647	C	1.902142	1.120481	-0.852488	C	1.946441	1.210424	-0.695441
C	1.385856	2.052656	-0.012149	C	1.302444	2.191319	0.082844	C	1.243635	2.308615	0.131044
C	-0.156365	2.119738	-0.036704	C	-0.237946	2.183322	0.188338	C	-0.298585	2.234016	0.139543
C	-0.837777	0.757269	0.310478	C	-0.831731	0.748790	0.358533	C	-0.836688	0.784113	0.359012
C	-0.083986	-0.006284	1.408849	C	0.036317	-0.134196	1.267273	C	0.004095	0.009047	1.383266
C	-2.317864	0.946310	0.695009	C	-2.284526	0.795559	0.870186	C	-2.324302	0.784379	0.760672
C	-3.136415	-0.348006	0.752568	C	-3.038345	-0.536936	0.794735	C	-3.002094	-0.589871	0.723520
C	-3.407660	-1.005392	-0.593265	C	-3.379480	-1.013342	-0.610199	C	-3.204896	-1.179340	-0.665127
C	-4.033603	-2.388350	-0.556770	C	-3.956124	-2.413165	-0.728415	C	-3.697354	-2.614618	-0.725235
O	-3.142920	-0.458773	-1.652786	O	-3.206369	-0.315363	-1.597303	O	-2.988996	-0.541805	-1.684174
O	-0.541710	-0.105850	2.546002	O	-0.322753	-0.421705	2.407910	O	-0.425968	-0.226566	2.511311

C	3.285311	-1.331409	-0.070104	C	3.341985	-0.993595	-0.680474	C	3.475164	-0.808744	-0.282492
C	3.426640	-1.792336	-1.534863	C	4.598732	-0.441798	0.031573	C	3.841055	-1.977222	0.641345
C	4.590196	-0.679692	0.433233	C	3.270821	-2.520421	-0.534150	C	3.463898	-1.275579	-1.757577
C	-0.647140	2.657825	-1.392903	C	-0.858483	2.903579	-1.022192	C	-0.870250	2.853026	-1.148736
H	1.341972	-1.588695	1.718412	H	1.526784	-1.679472	1.219877	H	1.580032	-1.430075	1.577837
H	3.017694	1.086472	-1.049548	H	2.877470	1.466307	-1.213727	H	2.924484	1.582191	-1.023521
H	1.454168	0.615611	-1.652780	H	1.286551	1.015806	-1.759116	H	1.385814	1.021350	-1.622350
H	1.770389	2.966554	-0.480741	H	1.612477	3.182939	-0.268327	H	1.532594	3.289305	-0.265443
H	1.744536	2.064794	1.023855	H	1.746562	2.065317	1.077440	H	1.627415	2.267151	1.157300
H	-0.458830	2.833725	0.742445	H	-0.503042	2.747304	1.093808	H	-0.647253	2.835867	0.990679
H	-0.781245	0.138463	-0.595116	H	-0.819621	0.283111	-0.635553	H	-0.728753	0.259569	-0.599353
H	-2.790914	1.618901	-0.024893	H	-2.842405	1.537751	0.293508	H	-2.873401	1.455231	0.095185
H	-2.360765	1.425936	1.677724	H	-2.272496	1.124861	1.913850	H	-2.408494	1.181148	1.777213
H	-4.122770	-0.143720	1.196603	H	-3.996280	-0.451268	1.330085	H	-4.003355	-0.521189	1.175551
H	-2.677038	-1.082695	1.425799	H	-2.496955	-1.332501	1.322014	H	-2.466171	-1.316433	1.347085
H	-4.396203	-2.661715	-1.549225	H	-3.166638	-3.148857	-0.529678	H	-4.013770	-2.859438	-1.740651
H	-4.850092	-2.438017	0.171866	H	-4.347928	-2.572668	-1.734453	H	-4.519869	-2.783463	-0.021576
H	-3.277800	-3.117933	-0.239334	H	-4.743249	-2.582698	0.014638	H	-2.883073	-3.289089	-0.431664
H	3.112893	-2.225460	0.542003	H	3.453042	-0.770571	-1.752757	H	4.271615	-0.053456	-0.191327
H	4.254717	-2.503413	-1.627706	H	5.503734	-0.903151	-0.378635	H	4.821320	-2.376647	0.361159
H	2.515171	-2.286819	-1.886963	H	4.696287	0.642530	-0.082677	H	3.892890	-1.667089	1.689755
H	3.637630	-0.952533	-2.206053	H	4.557318	-0.664907	1.103301	H	3.117880	-2.796941	0.563287
H	5.422610	-1.386010	0.340414	H	4.148092	-2.980747	-1.001155	H	4.429410	-1.719867	-2.021922
H	4.507295	-0.389398	1.485450	H	2.375725	-2.929062	-1.013810	H	3.276110	-0.450893	-2.451473
H	4.852629	0.212825	-0.145785	H	3.264021	-2.830040	0.516769	H	2.687431	-2.033030	-1.914177
H	-0.322203	2.014067	-2.218716	H	-1.950292	2.917876	-0.984974	H	-0.514901	2.320471	-2.038685
H	-1.736546	2.721016	-1.446754	H	-0.512094	3.942339	-1.064504	H	-1.962199	2.827657	-1.174844
H	-0.244951	3.661417	-1.571287	H	-0.575127	2.416500	-1.962533	H	-0.558951	3.899969	-1.236935
**2a4**				**2a5**				**2a6**			
C	-1.327293	-1.207407	-0.341922	C	-1.574832	-1.307231	0.029608	C	1.278523	-0.548077	1.178829
C	-2.374709	-0.464902	0.075694	C	-2.550259	-0.374546	0.084658	C	2.119151	-0.327691	0.146322
C	-2.354600	1.050571	0.124148	C	-2.293678	1.110374	-0.088718	C	1.909575	0.772797	-0.875361
C	-1.502112	1.718329	-0.976818	C	-1.184202	1.445989	-1.108937	C	1.248600	2.050923	-0.313270
C	0.006289	1.832366	-0.665869	C	0.261819	1.403773	-0.562997	C	-0.294526	2.020230	-0.252404
C	0.609792	0.518410	-0.075068	C	0.508616	0.207279	0.405402	C	-0.852839	0.674448	0.306649
C	0.020733	-0.736983	-0.739569	C	-0.112545	-1.099961	-0.104649	C	-0.004176	0.133732	1.469944
C	2.146582	0.503157	-0.159615	C	1.983733	0.015198	0.828240	C	-2.325074	0.797387	0.740767
C	2.802022	-0.574228	0.733644	C	3.005931	-0.140428	-0.301355	C	-3.054479	-0.557041	0.938594
C	4.315590	-0.550550	0.566761	C	4.435704	-0.304373	0.193983	C	-3.294274	-1.272317	-0.382032
C	4.911651	-1.483757	-0.468451	C	5.461756	-0.731754	-0.840741	C	-4.671068	-1.138896	-1.005647
O	5.010603	0.204453	1.228403	O	4.751093	-0.111214	1.357016	O	-2.408868	-1.912833	-0.929914

O	0.680613	-1.402487	-1.534380	O	0.583106	-2.018690	-0.533582	O	-0.410776	0.181378	2.629047
C	-3.678483	-1.159881	0.449705	C	-3.997009	-0.819500	0.259474	C	3.380692	-1.172138	0.017049
C	-4.148398	-0.773151	1.866433	C	-4.662022	-0.137478	1.472230	C	3.465370	-1.860570	-1.360365
C	-4.791195	-0.909107	-0.589857	C	-4.831135	-0.607988	-1.021456	C	4.660590	-0.362180	0.311467
C	0.274159	3.033510	0.258825	C	0.631216	2.738242	0.108746	C	-0.886193	2.336676	-1.638235
H	-1.452412	-2.282299	-0.462270	H	-1.846383	-2.361298	0.054335	H	1.547360	-1.282021	1.936560
H	-3.384709	1.411715	0.039763	H	-3.224483	1.589089	-0.409153	H	2.881795	1.037750	-1.303509
H	-2.019326	1.382827	1.117790	H	-2.062588	1.563567	0.886981	H	1.323563	0.381545	-1.720112
H	-1.886752	2.729059	-1.158215	H	-1.364774	2.449791	-1.511920	H	1.546132	2.906445	-0.931666
H	-1.649610	1.162353	-1.910026	H	-1.281525	0.755544	-1.954853	H	1.657457	2.238510	0.686374
H	0.519679	2.017205	-1.619642	H	0.919268	1.272331	-1.431388	H	-0.607132	2.814084	0.440692
H	0.309760	0.477392	0.982774	H	-0.045149	0.444991	1.325797	H	-0.773840	-0.068489	-0.497451
H	2.543302	1.477202	0.139800	H	2.041070	-0.873077	1.465963	H	-2.878147	1.397147	0.009553
H	2.430357	0.339163	-1.204738	H	2.283741	0.851683	1.466046	H	-2.360543	1.336699	1.692103
H	2.409713	-1.561595	0.469620	H	3.014594	0.736143	-0.967043	H	-4.010491	-0.370183	1.440350
H	2.576959	-0.367046	1.785504	H	2.750833	-0.994824	-0.937850	H	-2.454613	-1.203691	1.585252
H	5.959430	-1.235355	-0.645609	H	5.335245	-0.183310	-1.780656	H	-5.404710	-1.664236	-0.380989
H	4.343652	-1.445364	-1.404570	H	5.317417	-1.795337	-1.068883	H	-4.677419	-1.566747	-2.009471
H	4.842063	-2.516103	-0.101977	H	6.470709	-0.586141	-0.450796	H	-4.983152	-0.088250	-1.040463
H	-3.474621	-2.237824	0.452411	H	-3.976241	-1.898021	0.458495	H	3.313312	-1.960899	0.776475
H	-5.042061	-1.346422	2.135634	H	-5.672598	-0.533680	1.619303	H	4.335998	-2.524487	-1.396171
H	-3.375849	-0.981816	2.614043	H	-4.092858	-0.315052	2.390770	H	2.572255	-2.461469	-1.560216
H	-4.407824	0.289296	1.932902	H	-4.751308	0.945551	1.331860	H	3.573928	-1.132434	-2.172164
H	-5.687609	-1.480312	-0.324641	H	-5.845038	-0.997281	-0.877748	H	5.536656	-1.019249	0.277851
H	-4.475212	-1.219050	-1.591006	H	-4.385291	-1.129550	-1.874401	H	4.618974	0.097538	1.304127
H	-5.078591	0.147160	-0.635919	H	-4.921868	0.452631	-1.281286	H	4.820552	0.433227	-0.425026
H	-0.262080	2.931733	1.210192	H	1.669553	2.754890	0.451378	H	-1.979894	2.339640	-1.635734
H	1.335501	3.152233	0.492336	H	0.501437	3.569194	-0.593353	H	-0.557607	3.325202	-1.977835
H	-0.063643	3.961858	-0.214590	H	-0.005905	2.935822	0.979991	H	-0.562511	1.602305	-2.385453
**2b1**				**2b2**				**2b3**			
C	-1.163702	-0.732866	1.069014	C	-1.275768	-0.743947	0.726770	C	-1.314614	-0.561057	0.983039
C	-2.076012	-0.424215	0.123020	C	-2.093597	-0.245676	-0.223709	C	-2.170754	-0.093077	0.050128
C	-2.003877	0.830355	-0.724647	C	-1.902142	1.120481	-0.852488	C	-1.946441	1.210424	-0.695441
C	-1.385856	2.052656	-0.012149	C	-1.302444	2.191319	0.082844	C	-1.243635	2.308615	0.131044
C	0.156365	2.119738	-0.036704	C	0.237946	2.183322	0.188338	C	0.298585	2.234016	0.139543
C	0.837777	0.757269	0.310478	C	0.831731	0.748790	0.358533	C	0.836688	0.784113	0.359012
C	0.083986	-0.006284	1.408849	C	-0.036317	-0.134196	1.267273	C	-0.004095	0.009047	1.383266
C	2.317864	0.946310	0.695009	C	2.284526	0.795559	0.870186	C	2.324302	0.784379	0.760672
C	3.136415	-0.348006	0.752568	C	3.038345	-0.536936	0.794735	C	3.002094	-0.589871	0.723520
C	3.407660	-1.005392	-0.593265	C	3.379480	-1.013342	-0.610199	C	3.204896	-1.179340	-0.665127
C	4.033603	-2.388350	-0.556770	C	3.956124	-2.413165	-0.728415	C	3.697354	-2.614618	-0.725235

O	3.142920	-0.458773	-1.652786	O	3.206369	-0.315363	-1.597303	O	2.988996	-0.541805	-1.684174
O	0.541710	-0.105850	2.546002	O	0.322753	-0.421705	2.407910	O	0.425968	-0.226566	2.511311
C	-3.285311	-1.331409	-0.070104	C	-3.341985	-0.993595	-0.680474	C	-3.475164	-0.808744	-0.282492
C	-3.426640	-1.792336	-1.534863	C	-4.598732	-0.441798	0.031573	C	-3.841055	-1.977222	0.641345
C	-4.590196	-0.679692	0.433233	C	-3.270821	-2.520421	-0.534150	C	-3.463898	-1.275579	-1.757577
C	0.647140	2.657825	-1.392903	C	0.858483	2.903579	-1.022192	C	0.870250	2.853026	-1.148736
H	-1.341972	-1.588695	1.718412	H	-1.526784	-1.679472	1.219877	H	-1.580032	-1.430075	1.577837
H	-1.454168	0.615611	-1.652780	H	-1.286551	1.015806	-1.759116	H	-1.385814	1.021350	-1.622350
H	-3.017694	1.086472	-1.049548	H	-2.877470	1.466307	-1.213727	H	-2.924484	1.582191	-1.023521
H	-1.770389	2.966554	-0.480741	H	-1.612477	3.182939	-0.268327	H	-1.532594	3.289305	-0.265443
H	-1.744536	2.064794	1.023855	H	-1.746562	2.065317	1.077440	H	-1.627415	2.267151	1.157300
H	0.458830	2.833725	0.742445	H	0.503042	2.747304	1.093808	H	0.647253	2.835867	0.990679
H	0.781245	0.138463	-0.595116	H	0.819621	0.283111	-0.635553	H	0.728753	0.259569	-0.599353
H	2.790914	1.618901	-0.024893	H	2.842405	1.537751	0.293508	H	2.873401	1.455231	0.095185
H	2.360765	1.425936	1.677724	H	2.272496	1.124861	1.913850	H	2.408494	1.181148	1.777213
H	4.122770	-0.143720	1.196603	H	3.996280	-0.451268	1.330085	H	4.003355	-0.521189	1.175551
H	2.677038	-1.082695	1.425799	H	2.496955	-1.332501	1.322014	H	2.466171	-1.316433	1.347085
H	3.277800	-3.117933	-0.239334	H	3.166638	-3.148857	-0.529678	H	4.519869	-2.783463	-0.021576
H	4.850092	-2.438017	0.171866	H	4.743249	-2.582698	0.014638	H	4.013770	-2.859438	-1.740651
H	4.396203	-2.661715	-1.549225	H	4.347928	-2.572668	-1.734453	H	2.883073	-3.289089	-0.431664
H	-3.112893	-2.225460	0.542003	H	-3.453042	-0.770571	-1.752757	H	-4.271615	-0.053456	-0.191327
H	-4.254717	-2.503413	-1.627706	H	-5.503734	-0.903151	-0.378635	H	-4.821320	-2.376647	0.361159
H	-2.515171	-2.286819	-1.886963	H	-4.696287	0.642530	-0.082677	H	-3.892890	-1.667089	1.689755
H	-3.637630	-0.952533	-2.206053	H	-4.557318	-0.664907	1.103301	H	-3.117880	-2.796941	0.563287
H	-5.422610	-1.386010	0.340414	H	-4.148092	-2.980747	-1.001155	H	-4.429410	-1.719867	-2.021922
H	-4.507295	-0.389398	1.485450	H	-2.375725	-2.929062	-1.013810	H	-3.276110	-0.450893	-2.451473
H	-4.852629	0.212825	-0.145785	H	-3.264021	-2.830040	0.516769	H	-2.687431	-2.033030	-1.914177
H	1.736546	2.721016	-1.446754	H	1.950292	2.917876	-0.984974	H	1.962199	2.827657	-1.174844
H	0.244951	3.661417	-1.571287	H	0.512094	3.942339	-1.064504	H	0.558951	3.899969	-1.236935
H	0.322203	2.014067	-2.218716	H	0.575127	2.416500	-1.962533	H	0.514901	2.320471	-2.038685
**2b4**				**2b5**				**2b6**			
C	1.327293	-1.207407	-0.341922	C	1.574954	-1.307219	0.029692	C	-1.278523	-0.548077	1.178829
C	2.374709	-0.464902	0.075694	C	2.550332	-0.374462	0.084488	C	-2.119151	-0.327691	0.146322
C	2.354600	1.050571	0.124148	C	2.293627	1.110376	-0.089357	C	-1.909575	0.772797	-0.875361
C	1.502112	1.718329	-0.976818	C	1.183939	1.445590	-1.109472	C	-1.248600	2.050923	-0.313270
C	-0.006289	1.832366	-0.665869	C	-0.261938	1.403544	-0.563191	C	0.294526	2.020230	-0.252404
C	-0.609792	0.518410	-0.075068	C	-0.508607	0.207099	0.405350	C	0.852839	0.674448	0.306649
C	-0.020733	-0.736983	-0.739569	C	0.112637	-1.100076	-0.104676	C	0.004176	0.133732	1.469944
C	-2.146582	0.503157	-0.159615	C	-1.983676	0.014969	0.828192	C	2.325074	0.797387	0.740767
C	-2.802022	-0.574228	0.733644	C	-3.005954	-0.140799	-0.301313	C	3.054479	-0.557041	0.938594
C	-4.315590	-0.550550	0.566761	C	-4.435754	-0.304195	0.194119	C	3.294274	-1.272317	-0.382032

C	-4.911651	-1.483757	-0.468451	C	-5.461938	-0.731673	-0.840420	C	4.671068	-1.138896	-1.005647
O	-5.010603	0.204453	1.228403	O	-4.751081	-0.110479	1.357093	O	2.408868	-1.912833	-0.929914
O	-0.680613	-1.402487	-1.534380	O	-0.582922	-2.018844	-0.533680	O	0.410776	0.181378	2.629047
C	3.678483	-1.159881	0.449705	C	3.997061	-0.819240	0.259821	C	-3.380692	-1.172138	0.017049
C	4.148398	-0.773151	1.866433	C	4.661724	-0.136666	1.472487	C	-3.465370	-1.860570	-1.360365
C	4.791195	-0.909107	-0.589857	C	4.831514	-0.608256	-1.020979	C	-4.660590	-0.362180	0.311467
C	-0.274159	3.033510	0.258825	C	-0.631117	2.738048	0.108581	C	0.886193	2.336676	-1.638235
H	1.452412	-2.282299	-0.462270	H	1.846550	-2.361263	0.054858	H	-1.547360	-1.282021	1.936560
H	2.019326	1.382827	1.117790	H	2.062675	1.563861	0.886239	H	-1.323563	0.381545	-1.720112
H	3.384709	1.411715	0.039763	H	3.224363	1.589033	-0.410085	H	-2.881795	1.037750	-1.303509
H	1.649610	1.162353	-1.910026	H	1.281087	0.754839	-1.955164	H	-1.546132	2.906445	-0.931666
H	1.886752	2.729059	-1.158215	H	1.364384	2.449237	-1.512906	H	-1.657457	2.238510	0.686374
H	-0.519679	2.017205	-1.619642	H	-0.919618	1.272059	-1.431399	H	0.607132	2.814084	0.440692
H	-0.309760	0.477392	0.982774	H	0.045166	0.444943	1.325701	H	0.773840	-0.068489	-0.497451
H	-2.543302	1.477202	0.139800	H	-2.040951	-0.873266	1.465999	H	2.878147	1.397147	0.009553
H	-2.430357	0.339163	-1.204738	H	-2.283770	0.851446	1.465980	H	2.360543	1.336699	1.692103
H	-2.409713	-1.561595	0.469620	H	-3.014393	0.735509	-0.967340	H	4.010491	-0.370183	1.440350
H	-2.576959	-0.367046	1.785504	H	-2.751108	-0.995499	-0.937503	H	2.454613	-1.203691	1.585252
H	-4.343652	-1.445364	-1.404570	H	-5.335455	-0.183380	-1.780425	H	4.677419	-1.566747	-2.009471
H	-5.959430	-1.235355	-0.645609	H	-6.470848	-0.585866	-0.450430	H	5.404710	-1.664236	-0.380989
H	-4.842063	-2.516103	-0.101977	H	-5.317755	-1.795315	-1.068363	H	4.983152	-0.088250	-1.040463
H	3.474621	-2.237824	0.452411	H	3.976315	-1.897675	0.459310	H	-3.313312	-1.960899	0.776475
H	5.042061	-1.346422	2.135634	H	5.672134	-0.533031	1.620254	H	-4.335998	-2.524487	-1.396171
H	3.375849	-0.981816	2.614043	H	4.092117	-0.313546	2.390888	H	-2.572255	-2.461469	-1.560216
H	4.407824	0.289296	1.932902	H	4.751353	0.946256	1.331516	H	-3.573928	-1.132434	-2.172164
H	5.687609	-1.480312	-0.324641	H	5.845431	-0.997335	-0.876798	H	-5.536656	-1.019249	0.277851
H	4.475212	-1.219050	-1.591006	H	4.385980	-1.130319	-1.873783	H	-4.618974	0.097538	1.304127
H	5.078591	0.147160	-0.635919	H	4.922155	0.452240	-1.281337	H	-4.820552	0.433227	-0.425026
H	-1.335501	3.152233	0.492336	H	-1.669317	2.754713	0.451631	H	1.979894	2.339640	-1.635734
H	0.063643	3.961858	-0.214590	H	-0.501650	3.568970	-0.593621	H	0.557607	3.325202	-1.977835
H	0.262080	2.931733	1.210192	H	0.006347	2.935736	0.979547	H	0.562511	1.602305	-2.385453

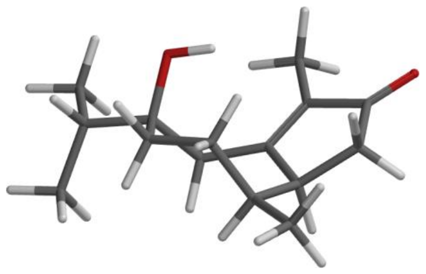
3a1 (39.15%)

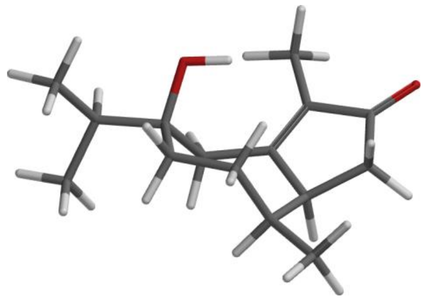
3a2 (23.84%)

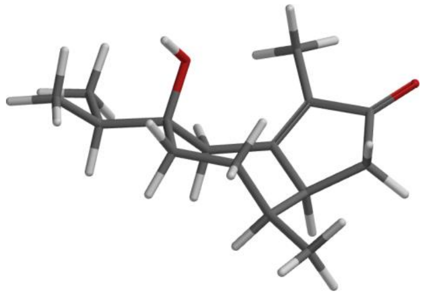
3a3 (11.48%)

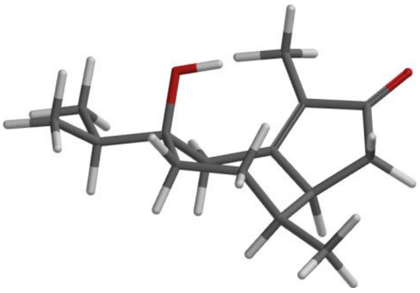

3a4 (11.13%)

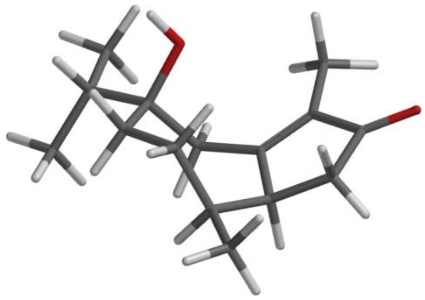

3a5 (10.32%)

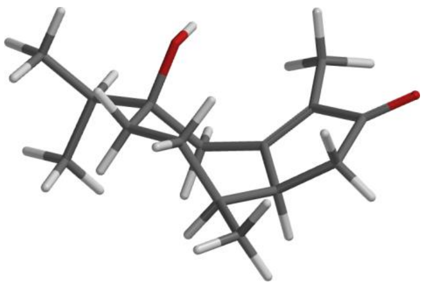

3a6 (4.08%)

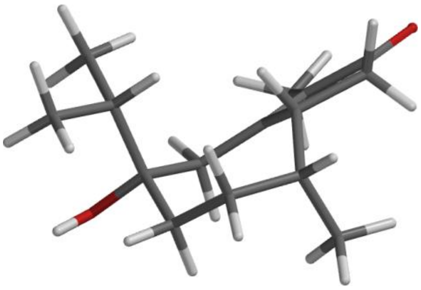

3b1 (47.47%)

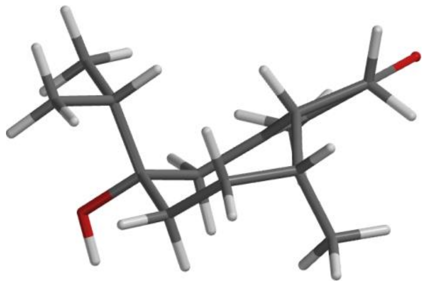

3b2 (32.34%)

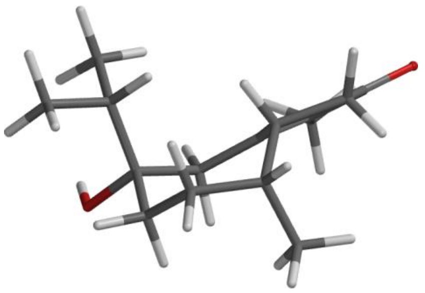

3b3 (20.20%)

Figure S86. Stable conformers of compound **3** with 1*S*,7*R*,10*S* (**3a**) and 1*R*,7*S*,10*R* (**3b**) configurations, respectively

Table S5. Important thermodynamic parameters (a.u.) of the optimized compound **3** at B3LYP/6-31G (d,p) level in the gas phase

conformations	E+ZPE	G	conformations	E+ZPE	G
**3a1**	-736.195975	-736.240730	**3b1**	-736.193420	-736.238410
**3a2**	-736.196083	-736.240262	**3b2**	-736.193089	-736.238048
**3a3**	-736.193588	-736.239573	**3b3**	-736.192844	-736.237604
**3a4**	-736.194878	-736.239544			
**3a5**	-736.194218	-736.239472			
**3a6**	-736.194512	-736.238597			

Table S6. Optimized Z-Matrixes of compound **3** in the Gas Phase (Å) at B3LYP/6-31G (d,p) level

**3a1**				**2a2**				**2a3**			
C	1.619764	0.081127	0.365733	C	-1.590122	-0.238150	-0.169268	C	1.625332	0.087788	0.080015
C	1.311252	1.528333	0.823324	C	-1.521009	1.229484	-0.649536	C	1.323177	1.542385	0.520793
C	-0.161646	1.934037	1.034399	C	-0.136043	1.831946	-0.965696	C	-0.132150	1.939448	0.835776
C	-0.974470	2.088411	-0.271056	C	0.748457	2.106690	0.271599	C	-1.042398	2.063022	-0.406409
C	-1.592052	0.753944	-0.766126	C	1.574544	0.872603	0.722919	C	-1.708462	0.715727	-0.787856
C	-0.675066	-0.457479	-0.629010	C	0.821032	-0.452183	0.661835	C	-0.786966	-0.486820	-0.628232
C	0.797212	-0.347151	-0.896332	C	-0.629119	-0.529234	1.034503	C	0.657922	-0.419028	-1.030722
C	-2.899686	0.289559	-0.089757	C	2.878828	0.575690	-0.047734	C	-2.965940	0.304015	0.007161
C	-2.765170	-1.226533	0.010113	C	2.944392	-0.946702	-0.121615	C	-2.818672	-1.200701	0.211744
C	-1.345972	-1.573890	-0.243887	C	1.606117	-1.476247	0.237696	C	-1.422301	-1.567891	-0.114865
C	-2.038650	3.193530	-0.165540	C	1.645850	3.340330	0.079985	C	-2.077024	3.191147	-0.261766

C	3.154909	-0.074729	0.119060	C	-3.041954	-0.663208	0.215778	C	3.108289	-0.014158	-0.432701
C	3.612762	-1.541582	0.039644	C	-3.594327	0.019689	1.478596	C	4.138984	0.507211	0.589213
C	3.673694	0.698941	-1.106332	C	-4.036449	-0.509040	-0.948582	C	3.506916	-1.439198	-0.859444
C	-0.870826	-2.987815	-0.100711	C	1.316956	-2.943624	0.143218	C	-0.913273	-2.951337	0.142555
O	-3.663203	-2.012078	0.276633	O	3.919622	-1.610032	-0.442730	O	-3.697425	-1.958399	0.600116
O	1.334718	-0.815286	1.451325	O	-1.252195	-1.116762	-1.256435	O	1.413790	-0.808866	1.185413
H	-1.794528	0.883393	-1.841418	H	1.837642	1.043551	1.779197	H	-1.984458	0.788381	-1.852155
H	1.851592	1.662989	1.767738	H	-2.133832	1.290015	-1.554274	H	1.923120	1.740048	1.417643
H	1.747877	2.236197	0.108823	H	-2.001428	1.872835	0.099064	H	1.708211	2.224591	-0.250569
H	-0.154050	2.897503	1.558475	H	-0.315948	2.782610	-1.482657	H	-0.092299	2.912432	1.341452
H	-0.666690	1.238292	1.720554	H	0.403767	1.208907	-1.693935	H	-0.564469	1.238246	1.561535
H	-0.266595	2.411831	-1.047647	H	0.065894	2.344714	1.100282	H	-0.394875	2.342795	-1.250285
H	1.183226	-1.310239	-1.240983	H	-0.869395	-1.529259	1.409988	H	0.983846	-1.418112	-1.324813
H	0.974787	0.381723	-1.694878	H	-0.838324	0.184551	1.838075	H	0.778412	0.233317	-1.905114
H	-3.814970	0.571369	-0.616691	H	3.784415	0.988942	0.403697	H	-3.916022	0.547978	-0.475603
H	-2.982308	0.680691	0.932713	H	2.829456	0.952442	-1.077530	H	-2.979659	0.766785	1.002272
H	-2.621259	3.277309	-1.090269	H	2.285743	3.508739	0.953950	H	-2.733596	3.245290	-1.137822
H	-1.567457	4.164576	0.021062	H	1.037819	4.239502	-0.066295	H	-1.577832	4.160887	-0.157865
H	-2.740582	3.004956	0.654185	H	2.297396	3.237608	-0.794835	H	-2.710529	3.050667	0.620861
H	3.625515	0.358301	1.013005	H	-2.959211	-1.738331	0.426550	H	3.169108	0.636805	-1.317170
H	4.706540	-1.582312	-0.009780	H	-4.593109	-0.372340	1.698163	H	5.149111	0.404777	0.179912
H	3.284999	-2.110840	0.910830	H	-2.976280	-0.157798	2.363854	H	3.998283	1.560963	0.842731
H	3.232710	-2.039970	-0.860217	H	-3.696743	1.102988	1.346916	H	4.117667	-0.075715	1.519425
H	4.767484	0.653209	-1.133469	H	-4.977056	-1.012765	-0.699858	H	4.574021	-1.472056	-1.103106
H	3.394881	1.756855	-1.097348	H	-3.644471	-0.952081	-1.866834	H	2.967104	-1.783690	-1.745344
H	3.311073	0.261244	-2.043594	H	-4.274866	0.542890	-1.143412	H	3.321719	-2.152739	-0.050001
H	-1.447269	-3.642930	-0.764056	H	2.067889	-3.505461	0.709948	H	-1.700313	-3.681549	-0.070170
H	0.191495	-3.096445	-0.326571	H	0.322795	-3.197791	0.515624	H	-0.029892	-3.187885	-0.456189
H	-1.043667	-3.355726	0.916846	H	1.391566	-3.292916	-0.892927	H	-0.635469	-3.069317	1.196128
H	0.378753	-0.835262	1.597892	H	-0.306053	-1.035449	-1.441615	H	1.937291	-0.504440	1.938449
**3a4**				**3a5**				**3a6**			
C	-1.623238	0.101055	-0.067991	C	1.620415	0.076280	0.359355	C	-1.592578	-0.238636	-0.182782
C	-1.329001	1.563162	-0.491155	C	1.327410	1.534062	0.785800	C	-1.529102	1.228088	-0.646859
C	0.123480	1.974878	-0.809358	C	-0.142643	1.929268	1.022814	C	-0.142070	1.822170	-0.962920
C	1.057549	2.070158	0.416562	C	-0.964844	2.087996	-0.276794	C	0.737131	2.101528	0.277249
C	1.714703	0.712005	0.773458	C	-1.590981	0.756499	-0.769389	C	1.571405	0.872795	0.726662
C	0.775974	-0.482287	0.647423	C	-0.689436	-0.463616	-0.615951	C	0.831249	-0.457670	0.648527
C	-0.660302	-0.386716	1.070165	C	0.787576	-0.379813	-0.873364	C	-0.621257	-0.551479	1.005136
C	2.946889	0.286759	-0.053580	C	-2.905520	0.305875	-0.097351	C	2.882093	0.589606	-0.038633
C	2.785122	-1.218303	-0.240578	C	-2.787989	-1.212050	-0.001224	C	2.964140	-0.931621	-0.110172
C	1.392828	-1.574352	0.125777	C	-1.372851	-1.572090	-0.242445	C	1.630156	-1.471108	0.235977

C	2.106810	3.183724	0.263725	C	-2.022556	3.198440	-0.161303	C	1.626218	3.341909	0.090099
C	-3.106412	-0.028899	0.413447	C	3.157377	-0.110592	0.109061	C	-3.045596	-0.670431	0.214374
C	-4.117783	0.547767	-0.596990	C	3.573490	-1.585303	-0.038041	C	-3.570554	-0.051722	1.522549
C	-3.511985	-1.476852	0.748707	C	3.719776	0.703446	-1.070929	C	-4.061796	-0.450260	-0.919660
C	0.892830	-2.974726	-0.060656	C	-0.895836	-2.977302	-0.048774	C	1.342292	-2.934326	0.096700
O	3.644056	-1.989333	-0.643583	O	-3.701321	-1.984918	0.255560	O	3.949980	-1.585179	-0.422930
O	-1.504065	-0.766231	-1.205087	O	1.215421	-0.802086	1.427507	O	-1.195344	-0.999258	-1.337839
H	2.019242	0.775349	1.830388	H	-1.789056	0.886409	-1.845732	H	1.828314	1.041088	1.785146
H	-1.931689	1.754770	-1.383021	H	1.894570	1.709531	1.712201	H	-2.142631	1.295249	-1.550664
H	-1.706081	2.234147	0.293974	H	1.749188	2.223068	0.044808	H	-2.012755	1.858604	0.109777
H	0.071159	2.960695	-1.287628	H	-0.137611	2.889290	1.553799	H	-0.316068	2.770682	-1.486338
H	0.559586	1.310505	-1.570128	H	-0.620883	1.207966	1.697673	H	0.384500	1.175435	-1.675739
H	0.429124	2.349432	1.274334	H	-0.262211	2.410724	-1.059120	H	0.053132	2.333765	1.107176
H	-1.000374	-1.367850	1.404245	H	1.154516	-1.361841	-1.183476	H	-0.852759	-1.561923	1.366179
H	-0.758912	0.300381	1.919880	H	0.985082	0.316521	-1.696405	H	-0.842203	0.135206	1.829413
H	3.911390	0.527759	0.400801	H	-3.817169	0.601607	-0.623271	H	3.781609	1.015518	0.413324
H	2.936949	0.743956	-1.051565	H	-2.980857	0.691752	0.927529	H	2.827037	0.960627	-1.070073
H	2.780985	3.218939	1.127257	H	-2.612198	3.286039	-1.081195	H	2.264090	3.513120	0.965144
H	1.620446	4.161582	0.179670	H	-1.545595	4.167324	0.023252	H	1.012127	4.237188	-0.055603
H	2.720608	3.043303	-0.632827	H	-2.718897	3.011092	0.663371	H	2.279292	3.244910	-0.784205
H	-3.175266	0.566591	1.335695	H	3.638332	0.270723	1.024895	H	-2.975940	-1.758255	0.379571
H	-5.137608	0.375356	-0.237232	H	4.664753	-1.669103	0.007759	H	-4.580348	-0.423618	1.726901
H	-4.005124	1.625275	-0.742546	H	3.147172	-2.209422	0.750391	H	-2.951840	-0.301695	2.389251
H	-4.016302	0.056494	-1.569994	H	3.260619	-1.998959	-1.003462	H	-3.637204	1.040188	1.455864
H	-4.564238	-1.503980	1.050697	H	4.806087	0.573772	-1.124748	H	-4.992098	-0.984044	-0.696809
H	-2.934307	-1.906342	1.571996	H	3.525907	1.776019	-0.983551	H	-3.678209	-0.814008	-1.876214
H	-3.392013	-2.122885	-0.125818	H	3.307586	0.360186	-2.026658	H	-4.315232	0.609794	-1.031788
H	1.536191	-3.673830	0.485876	H	-1.674721	-3.679113	-0.362746	H	2.191954	-3.517570	0.466277
H	-0.137937	-3.099701	0.276002	H	0.021313	-3.186674	-0.605889	H	0.445853	-3.240492	0.644618
H	0.946777	-3.272120	-1.114045	H	-0.689682	-3.171875	1.010027	H	1.214909	-3.210544	-0.957703
H	-0.577328	-0.797548	-1.480382	H	1.700632	-0.555600	2.227222	H	-1.153010	-1.936786	-1.104834
**3b1**				**3b2**				**3b3**			
C	-1.770417	-0.212244	0.540241	C	-1.774651	-0.215898	0.534670	C	-1.771497	-0.216578	0.542187
C	-1.899710	1.324842	0.680359	C	-1.902943	1.320956	0.674356	C	-1.902927	1.315481	0.688464
C	-1.128638	2.228007	-0.298805	C	-1.122411	2.225405	-0.295855	C	-1.129885	2.217461	-0.287550
C	0.415049	2.172524	-0.265246	C	0.421104	2.173359	-0.254652	C	0.414376	2.175110	-0.246639
C	0.966430	0.810186	-0.763292	C	0.976758	0.814789	-0.756888	C	0.976644	0.820185	-0.750524
C	0.854893	-0.352796	0.219108	C	0.863071	-0.354541	0.217455	C	0.864999	-0.352614	0.219909
C	-0.379454	-0.695578	1.010620	C	-0.376778	-0.701758	1.002401	C	-0.372298	-0.698745	1.008996
C	2.458976	0.820298	-1.158969	C	2.470330	0.827141	-1.148572	C	2.470642	0.836951	-1.140433
C	3.060687	-0.419230	-0.506112	C	3.067673	-0.422090	-0.510743	C	3.067370	-0.418663	-0.515276

C	2.019376	-1.039854	0.339921	C	2.024335	-1.047960	0.330062	C	2.026134	-1.047307	0.326217
C	-2.166590	-0.748005	-0.873878	C	-2.173103	-0.752039	-0.872877	C	-2.168123	-0.747007	-0.873476
C	-1.977089	-2.269920	-1.004077	C	-2.058051	-2.284487	-0.976733	C	-2.075413	-2.280976	-0.989231
C	-3.605902	-0.370649	-1.272909	C	-3.586226	-0.308548	-1.294207	C	-3.570264	-0.281216	-1.304707
C	0.994028	2.613614	1.089518	C	0.994458	2.609895	1.103988	C	0.985211	2.612753	1.112632
C	2.317945	-2.245744	1.179519	C	2.320263	-2.264378	1.155275	C	2.324936	-2.266727	1.146246
O	4.206564	-0.820767	-0.657039	O	4.211676	-0.826760	-0.665851	O	4.208983	-0.826742	-0.679119
O	-2.633857	-0.805817	1.538589	O	-2.732136	-0.810619	1.440057	O	-2.715359	-0.703040	1.523797
H	0.379343	0.553041	-1.656250	H	0.392299	0.561269	-1.652503	H	0.395133	0.567055	-1.648391
H	-1.621173	1.568936	1.712328	H	-1.620732	1.574895	1.706901	H	-1.624503	1.556426	1.720969
H	-2.962965	1.586250	0.598249	H	-2.965939	1.568965	0.592473	H	-2.967526	1.558002	0.614681
H	-1.460749	2.025581	-1.325568	H	-1.448415	2.021472	-1.323978	H	-1.452934	2.012501	-1.316772
H	-1.431223	3.262454	-0.089024	H	-1.428808	3.259134	-0.088277	H	-1.440904	3.250070	-0.081732
H	0.749326	2.908697	-1.010906	H	0.757339	2.913648	-0.995267	H	0.747224	2.917466	-0.986874
H	-0.252701	-0.296226	2.027646	H	-0.236989	-0.303469	2.021280	H	-0.247848	-0.300834	2.026811
H	-0.429016	-1.780193	1.147464	H	-0.424634	-1.787030	1.132980	H	-0.399349	-1.787734	1.139336
H	2.623216	0.795013	-2.240888	H	2.637723	0.817035	-2.230187	H	2.640086	0.840161	-2.221737
H	2.984842	1.704170	-0.780328	H	2.996808	1.704436	-0.755535	H	2.995684	1.709618	-0.735300
H	-1.499596	-0.269574	-1.599958	H	-1.464458	-0.321800	-1.589482	H	-1.446067	-0.333307	-1.586759
H	-2.336627	-2.611237	-1.980706	H	-2.374989	-2.613250	-1.972104	H	-2.339049	-2.593332	-2.004803
H	-0.927165	-2.566809	-0.925562	H	-1.032908	-2.638685	-0.832547	H	-1.070552	-2.662911	-0.786191
H	-2.541353	-2.800665	-0.230475	H	-2.698514	-2.774657	-0.237526	H	-2.779572	-2.784863	-0.314720
H	-3.815639	-0.728032	-2.286280	H	-3.826834	-0.725395	-2.277947	H	-3.827969	-0.717495	-2.275675
H	-3.780678	0.709552	-1.268088	H	-3.681374	0.778810	-1.370692	H	-3.636253	0.805535	-1.408178
H	-4.344549	-0.842358	-0.613187	H	-4.334400	-0.666135	-0.580778	H	-4.326370	-0.595927	-0.578553
H	2.088097	2.644425	1.069764	H	2.088390	2.647075	1.087338	H	2.079056	2.655363	1.096666
H	0.643397	3.619692	1.345218	H	0.637369	3.612245	1.365271	H	0.623003	3.613160	1.373948
H	0.703525	1.945089	1.905645	H	0.706724	1.934685	1.915916	H	0.698420	1.936622	1.923806
H	1.528648	-3.002338	1.110526	H	1.523730	-3.013378	1.087265	H	1.536264	-3.023318	1.065847
H	2.422228	-1.981374	2.239410	H	2.439692	-2.011240	2.216430	H	2.432035	-2.020107	2.210095
H	3.262449	-2.690779	0.855115	H	3.257240	-2.714778	0.816679	H	3.268311	-2.707171	0.812331
H	-3.520800	-0.433351	1.447468	H	-2.597614	-0.443889	2.324545	H	-2.644749	-1.665364	1.582890

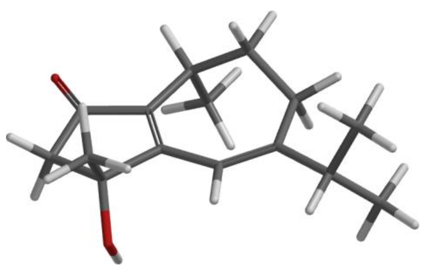

4a1 (54.70%)

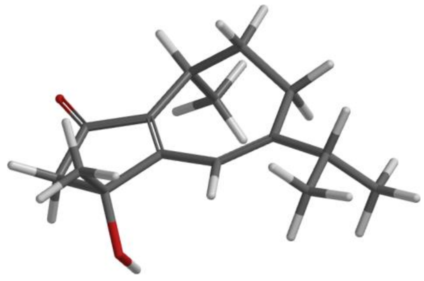

4a2 (17.86%)

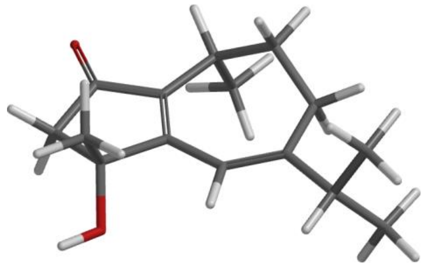

4a3 (14.20%)

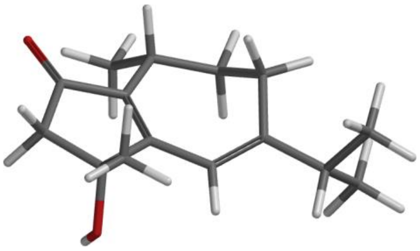

4a4 (7.57%)

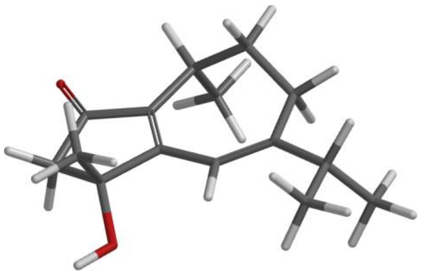

4a5 (3.42%)

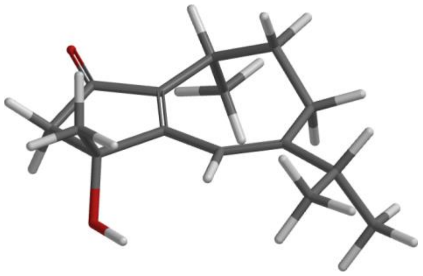

4a6 (2.26%)

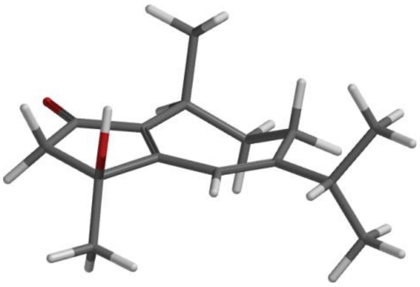

4b1 (46.52%)

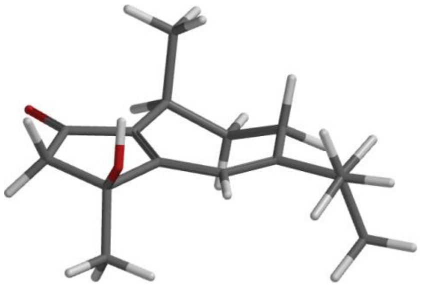

4b2 (28.72%)

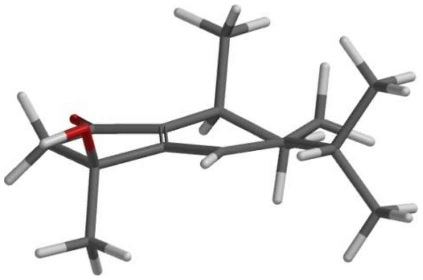

4b3 (12.08%)

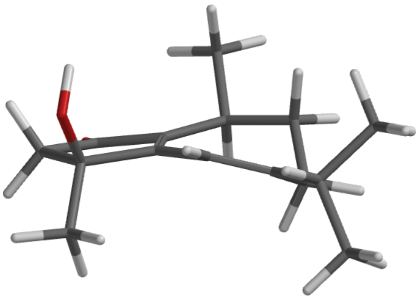

4b4 (6.43%)

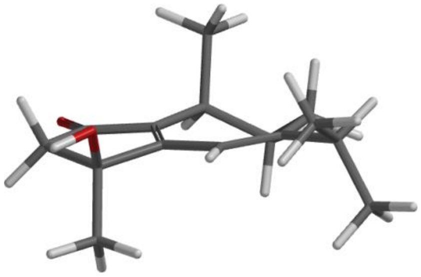

4b5 (6.25%)

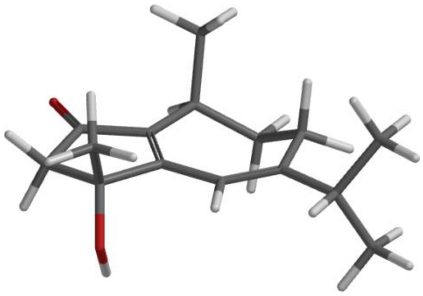

4c1 (53.50%)

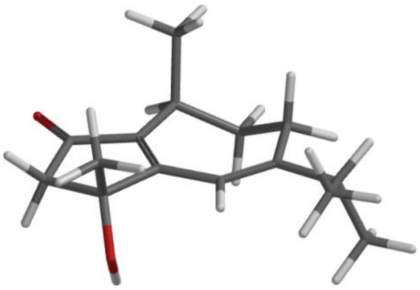

4c2 (21.48%)

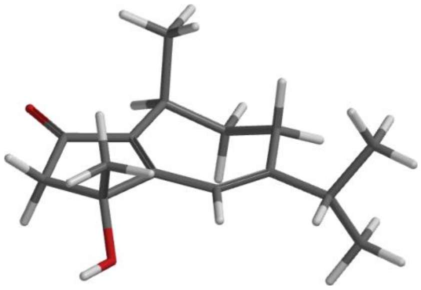

4c3 (11.54%)

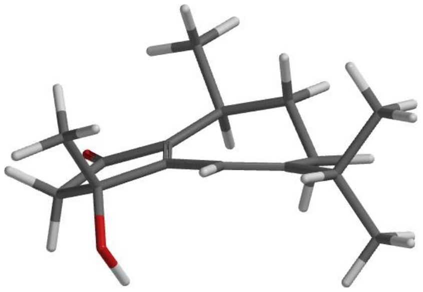

4c4 (5.61%)

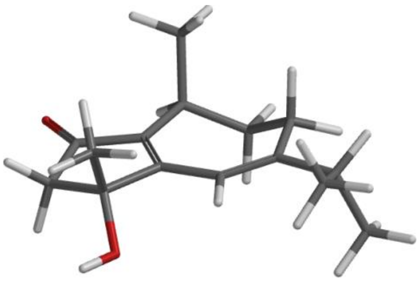

4c5 (5.20%)

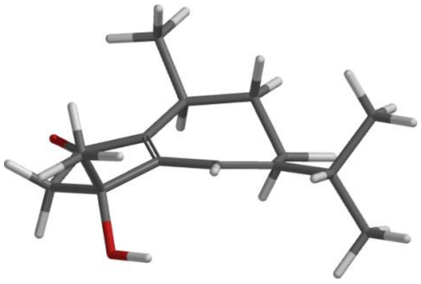

4c6 (2.67%)

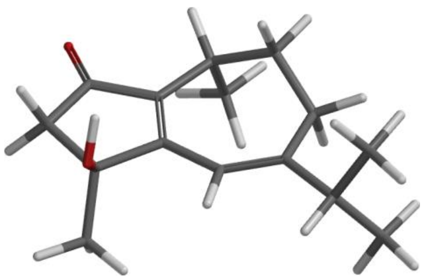

4d1 (53.50%)

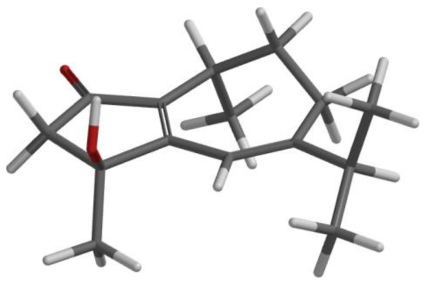

4d2 (21.48%)

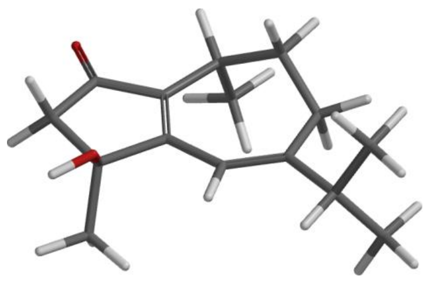

4d3 (11.54%)

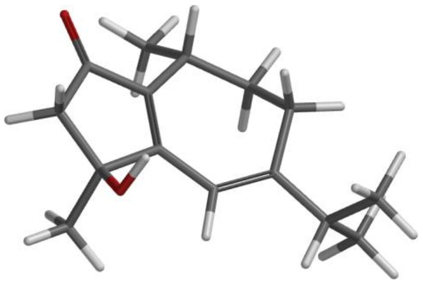

4d4 (5.61%)

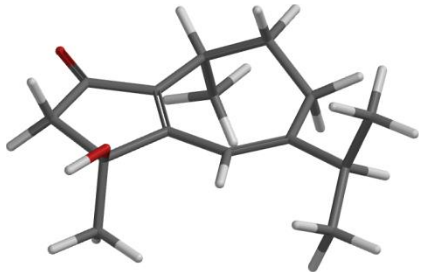

4d5 (5.20%)

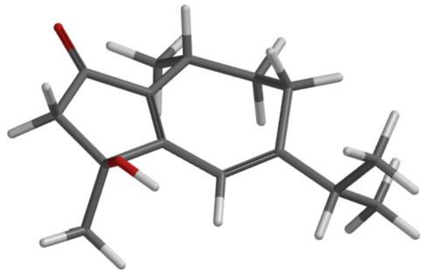

4d6 (2.67%)

Figure S87. Stable conformers of compound **4** with 4*R*,10*R* (**4a**), 4*S*,10*S* (**4b**), 4*R*,10*S* (**4c**) and 4*S*,10*R* (**4d**) configurations, respectively

Table S7. Important thermodynamic parameters (a.u.) of the optimized compound **4** at B3LYP/6-31G (d,p) level in the gas phase

conformations	E+ZPE	G	conformations	E+ZPE	G
**4a1**	-735.004591	-735.049531	**4b1**	-735.004591	-735.049531
**4a2**	-735.003323	-735.048475	**4b2**	-735.003867	-735.049076
**4a3**	-735.003355	-735.048259	**4b3**	-735.003355	-735.048259
**4a4**	-735.002752	-735.047665	**4b4**	-735.002752	-735.047665
**4a5**	-735.002048	-735.046916	**4b5**	-735.002623	-735.047637
**4a6**	-735.001585	-735.046524			
**4c1**	-735.004376	-735.049310	**4d1**	-735.004376	-735.049310
**4c2**	-735.003511	-735.048449	**4d2**	-735.003511	-735.048449
**4c3**	-735.003041	-735.047863	**4d3**	-735.003041	-735.047863
**4c4**	-735.002471	-735.047183	**4d4**	-735.002471	-735.047183
**4c5**	-735.002286	-735.047111	**4d5**	-735.002286	-735.047111
**4c6**	-735.001411	-735.046483	**4d6**	-735.001411	-735.046483

Table S8. Optimized Z-Matrixes of compound **4** in the Gas Phase (Å) at B3LYP/6-31G (d,p) level

**4a1**				**4a2**				**4a3**			
C	-1.823559	-0.12842	0.012413	C	-1.861293	0.221019	-0.225891	C	-1.823287	-0.13192	0.012828
C	-1.869453	1.366382	-0.207714	C	-1.672056	1.718434	-0.348181	C	-1.872208	1.360525	-0.221195
C	-0.723859	2.012432	-1.003886	C	-0.418945	2.216788	-1.088343	C	-0.723841	2.002244	-1.016619
C	0.648888	2.078714	-0.311057	C	0.917676	2.084652	-0.339352	C	0.644171	2.078225	-0.315503
C	1.283908	0.730084	-0.131379	C	1.336959	0.659209	-0.120619	C	1.281444	0.733457	-0.117931

C	0.702663	-0.494941	0.014573	C	0.584725	-0.476822	-0.051302	C	0.702163	-0.490804	0.032626
C	-0.696676	-0.879395	0.098337	C	-0.854649	-0.679734	-0.107531	C	-0.695601	-0.879792	0.109396
C	2.754027	0.593441	-0.112797	C	2.767708	0.321994	0.026689	C	2.75067	0.601954	-0.078041
C	3.101961	-0.862435	0.172407	C	2.886158	-1.165865	0.330753	C	3.094193	-0.847798	0.245761
C	1.75627	-1.61818	0.109151	C	1.466822	-1.732875	0.111079	C	1.757927	-1.607123	0.109981
C	1.685147	-2.573029	-1.086616	C	1.396184	-2.645844	-1.117525	C	1.723983	-2.49262	-1.147472
O	1.52057	-2.447222	1.260974	O	1.007687	-2.546643	1.204713	O	1.446832	-2.39875	1.270386
C	0.618176	2.864589	1.018043	C	0.955232	2.888332	0.979151	C	0.602511	2.876012	1.006235
C	-3.170523	-0.830877	0.159546	C	-3.322464	-0.224709	-0.219805	C	-3.16889	-0.835124	0.16921
C	-4.041716	-0.208233	1.269524	C	-3.562695	-1.732971	-0.36758	C	-4.035544	-0.206128	1.279257
C	-3.942758	-0.887679	-1.176433	C	-4.049481	0.306516	1.038946	C	-3.947591	-0.902739	-1.162345
O	3.56312	1.495409	-0.293475	O	3.705565	1.1035	-0.075775	O	3.562601	1.499417	-0.266831
H	-2.808417	1.608397	-0.719482	H	-2.552108	2.126247	-0.861762	H	-2.809143	1.595783	-0.73996
H	-1.957016	1.855847	0.773457	H	-1.702738	2.163394	0.656693	H	-1.965313	1.858224	0.755231
H	-0.615095	1.491906	-1.96349	H	-0.343047	1.700805	-2.053573	H	-0.60797	1.47337	-1.970934
H	-1.027796	3.039781	-1.241824	H	-0.569797	3.279576	-1.3163	H	-1.028692	3.02677	-1.265562
H	1.321224	2.634442	-0.979248	H	1.692901	2.523126	-0.983469	H	1.319328	2.629293	-0.984878
H	-0.839577	-1.942115	0.283525	H	-1.135188	-1.722984	-0.00795	H	-0.835594	-1.939394	0.310509
H	3.548303	-0.940279	1.170632	H	3.201853	-1.297568	1.372352	H	3.448574	-0.900574	1.281194
H	3.849369	-1.226703	-0.538906	H	3.653354	-1.630462	-0.295745	H	3.893815	-1.212926	-0.406237
H	0.700398	-3.042853	-1.154932	H	0.370818	-2.97657	-1.302919	H	0.740762	-2.954501	-1.273824
H	2.434405	-3.361603	-0.967392	H	2.016817	-3.530939	-0.947761	H	2.473767	-3.289997	-1.068702
H	1.881844	-2.036063	-2.019328	H	1.761197	-2.122906	-2.006462	H	1.951601	-1.907159	-2.043939
H	1.513124	-1.889862	2.051646	H	0.989367	-2.009383	2.00898	H	2.01401	-3.181703	1.27202
H	1.631973	3.001081	1.404955	H	1.959542	2.86679	1.411113	H	1.613434	3.020189	1.397689
H	0.178279	3.85721	0.864406	H	0.688762	3.935882	0.794771	H	0.15918	3.865204	0.840362
H	0.032158	2.344866	1.78345	H	0.259521	2.483802	1.722028	H	0.015913	2.35985	1.773169
H	-2.959484	-1.86681	0.452427	H	-3.790715	0.26518	-1.088079	H	-2.95497	-1.868463	0.46906
H	-4.957364	-0.794794	1.401508	H	-4.637064	-1.927533	-0.451384	H	-4.950078	-0.792651	1.419649
H	-3.512395	-0.188821	2.228172	H	-3.07885	-2.136595	-1.262674	H	-3.50086	-0.180017	2.234614
H	-4.341248	0.816956	1.025779	H	-3.196757	-2.292764	0.500432	H	-4.337189	0.81715	1.029936
H	-4.867401	-1.46219	-1.051718	H	-5.113142	0.04771	1.000313	H	-4.870605	-1.478164	-1.029345
H	-3.346763	-1.369396	-1.958151	H	-3.975796	1.393853	1.132885	H	-3.354391	-1.388489	-1.943847
H	-4.223018	0.110784	-1.530161	H	-3.624231	-0.142262	1.944078	H	-4.231646	0.092641	-1.521774
**4a4**				**4a5**				**4a6**			
C	-1.843813	-0.145803	-0.068991	C	-1.861357	0.205352	-0.233339	C	-1.86588	0.252491	-0.192322
C	-1.68268	1.169248	-0.800295	C	-1.687432	1.701052	-0.391762	C	-1.637114	1.748506	-0.200013
C	-0.836891	2.198222	-0.019347	C	-0.423019	2.20095	-1.109909	C	-0.434704	2.252248	-1.020485
C	0.685062	2.073936	-0.20376	C	0.897755	2.089648	-0.329444	C	0.947752	2.074059	-0.373553
C	1.236271	0.668861	-0.118411	C	1.330747	0.67037	-0.101147	C	1.335357	0.639146	-0.152238
C	0.630184	-0.525257	0.150772	C	0.587681	-0.47045	-0.03213	C	0.571538	-0.486584	-0.068058

C	-0.772538	-0.850085	0.363115	C	-0.848996	-0.685375	-0.092221	C	-0.871143	-0.666572	-0.142953
C	2.678282	0.433857	-0.371132	C	2.761596	0.347885	0.064439	C	2.764112	0.280621	-0.018972
C	3.003497	-1.018651	-0.048334	C	2.884195	-1.135691	0.391219	C	2.866218	-1.203533	0.296368
C	1.638413	-1.693316	0.193917	C	1.483153	-1.714307	0.10546	C	1.439779	-1.749526	0.133933
C	1.329716	-2.768001	-0.851619	C	1.463136	-2.557941	-1.181145	C	1.321408	-2.73219	-1.040622
O	1.558582	-2.371607	1.461566	O	0.964965	-2.487844	1.202087	O	1.099095	-2.413902	1.366595
C	1.401763	3.011993	0.795688	C	0.891816	2.891944	0.990244	C	1.113032	2.898928	0.922673
C	-3.248566	-0.672973	0.176461	C	-3.319228	-0.251628	-0.212596	C	-3.334367	-0.16269	-0.225552
C	-4.068088	0.266022	1.086694	C	-3.547426	-1.765052	-0.322549	C	-3.607386	-1.647657	-0.498142
C	-3.997331	-0.950877	-1.143601	C	-4.046699	0.302423	1.036184	C	-4.051725	0.280321	1.072016
O	3.482101	1.257383	-0.789772	O	3.696396	1.132852	-0.037546	O	3.711277	1.046254	-0.146966
H	-1.215023	0.986871	-1.778981	H	-2.559284	2.082443	-0.93917	H	-2.543471	2.228594	-0.588494
H	-2.666659	1.597019	-1.012462	H	-1.752021	2.171597	0.59976	H	-1.547595	2.102853	0.83654
H	-1.122582	3.211797	-0.326388	H	-0.320416	1.674261	-2.067012	H	-0.442667	1.764033	-2.002844
H	-1.08337	2.121148	1.04691	H	-0.578737	3.259482	-1.354219	H	-0.578724	3.324163	-1.2051
H	0.940446	2.436006	-1.21106	H	1.681152	2.539285	-0.955694	H	1.688202	2.473526	-1.081267
H	-0.947944	-1.799692	0.864858	H	-1.122064	-1.726983	0.035421	H	-1.184099	-1.707364	-0.145698
H	3.631567	-1.05832	0.849272	H	3.11865	-1.241154	1.456366	H	3.195861	-1.333472	1.332271
H	3.580526	-1.471573	-0.860087	H	3.693894	-1.596343	-0.183109	H	3.603848	-1.686311	-0.351165
H	0.333769	-3.190358	-0.695973	H	0.447365	-2.885658	-1.419523	H	0.293361	-3.088676	-1.169524
H	2.063809	-3.575512	-0.77182	H	2.094175	-3.447411	-1.059548	H	1.962216	-3.599706	-0.855508
H	1.376773	-2.346236	-1.860281	H	1.852439	-1.985633	-2.02913	H	1.631109	-2.25944	-1.978543
H	1.723672	-1.732479	2.168906	H	1.428632	-3.335756	1.228241	H	0.195869	-2.754064	1.308403
H	2.477103	3.037323	0.611725	H	1.887293	2.889488	1.442745	H	2.142437	2.839453	1.285261
H	1.012592	4.031668	0.698406	H	0.608128	3.934199	0.801104	H	0.880614	3.953586	0.732939
H	1.227006	2.683308	1.827204	H	0.190761	2.47112	1.718341	H	0.453618	2.539775	1.719742
H	-3.14446	-1.630172	0.703078	H	-3.794862	0.213072	-1.090681	H	-3.788929	0.406592	-1.051609
H	-5.05391	-0.168526	1.285969	H	-4.620376	-1.970414	-0.399444	H	-4.685114	-1.810918	-0.601623
H	-3.567621	0.424923	2.047439	H	-3.061856	-2.186655	-1.208556	H	-3.127576	-1.98792	-1.42152
H	-4.229097	1.245418	0.622325	H	-3.174993	-2.299044	0.558801	H	-3.262346	-2.28515	0.324366
H	-4.982845	-1.382224	-0.935979	H	-5.107009	0.028325	1.010368	H	-5.122697	0.0592	1.008739
H	-3.443777	-1.655957	-1.772281	H	-3.987697	1.392755	1.100861	H	-3.94379	1.352729	1.258255
H	-4.155116	-0.033701	-1.722077	H	-3.609583	-0.116118	1.949926	H	-3.644363	-0.25571	1.936872
**4b1**				**4b2**				**4b3**			
C	1.823559	-0.12842	0.012413	C	1.848647	0.333806	0.094707	C	1.823287	-0.13192	0.012828
C	1.869453	1.366382	-0.207714	C	1.64147	1.821519	-0.079747	C	1.872208	1.360525	-0.221195
C	0.723859	2.012432	-1.003886	C	0.404064	2.310891	-0.845856	C	0.723841	2.002244	-1.016619
C	-0.648888	2.078714	-0.311057	C	-0.961567	2.092537	-0.17016	C	-0.644171	2.078225	-0.315503
C	-1.283908	0.730084	-0.131379	C	-1.354226	0.645215	-0.097097	C	-1.281444	0.733457	-0.117931
C	-0.702663	-0.494941	0.014573	C	-0.569467	-0.466035	-0.002412	C	-0.702163	-0.490804	0.032626
C	0.696676	-0.879395	0.098337	C	0.872881	-0.606768	0.115245	C	0.695601	-0.879792	0.109396

C	-2.754027	0.593441	-0.112797	C	-2.776852	0.253113	-0.138113	C	-2.75067	0.601954	-0.078041
C	-3.101961	-0.862435	0.172407	C	-2.869215	-1.255851	0.053236	C	-3.094193	-0.847798	0.245761
C	-1.75627	-1.61818	0.109151	C	-1.410661	-1.759504	-0.014005	C	-1.757927	-1.607123	0.109981
C	-1.685147	-2.573029	-1.086616	C	-1.145812	-2.600993	-1.266425	C	-1.723983	-2.49262	-1.147472
O	-1.52057	-2.447222	1.260974	O	-1.056953	-2.613277	1.088506	O	-1.446832	-2.39875	1.270386
C	-0.618176	2.864589	1.018043	C	-1.067674	2.772045	1.212721	C	-0.602511	2.876012	1.006235
C	3.170523	-0.830877	0.159546	C	3.3172	-0.060088	0.250334	C	3.16889	-0.835124	0.16921
C	4.041716	-0.208233	1.269524	C	3.996155	-0.172116	-1.134903	C	4.035544	-0.206128	1.279257
C	3.942758	-0.887679	-1.176433	C	3.57428	-1.33016	1.075581	C	3.947591	-0.902739	-1.162345
O	-3.56312	1.495409	-0.293475	O	-3.728119	1.009083	-0.295112	O	-3.562601	1.499417	-0.266831
H	1.957016	1.855847	0.773457	H	1.667679	2.280612	0.920768	H	1.965313	1.858224	0.755231
H	2.808417	1.608397	-0.719482	H	2.52894	2.224247	-0.585332	H	2.809143	1.595783	-0.73996
H	1.027796	3.039781	-1.241824	H	0.527009	3.388538	-1.013751	H	1.028692	3.02677	-1.265562
H	0.615095	1.491906	-1.96349	H	0.388412	1.843447	-1.838271	H	0.60797	1.47337	-1.970934
H	-1.321224	2.634442	-0.979248	H	-1.718239	2.571713	-0.806421	H	-1.319328	2.629293	-0.984878
H	0.839577	-1.942115	0.283525	H	1.18534	-1.635399	0.268612	H	0.835594	-1.939394	0.310509
H	-3.849369	-1.226703	-0.538906	H	-3.528419	-1.701658	-0.69761	H	-3.893815	-1.212926	-0.406237
H	-3.548303	-0.940279	1.170632	H	-3.311313	-1.470098	1.033203	H	-3.448574	-0.900574	1.281194
H	-0.700398	-3.042853	-1.154932	H	-0.090891	-2.880055	-1.334096	H	-0.740762	-2.954501	-1.273824
H	-2.434405	-3.361603	-0.967392	H	-1.741937	-3.517628	-1.222494	H	-2.473767	-3.289997	-1.068702
H	-1.881844	-2.036063	-2.019328	H	-1.418418	-2.043979	-2.167759	H	-1.951601	-1.907159	-2.043939
H	-1.513124	-1.889862	2.051646	H	-1.172937	-2.123165	1.914454	H	-2.01401	-3.181703	1.27202
H	-1.631973	3.001081	1.404955	H	-2.089628	2.701665	1.596215	H	-1.613434	3.020189	1.397689
H	-0.178279	3.85721	0.864406	H	-0.80809	3.834849	1.1377	H	-0.15918	3.865204	0.840362
H	-0.032158	2.344866	1.78345	H	-0.399765	2.307148	1.945671	H	-0.015913	2.35985	1.773169
H	2.959484	-1.86681	0.452427	H	3.802402	0.774195	0.779318	H	2.95497	-1.868463	0.46906
H	4.957364	-0.794794	1.401508	H	5.074166	-0.332387	-1.021966	H	4.950078	-0.792651	1.419649
H	3.512395	-0.188821	2.228172	H	3.854048	0.731239	-1.736714	H	3.50086	-0.180017	2.234614
H	4.341248	0.816956	1.025779	H	3.582356	-1.017668	-1.695224	H	4.337189	0.81715	1.029936
H	4.867401	-1.46219	-1.051718	H	4.649867	-1.452142	1.242708	H	4.870605	-1.478164	-1.029345
H	3.346763	-1.369396	-1.958151	H	3.081539	-1.283177	2.051929	H	3.354391	-1.388489	-1.943847
H	4.223018	0.110784	-1.530161	H	3.223128	-2.232412	0.563272	H	4.231646	0.092641	-1.521774
**4b4**				**4b5**				**4c1**			
C	1.843813	-0.145803	-0.068991	C	-1.849249	0.329682	-0.093585	C	1.827934	-0.127131	-0.00268
C	1.68268	1.169248	-0.800295	C	-1.646486	1.815971	0.095992	C	1.86275	1.382528	-0.076828
C	0.836891	2.198222	-0.019347	C	-0.406295	2.301251	0.859805	C	0.749259	2.080121	-0.87644
C	-0.685062	2.073936	-0.20376	C	0.955347	2.093305	0.173091	C	-0.655755	2.089488	-0.248461
C	-1.236271	0.668861	-0.118411	C	1.352071	0.648389	0.082689	C	-1.282071	0.72658	-0.166999
C	-0.630184	-0.525257	0.150772	C	0.569963	-0.463259	-0.016304	C	-0.696259	-0.499508	-0.047288
C	0.772538	-0.850085	0.363115	C	-0.871859	-0.608453	-0.126283	C	0.703753	-0.886452	0.006347
C	-2.678282	0.433857	-0.371132	C	2.775567	0.261015	0.100852	C	-2.750489	0.577024	-0.2248

C	-3.003497	-1.018651	-0.048335	C	2.865777	-1.243055	-0.131532	C	-3.089374	-0.908179	-0.200297
C	-1.638413	-1.693316	0.193917	C	1.415363	-1.748327	0.013479	C	-1.746138	-1.625658	0.055734
C	-1.329716	-2.768001	-0.851619	C	1.202476	-2.519689	1.327266	C	-1.712429	-2.318577	1.421859
O	-1.558582	-2.371607	1.461566	O	0.993227	-2.557318	-1.098762	O	-1.474316	-2.669681	-0.896193
C	-1.401763	3.011993	0.795688	C	1.050125	2.785408	-1.204454	C	-0.698241	2.797861	1.123683
C	3.248566	-0.672973	0.176461	C	-3.316633	-0.065866	-0.256614	C	3.177373	-0.833559	0.085439
C	4.068088	0.266022	1.086694	C	-4.001331	-0.190292	1.124576	C	3.971633	-0.735813	-1.235012
C	3.997331	-0.950877	-1.143601	C	-3.567723	-1.329654	-1.093299	O	-3.564352	1.489735	-0.29885
O	-3.482101	1.257383	-0.789772	O	3.7283	1.01301	0.266247	C	4.024245	-0.331643	1.273016
H	2.666659	1.597019	-1.012462	H	-1.679295	2.285393	-0.899484	H	1.887768	1.780628	0.948038
H	1.215023	0.986871	-1.778981	H	-2.532488	2.210538	0.610762	H	2.822816	1.682316	-0.512633
H	1.08337	2.121148	1.04691	H	-0.530982	3.376893	1.039189	H	1.053251	3.124252	-1.023802
H	1.122582	3.211797	-0.326388	H	-0.383357	1.823937	1.847511	H	0.6924	1.630929	-1.875841
H	-0.940446	2.436006	-1.21106	H	1.714647	2.568794	0.809151	H	-1.301027	2.677726	-0.914954
H	0.947944	-1.799692	0.864858	H	-1.180499	-1.635008	-0.295841	H	0.850197	-1.963301	0.05953
H	-3.580526	-1.471573	-0.860087	H	3.580614	-1.702858	0.557812	H	-3.511412	-1.199958	-1.1687
H	-3.631567	-1.05832	0.849272	H	3.217378	-1.417319	-1.154555	H	-3.85344	-1.116866	0.554785
H	-0.333769	-3.190358	-0.695973	H	0.1498	-2.786299	1.457143	H	-0.730638	-2.758679	1.615698
H	-2.063809	-3.575512	-0.77182	H	1.796006	-3.442804	1.325668	H	-2.457384	-3.119952	1.440652
H	-1.376773	-2.346236	-1.860281	H	1.518192	-1.920576	2.187251	H	-1.940053	-1.605492	2.219793
H	-1.723672	-1.732479	2.168906	H	1.418213	-3.422907	-1.029945	H	-1.434098	-2.283816	-1.78239
H	-2.477103	3.037323	0.611725	H	2.07014	2.723287	-1.594321	H	-1.73056	2.895107	1.470832
H	-1.012592	4.031668	0.698406	H	0.785425	3.846202	-1.118223	H	-0.269705	3.804391	1.046246
H	-1.227006	2.683308	1.827204	H	0.381865	2.322322	-1.9378	H	-0.137363	2.243543	1.883515
H	3.14446	-1.630172	0.703078	H	-3.801521	0.771577	-0.780972	H	2.970463	-1.897037	0.257796
H	5.05391	-0.168526	1.285969	H	-5.078517	-0.352744	1.006153	H	4.900787	-1.311482	-1.158228
H	3.567621	0.424923	2.047439	H	-3.864218	0.708628	1.734179	H	3.394503	-1.134806	-2.075342
H	4.229097	1.245418	0.622325	H	-3.58752	-1.039133	1.680109	H	4.246167	0.298141	-1.471986
H	4.982845	-1.382224	-0.935979	H	-4.642066	-1.450032	-1.269967	H	4.941623	-0.924334	1.357263
H	3.443777	-1.655957	-1.772281	H	-3.06549	-1.276662	-2.064236	H	3.477159	-0.419936	2.217627
H	4.155116	-0.0337	-1.722077	H	-3.22121	-2.235329	-0.583545	H	4.320739	0.715712	1.148811
**4c2**				**4c3**				**4c4**			
C	1.854056	0.319465	0.126892	C	1.828804	-0.129519	-0.004371	C	1.854544	-0.136043	-0.081521
C	1.638394	1.815851	0.087208	C	1.864317	1.381068	-0.057778	C	1.707964	1.231601	-0.710821
C	0.443084	2.357951	-0.711156	C	0.75855	2.086791	-0.861016	C	0.824146	2.19529	0.109364
C	-0.957129	2.09865	-0.126946	C	-0.651703	2.09038	-0.245337	C	-0.687476	2.077067	-0.144221
C	-1.344607	0.647494	-0.138075	C	-1.278406	0.726819	-0.181072	C	-1.228843	0.665465	-0.153842
C	-0.564298	-0.467428	-0.041015	C	-0.693326	-0.499377	-0.070213	C	-0.622327	-0.532952	0.103407
C	0.877545	-0.618914	0.06968	C	0.705002	-0.88882	-0.011908	C	0.779391	-0.868266	0.294739
C	-2.761971	0.257396	-0.279515	C	-2.745056	0.577878	-0.255105	C	-2.652035	0.425519	-0.489414
C	-2.84514	-1.262933	-0.334482	C	-3.07847	-0.909757	-0.270731	C	-2.929442	-1.070567	-0.417036

C	-1.411461	-1.757417	-0.044513	C	-1.745263	-1.615819	0.050093	C	-1.63758	-1.692326	0.150737
C	-1.319164	-2.509943	1.28674	C	-1.744525	-2.22969	1.460785	C	-1.824964	-2.219386	1.577994
O	-0.925174	-2.686056	-1.030375	O	-1.400842	-2.622648	-0.91835	O	-1.177636	-2.825604	-0.604096
C	-1.146995	2.711196	1.278197	C	-0.707713	2.789152	1.131151	C	-1.449014	2.949014	0.882622
C	3.323387	-0.084152	0.238778	C	3.177732	-0.837949	0.074548	C	3.255166	-0.692226	0.119697
C	3.588754	-1.439515	0.910565	C	3.970284	-0.724463	-1.245645	C	4.007333	-0.852964	-1.21787
O	-3.714809	1.023546	-0.358205	O	-3.562112	1.488422	-0.315514	O	-3.473971	1.274448	-0.812808
C	4.001165	-0.033958	-1.150934	C	4.026171	-0.352223	1.267657	C	4.076821	0.154674	1.114598
H	1.589541	2.176181	1.126382	H	1.878927	1.766811	0.972053	H	2.696496	1.678468	-0.851183
H	2.548669	2.275126	-0.318381	H	2.828369	1.686426	-0.480663	H	1.281098	1.122347	-1.719244
H	0.571182	3.444302	-0.800555	H	1.063751	3.132389	-0.995452	H	1.028606	2.041015	1.176072
H	0.484304	1.957632	-1.731954	H	0.709796	1.646045	-1.864283	H	1.114496	3.229678	-0.111596
H	-1.678066	2.606936	-0.781277	H	-1.291068	2.682706	-0.913879	H	-0.904597	2.498664	-1.136966
H	1.192606	-1.657218	0.10645	H	0.850177	-1.966149	0.020068	H	0.961721	-1.862391	0.696664
H	-3.174606	-1.572871	-1.332766	H	-3.412965	-1.181468	-1.277966	H	-3.132438	-1.450784	-1.424631
H	-3.592032	-1.633096	0.374631	H	-3.890563	-1.133542	0.428133	H	-3.823107	-1.267011	0.183361
H	-0.286333	-2.789366	1.510558	H	-0.764328	-2.647585	1.706768	H	-0.878384	-2.577533	1.991969
H	-1.916885	-3.424788	1.228676	H	-2.488725	-3.033761	1.523484	H	-2.53064	-3.055682	1.564853
H	-1.699161	-1.890666	2.104705	H	-2.001268	-1.477713	2.213693	H	-2.218374	-1.433719	2.229524
H	-0.910539	-2.248068	-1.892808	H	-1.972495	-3.390406	-0.782557	H	-0.970837	-2.54139	-1.505241
H	-2.188101	2.614777	1.598525	H	-1.743232	2.881383	1.470265	H	-2.517663	2.970904	0.664082
H	-0.893496	3.778051	1.265847	H	-0.281164	3.797343	1.064485	H	-1.069284	3.976557	0.855894
H	-0.51706	2.21689	2.025097	H	-0.151502	2.231181	1.891862	H	-1.303937	2.562967	1.898547
H	3.804933	0.686432	0.859906	H	2.969861	-1.903429	0.233227	H	3.144137	-1.692912	0.556248
H	4.665468	-1.574687	1.058961	H	4.899553	-1.301219	-1.177353	H	4.988267	-1.310105	-1.047496
H	3.100428	-1.507165	1.887983	H	3.390901	-1.112481	-2.089367	H	3.451279	-1.492535	-1.911123
H	3.240226	-2.277382	0.297007	H	4.244665	0.31236	-1.470127	H	4.17514	0.112667	-1.707975
H	5.079953	-0.201118	-1.05759	H	4.943581	-0.946174	1.343396	H	5.058283	-0.30403	1.278202
H	3.854856	0.930984	-1.647122	H	3.47998	-0.452327	2.211719	H	3.572514	0.230056	2.083427
H	3.590253	-0.813268	-1.802191	H	4.322994	0.696591	1.157067	H	4.247967	1.170624	0.741244
**4c5**				**4c6**				**4d1**			
C	1.856463	0.313866	0.130108	C	1.838852	-0.163258	-0.075752	C	-1.827934	-0.127131	-0.00268
C	1.642802	1.810997	0.110612	C	1.676073	1.149637	-0.808511	C	-1.86275	1.382528	-0.076828
C	0.456494	2.363519	-0.694235	C	0.866696	2.187804	0.000625	C	-0.749259	2.080121	-0.87644
C	-0.949575	2.100645	-0.126155	C	-0.660666	2.081761	-0.152895	C	0.655755	2.089488	-0.248461
C	-1.338709	0.650282	-0.155633	C	-1.221457	0.678655	-0.125701	C	1.282071	0.72658	-0.166999
C	-0.560385	-0.46522	-0.062254	C	-0.637673	-0.524378	0.143789	C	0.696259	-0.499508	-0.047288
C	0.879191	-0.622191	0.056964	C	0.762945	-0.856719	0.364066	C	-0.703753	-0.886452	0.006347
C	-2.753241	0.26285	-0.319704	C	-2.653628	0.460903	-0.443935	C	2.750489	0.577024	-0.2248
C	-2.827511	-1.256615	-0.42263	C	-2.963833	-1.024821	-0.332586	C	3.089374	-0.908179	-0.200297
C	-1.414628	-1.74502	-0.044885	C	-1.660269	-1.679866	0.144944	C	1.746138	-1.625658	0.055734

C	-1.387884	-2.410358	1.342091	C	-1.797071	-2.333756	1.528188	C	1.712429	-2.318577	1.421859
O	-0.853566	-2.638625	-1.023244	O	-1.304852	-2.675799	-0.83411	O	1.474316	-2.669681	-0.896193
C	-1.1536	2.701016	1.282177	C	-1.351444	2.971897	0.90689	C	0.698241	2.797861	1.123683
C	3.324996	-0.094299	0.233801	C	3.242341	-0.691022	0.172757	C	-3.177373	-0.833559	0.085439
C	3.588217	-1.461286	0.882497	C	3.985735	-0.98607	-1.1467	C	-4.024245	-0.331643	1.273016
O	-3.709305	1.025818	-0.385527	O	-3.458981	1.323613	-0.769308	O	3.564352	1.489735	-0.29885
C	3.999999	-0.024189	-1.156397	C	4.066732	0.25686	1.069129	C	-3.971633	-0.735813	-1.235012
H	1.582496	2.157201	1.154045	H	2.658574	1.56246	-1.053744	H	-2.822816	1.682316	-0.512633
H	2.557445	2.27541	-0.278859	H	1.170995	0.969152	-1.768537	H	-1.887768	1.780628	0.948038
H	0.58696	3.450589	-0.771296	H	1.13653	2.097633	1.060062	H	-0.6924	1.630929	-1.875841
H	0.506564	1.972076	-1.717785	H	1.160618	3.199542	-0.304369	H	-1.053251	3.124252	-1.023802
H	-1.662822	2.615566	-0.78368	H	-0.935806	2.493351	-1.135145	H	1.301027	2.677726	-0.914954
H	1.191245	-1.661397	0.074827	H	0.953865	-1.798538	0.87917	H	-0.850197	-1.963301	0.05953
H	-3.048563	-1.523981	-1.461814	H	-3.241919	-1.417856	-1.315112	H	3.85344	-1.116866	0.554785
H	-3.629553	-1.652151	0.208387	H	-3.811983	-1.182698	0.340961	H	3.511412	-1.199958	-1.1687
H	-0.365908	-2.667667	1.634034	H	-0.849936	-2.76856	1.866283	H	0.730638	-2.758679	1.615698
H	-1.986411	-3.330201	1.331773	H	-2.5389	-3.13655	1.47751	H	2.457384	-3.119952	1.440652
H	-1.810152	-1.745371	2.102161	H	-2.11871	-1.602671	2.277393	H	1.940053	-1.605492	2.219793
H	-1.295396	-3.495272	-0.948413	H	-0.416958	-3.007104	-0.640538	H	1.434098	-2.283816	-1.78239
H	-2.197961	2.601956	1.591132	H	-2.430707	3.005042	0.7486	H	1.73056	2.895107	1.470832
H	-0.899739	3.76793	1.282	H	-0.963346	3.995115	0.848705	H	0.269705	3.804391	1.046246
H	-0.531108	2.20021	2.031114	H	-1.153215	2.593397	1.916678	H	0.137363	2.243543	1.883515
H	3.809681	0.664691	0.866776	H	3.13869	-1.642183	0.711338	H	-2.970463	-1.897037	0.257796
H	4.664795	-1.601489	1.027641	H	4.969921	-1.41936	-0.937152	H	-4.941623	-0.924334	1.357263
H	3.100512	-1.544506	1.859168	H	3.427428	-1.694321	-1.767515	H	-3.477159	-0.419936	2.217627
H	3.237127	-2.287213	0.254367	H	4.145535	-0.07544	-1.734478	H	-4.320739	0.715712	1.148811
H	5.078118	-0.199064	-1.068383	H	5.052722	-0.176906	1.268847	H	-4.900787	-1.311482	-1.158228
H	3.858031	0.950073	-1.635206	H	3.570717	0.427902	2.030053	H	-3.394503	-1.134806	-2.075342
H	3.581829	-0.788908	-1.819973	H	4.226549	1.230324	0.592483	H	-4.246167	0.298141	-1.471986
**4d2**				**4d3**				**4d4**			
C	-1.854056	0.319465	0.126892	C	-1.828804	-0.129519	-0.004371	C	-1.854544	-0.136043	-0.081521
C	-1.638394	1.815851	0.087208	C	-1.864317	1.381068	-0.057778	C	-1.707964	1.231601	-0.710821
C	-0.443084	2.357951	-0.711156	C	-0.75855	2.086791	-0.861016	C	-0.824146	2.19529	0.109364
C	0.957129	2.09865	-0.126946	C	0.651703	2.09038	-0.245337	C	0.687476	2.077067	-0.144221
C	1.344607	0.647494	-0.138075	C	1.278406	0.726819	-0.181072	C	1.228843	0.665465	-0.153842
C	0.564298	-0.467428	-0.041015	C	0.693326	-0.499377	-0.070213	C	0.622327	-0.532952	0.103407
C	-0.877545	-0.618914	0.06968	C	-0.705002	-0.88882	-0.011908	C	-0.779391	-0.868266	0.294739
C	2.761971	0.257396	-0.279515	C	2.745056	0.577878	-0.255105	C	2.652035	0.425519	-0.489414
C	2.84514	-1.262933	-0.334482	C	3.07847	-0.909757	-0.270731	C	2.929442	-1.070567	-0.417036
C	1.411461	-1.757417	-0.044513	C	1.745263	-1.615819	0.050093	C	1.63758	-1.692326	0.150737
C	1.319164	-2.509943	1.28674	C	1.744525	-2.22969	1.460785	C	1.824964	-2.219386	1.577993

O	0.925174	-2.686056	-1.030375	O	1.400842	-2.622648	-0.91835	O	1.177636	-2.825604	-0.604096
C	1.146995	2.711196	1.278197	C	0.707713	2.789152	1.131151	C	1.449014	2.949014	0.882622
C	-3.323387	-0.084152	0.238778	C	-3.177732	-0.837949	0.074548	C	-3.255166	-0.692226	0.119697
C	-4.001165	-0.033958	-1.150934	C	-4.026171	-0.352223	1.267657	C	-4.076821	0.154674	1.114598
O	3.714809	1.023546	-0.358205	O	3.562112	1.488422	-0.315514	O	3.473971	1.274448	-0.812808
C	-3.588754	-1.439515	0.910565	C	-3.970284	-0.724463	-1.245645	C	-4.007333	-0.852964	-1.21787
H	-2.548669	2.275126	-0.318381	H	-2.828369	1.686426	-0.480663	H	-1.281098	1.122347	-1.719244
H	-1.589541	2.176181	1.126382	H	-1.878927	1.766811	0.972053	H	-2.696496	1.678468	-0.851183
H	-0.484304	1.957632	-1.731954	H	-0.709796	1.646045	-1.864283	H	-1.114496	3.229678	-0.111596
H	-0.571182	3.444302	-0.800555	H	-1.063751	3.132389	-0.995452	H	-1.028606	2.041015	1.176072
H	1.678066	2.606936	-0.781277	H	1.291068	2.682706	-0.913879	H	0.904597	2.498664	-1.136966
H	-1.192606	-1.657218	0.10645	H	-0.850177	-1.966149	0.020068	H	-0.961721	-1.862391	0.696664
H	3.592032	-1.633096	0.374631	H	3.890563	-1.133542	0.428133	H	3.823107	-1.267011	0.183361
H	3.174606	-1.572871	-1.332766	H	3.412965	-1.181468	-1.277966	H	3.132438	-1.450784	-1.424631
H	0.286333	-2.789366	1.510558	H	0.764328	-2.647585	1.706768	H	0.878384	-2.577533	1.991969
H	1.916885	-3.424788	1.228676	H	2.488725	-3.033761	1.523484	H	2.53064	-3.055682	1.564853
H	1.699161	-1.890666	2.104705	H	2.001268	-1.477713	2.213693	H	2.218374	-1.433719	2.229524
H	0.910539	-2.248068	-1.892808	H	1.972495	-3.390406	-0.782557	H	0.970837	-2.54139	-1.505241
H	2.188101	2.614777	1.598525	H	1.743232	2.881383	1.470265	H	2.517663	2.970904	0.664082
H	0.893496	3.778051	1.265847	H	0.281164	3.797343	1.064485	H	1.069284	3.976557	0.855894
H	0.51706	2.21689	2.025097	H	0.151502	2.231181	1.891862	H	1.303937	2.562967	1.898547
H	-3.804933	0.686432	0.859906	H	-2.969861	-1.903429	0.233227	H	-3.144137	-1.692912	0.556248
H	-5.079953	-0.201118	-1.05759	H	-4.943581	-0.946174	1.343396	H	-5.058283	-0.30403	1.278202
H	-3.854856	0.930984	-1.647122	H	-3.47998	-0.452327	2.211719	H	-3.572514	0.230056	2.083427
H	-3.590253	-0.813268	-1.802191	H	-4.322994	0.696591	1.157067	H	-4.247967	1.170624	0.741244
H	-4.665468	-1.574687	1.058961	H	-4.899553	-1.301219	-1.177353	H	-4.988267	-1.310105	-1.047496
H	-3.100428	-1.507165	1.887983	H	-3.390901	-1.112481	-2.089367	H	-3.451279	-1.492535	-1.911123
H	-3.240226	-2.277382	0.297007	H	-4.244665	0.31236	-1.470127	H	-4.17514	0.112667	-1.707975
**4d5**				**4d6**							
C	-1.856463	0.313866	0.130108	C	-1.838852	-0.163258	-0.075752				
C	-1.642802	1.810997	0.110612	C	-1.676073	1.149637	-0.808511				
C	-0.456494	2.363519	-0.694235	C	-0.866696	2.187804	0.000625				
C	0.949575	2.100645	-0.126155	C	0.660666	2.081761	-0.152895				
C	1.338709	0.650282	-0.155633	C	1.221457	0.678655	-0.125701				
C	0.560385	-0.46522	-0.062254	C	0.637673	-0.524378	0.143789				
C	-0.879191	-0.622191	0.056964	C	-0.762945	-0.856719	0.364066				
C	2.753241	0.26285	-0.319704	C	2.653628	0.460903	-0.443935				
C	2.827511	-1.256615	-0.42263	C	2.963833	-1.024821	-0.332586				
C	1.414628	-1.74502	-0.044885	C	1.660269	-1.679866	0.144944				
C	1.387884	-2.410358	1.342091	C	1.797071	-2.333756	1.528188				
O	0.853566	-2.638625	-1.023244	O	1.304852	-2.675799	-0.83411				

C	1.1536	2.701016	1.282177	C	1.351444	2.971897	0.90689
C	-3.324996	-0.094299	0.233801	C	-3.242341	-0.691022	0.172757
C	-3.999999	-0.024189	-1.156397	C	-4.066732	0.25686	1.069129
O	3.709305	1.025818	-0.385527	O	3.458981	1.323613	-0.769308
C	-3.588217	-1.461286	0.882497	C	-3.985735	-0.98607	-1.1467
H	-2.557445	2.27541	-0.278859	H	-1.170995	0.969152	-1.768537
H	-1.582495	2.157201	1.154045	H	-2.658574	1.56246	-1.053744
H	-0.506564	1.972076	-1.717785	H	-1.160618	3.199542	-0.304369
H	-0.58696	3.450589	-0.771296	H	-1.13653	2.097633	1.060062
H	1.662822	2.615566	-0.78368	H	0.935806	2.493351	-1.135145
H	-1.191245	-1.661397	0.074827	H	-0.953865	-1.798538	0.87917
H	3.629553	-1.652151	0.208387	H	3.811983	-1.182698	0.340961
H	3.048563	-1.523981	-1.461814	H	3.241919	-1.417856	-1.315112
H	0.365908	-2.667667	1.634034	H	0.849936	-2.76856	1.866283
H	1.986411	-3.330201	1.331773	H	2.5389	-3.13655	1.47751
H	1.810152	-1.745371	2.102161	H	2.11871	-1.602671	2.277393
H	1.295396	-3.495272	-0.948413	H	0.416958	-3.007104	-0.640538
H	2.197961	2.601956	1.591132	H	2.430707	3.005042	0.7486
H	0.899739	3.76793	1.282	H	0.963346	3.995115	0.848705
H	0.531108	2.20021	2.031114	H	1.153215	2.593397	1.916678
H	-3.809681	0.664691	0.866776	H	-3.13869	-1.642183	0.711338
H	-5.078118	-0.199064	-1.068383	H	-5.052722	-0.176906	1.268847
H	-3.858031	0.950073	-1.635206	H	-3.570717	0.427902	2.030053
H	-3.581829	-0.788908	-1.819973	H	-4.226549	1.230324	0.592483
H	-4.664795	-1.601489	1.027641	H	-4.969921	-1.41936	-0.937152
H	-3.100512	-1.544506	1.859168	H	-3.427428	-1.694321	-1.767515
H	-3.237127	-2.287213	0.254367	H	-4.145535	-0.07544	-1.734478

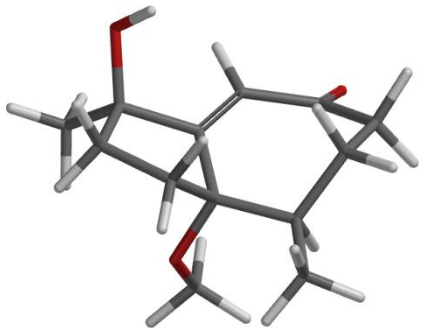

5a1 (42.67%)

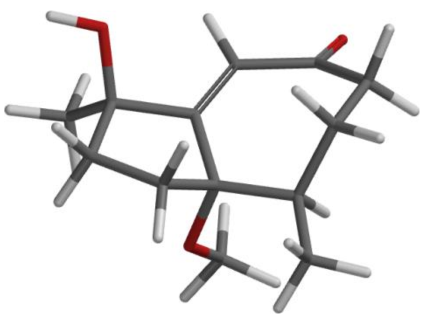

5a2 (20.15%)

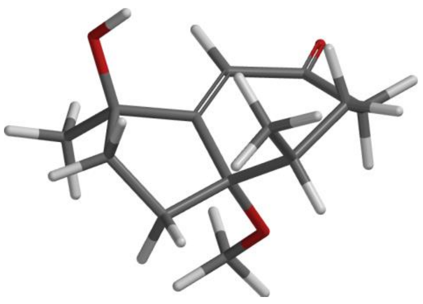

5a3 (15.04%)

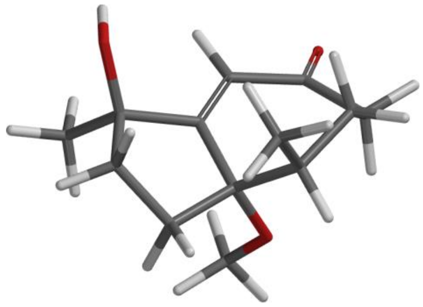

5a4 (8.51%)

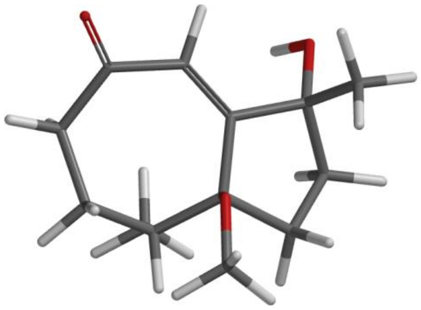

5a5 (6.20%)

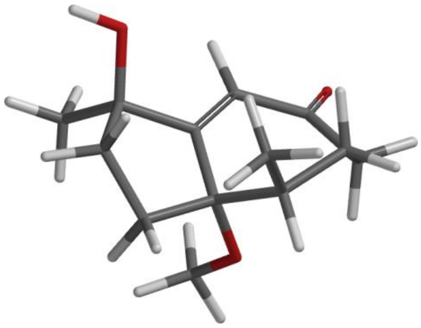

5a6 (3.92%)

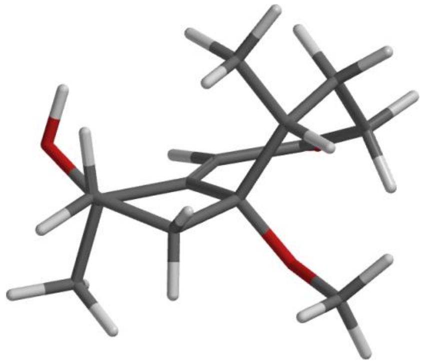

5a7 (3.51%)

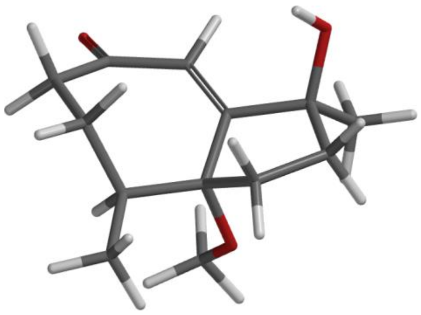

5b1 (46.10%)

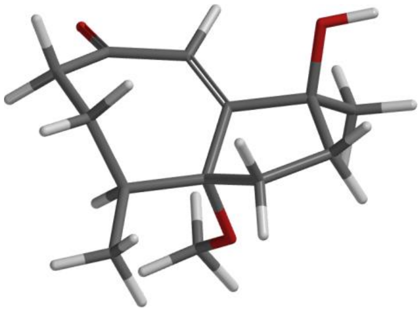

5b2 (21.76%)

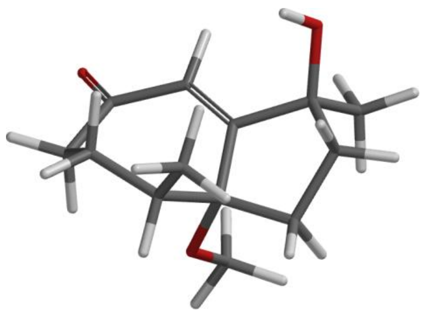

5b3 (16.24%)

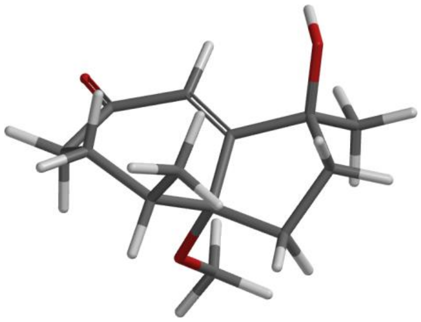

5b4 (9.19%)

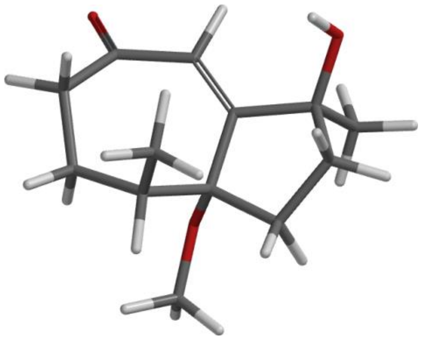

5b5 (6.70%)

Figure S88. Stable conformers of compound **5** with 1*R*,4*S*,10*S* (**5a**) and 1*S*,4*R*,10*R* (**5b**) configurations, respectively

Table S9. Important thermodynamic parameters (a.u.) of the optimized compound **5** at B3LYP/6-31G(d,p) level in the gas phase

conformations	E+ZPE	G	conformations	E+ZPE	G
**5a1**	-732.804167	-732.846759	**5b1**	-732.804167	-732.846759
**5a2**	-732.803559	-732.846051	**5b2**	-732.803559	-732.846051
**5a3**	-732.804229	-732.845775	**5b3**	-732.804229	-732.845775
**5a4**	-732.803464	-732.845238	**5b4**	-732.803464	-732.845238
**5a5**	-732.803226	-732.844940	**5b5**	-732.803226	-732.844940
**5a6**	-732.802921	-732.844506			
**5a7**	-732.802532	-732.844404			

Table S10. Optimized Z-Matrixes of compound **5** in the Gas Phase (Å) at B3LYP/6-31G (d,p) level

5a1				5a2				5a3			
C	-1.651092	1.884574	0.162693	C	-1.632615	1.890631	0.155124	C	2.023927	-1.302607	-0.455465
C	-2.653214	1.012281	-0.589128	C	-2.628830	1.042692	-0.630744	C	2.715076	0.019716	-0.157333
C	-2.146392	-0.306324	-1.182041	C	-2.124672	-0.281789	-1.213490	C	2.037136	1.243337	-0.794197
C	-1.471504	-1.208531	-0.128233	C	-1.483240	-1.189759	-0.143272	C	0.776789	1.829094	-0.110118
C	0.042752	-0.927710	0.022720	C	0.031869	-0.928466	0.030537	C	-0.122481	0.768743	0.614151
C	0.508537	0.526444	-0.063579	C	0.513590	0.518993	-0.063123	C	-0.368426	-0.481787	-0.235394
C	-0.187379	1.673889	0.008908	C	-0.169126	1.672168	0.005821	C	0.554666	-1.351287	-0.682865
C	0.946772	-1.583548	-1.042259	C	0.944379	-1.603609	-1.013532	C	-1.556250	1.281999	0.931645
C	2.317213	-0.904659	-0.857223	C	2.319152	-0.945849	-0.797836	C	-2.452561	0.738874	-0.190161
C	2.026980	0.535837	-0.335865	C	2.026285	0.511340	-0.333881	C	-1.867947	-0.658766	-0.514999
C	2.862488	0.897762	0.894467	C	2.860414	0.920881	0.887699	C	-2.488319	-1.752811	0.364952
O	2.361531	1.544879	-1.305351	O	2.239868	1.460832	-1.398718	O	-2.134930	-1.083470	-1.854681
O	0.541150	-1.490922	1.266889	O	0.503959	-1.487177	1.289640	O	0.621201	0.512138	1.827175
C	0.020609	-0.983709	2.491317	C	-0.033002	-0.964921	2.499970	C	0.227425	-0.564337	2.667498
C	-1.749241	-2.701037	-0.377128	C	-1.777374	-2.679275	-0.390225	C	0.054961	2.715537	-1.138603
O	-2.052688	2.836798	0.821952	O	-2.034359	2.829376	0.833457	O	2.671835	-2.339995	-0.555378
H	-3.487533	0.829533	0.099069	H	-3.488341	0.874352	0.029496	H	2.780862	0.136181	0.929309
H	-3.059633	1.657458	-1.380354	H	-2.994528	1.698683	-1.433067	H	3.737099	-0.088130	-0.534417
H	-1.465743	-0.116419	-2.021483	H	-1.422147	-0.097540	-2.035146	H	1.789741	0.994774	-1.835445
H	-3.007356	-0.834195	-1.608646	H	-2.982006	-0.800137	-1.658874	H	2.773653	2.053461	-0.854713
H	-1.935712	-0.957087	0.834073	H	-1.960619	-0.927952	0.809826	H	1.103805	2.472412	0.717433
H	0.373866	2.607561	-0.019397	H	0.394905	2.602186	-0.040651	H	0.223990	-2.235343	-1.225919
H	0.988725	-2.666769	-0.908285	H	0.966391	-2.687324	-0.877950	H	-1.875153	0.867819	1.892680
H	0.534887	-1.383573	-2.039072	H	0.564517	-1.389665	-2.018841	H	-1.591476	2.368687	1.037846
H	2.901603	-0.857350	-1.780479	H	2.927078	-0.938183	-1.707245	H	-2.384460	1.369208	-1.083495
H	2.907725	-1.455861	-0.121216	H	2.872194	-1.485092	-0.024211	H	-3.509793	0.682533	0.085955
H	2.622835	1.910211	1.232313	H	2.625956	1.947514	1.184962	H	-1.996762	-2.711326	0.178294
H	3.925266	0.861729	0.633709	H	3.931218	0.860767	0.651691	H	-3.550566	-1.848743	0.119519
H	2.674242	0.193306	1.707517	H	2.667400	0.253995	1.731891	H	-2.394760	-1.514895	1.427635
H	1.806253	1.427127	-2.088435	H	3.193079	1.582034	-1.509051	H	-1.673089	-0.496447	-2.469350
H	0.685131	-1.354414	3.275524	H	0.611571	-1.338221	3.299701	H	0.937713	-0.566131	3.497178
H	-0.993487	-1.351164	2.694709	H	-1.055456	-1.317649	2.687424	H	0.278439	-1.531169	2.152692
H	0.015183	0.113049	2.517606	H	-0.025682	0.132171	2.518240	H	-0.782009	-0.429895	3.078926
H	-1.218938	-3.330654	0.343406	H	-1.268456	-3.312992	0.342204	H	-0.775227	3.284293	-0.712324
H	-2.821180	-2.904336	-0.281664	H	-2.853621	-2.867070	-0.312638	H	0.765227	3.440303	-1.550756
H	-1.445258	-3.005809	-1.384696	H	-1.460187	-2.992353	-1.391082	H	-0.326068	2.128793	-1.982637
**5a4**				**5a5**				**5a6**			
C	2.039543	-1.261903	-0.482139	C	1.787149	1.694208	-0.357168	C	2.020407	-1.287905	-0.478340
C	2.711268	0.079954	-0.229331	C	2.689776	0.745418	0.416839	C	2.712904	0.040753	-0.210848

C	1.977562	1.283270	-0.843913	C	2.422157	-0.764443	0.301006	C	2.007519	1.257884	-0.829812
C	0.732968	1.842031	-0.110597	C	1.074429	-1.291648	0.835441	C	0.763086	1.834382	-0.109949
C	-0.116564	0.755421	0.631528	C	-0.132074	-0.906196	-0.066699	C	-0.112113	0.761826	0.624842
C	-0.365560	-0.484637	-0.231430	C	-0.496875	0.582901	-0.055319	C	-0.367926	-0.476032	-0.238672
C	0.567333	-1.340711	-0.680753	C	0.308933	1.646225	-0.193112	C	0.550565	-1.338900	-0.702861
C	-1.546575	1.226701	1.014363	C	-1.457330	-1.616938	0.370444	C	-1.539794	1.260780	0.984610
C	-2.475273	0.692425	-0.084204	C	-2.373511	-0.517680	0.929107	C	-2.457082	0.732740	-0.128254
C	-1.868484	-0.663912	-0.500862	C	-2.012521	0.739223	0.107886	C	-1.864732	-0.648305	-0.505126
C	-2.448995	-1.832583	0.312727	C	-2.724657	0.773264	-1.251574	C	-2.462541	-1.778412	0.352564
O	-2.160116	-0.849063	-1.891555	O	-2.390163	1.955684	0.752138	O	-2.035216	-0.961749	-1.894018
O	0.677383	0.490500	1.811502	O	0.168722	-1.183925	-1.457696	O	0.661135	0.493440	1.817523
C	0.328006	-0.599357	2.653478	C	0.406667	-2.537384	-1.821325	C	0.292019	-0.594856	2.652471
C	-0.047753	2.716210	-1.106186	C	0.857210	-0.944276	2.318510	C	0.008475	2.719218	-1.116503
O	2.704489	-2.288930	-0.580821	O	2.272965	2.591991	-1.035782	O	2.668052	-2.327219	-0.559777
H	2.827313	0.207230	0.851802	H	3.711216	0.961660	0.090732	H	2.817223	0.161318	0.872368
H	3.716500	-0.009619	-0.653999	H	2.621649	1.053402	1.470681	H	3.722117	-0.063965	-0.622507
H	1.691610	1.023674	-1.872031	H	3.218506	-1.275844	0.856981	H	1.729741	1.005730	-1.861930
H	2.691717	2.110111	-0.937511	H	2.536311	-1.059559	-0.746190	H	2.735561	2.073862	-0.911947
H	1.077370	2.484283	0.710901	H	1.137531	-2.388023	0.783959	H	1.107562	2.473166	0.714255
H	0.265878	-2.241376	-1.216020	H	-0.159801	2.626537	-0.271941	H	0.221267	-2.209067	-1.266810
H	-1.816766	0.784394	1.978005	H	-1.920081	-2.063630	-0.514159	H	-1.833664	0.828732	1.946018
H	-1.602499	2.309954	1.144561	H	-1.285491	-2.427312	1.084872	H	-1.579199	2.345586	1.107991
H	-2.471279	1.344419	-0.961647	H	-2.146129	-0.325372	1.985838	H	-2.412010	1.373370	-1.013204
H	-3.514030	0.588772	0.243456	H	-3.437820	-0.762781	0.862351	H	-3.506048	0.663187	0.180744
H	-1.957791	-2.775941	0.047455	H	-2.409377	1.659699	-1.808097	H	-1.974411	-2.729578	0.122552
H	-3.520313	-1.921430	0.106546	H	-3.807015	0.821754	-1.094607	H	-3.537485	-1.873501	0.151309
H	-2.311948	-1.682287	1.388164	H	-2.484656	-0.108728	-1.849674	H	-2.344539	-1.580403	1.421860
H	-1.855843	-1.723494	-2.170907	H	-1.923103	2.020108	1.597106	H	-2.950827	-1.236570	-2.039441
H	1.057728	-0.590385	3.466126	H	0.471137	-2.546256	-2.911771	H	1.017749	-0.600771	3.468772
H	0.388802	-1.562191	2.131984	H	-0.408752	-3.205191	-1.513282	H	0.340747	-1.555763	2.126255
H	-0.674041	-0.491050	3.089884	H	1.350092	-2.923576	-1.412675	H	-0.710483	-0.473286	3.085271
H	-0.865622	3.272084	-0.639754	H	-0.008475	-1.466810	2.736570	H	-0.808309	3.287135	-0.662785
H	0.632214	3.453631	-1.546327	H	1.731512	-1.244387	2.906408	H	0.704974	3.446693	-1.547323
H	-0.457904	2.121708	-1.929778	H	0.704436	0.128282	2.473404	H	-0.396463	2.129307	-1.946003
**5a7**				**5b1**				**5b2**			
C	1.801999	1.722435	-0.133359	C	1.651092	1.884574	0.162693	C	1.632615	1.890631	0.155124
C	2.642813	0.472440	0.079824	C	2.653214	1.012281	-0.589128	C	2.628830	1.042692	-0.630744
C	2.108444	-0.453121	1.186251	C	2.146392	-0.306324	-1.182041	C	2.124672	-0.281789	-1.213490
C	0.910129	-1.385505	0.867135	C	1.471504	-1.208531	-0.128233	C	1.483240	-1.189759	-0.143272
C	-0.063011	-0.853568	-0.238049	C	-0.042752	-0.927710	0.022720	C	-0.031869	-0.928466	0.030537
C	-0.477866	0.601838	-0.020889	C	-0.508537	0.526444	-0.063579	C	-0.513590	0.518993	-0.063123

C	0.325876	1.678008	0.024240	C	0.187379	1.673889	0.008908	C	0.169126	1.672168	0.005821
C	-1.422263	-1.616549	-0.296775	C	-0.946772	-1.583548	-1.042259	C	-0.944379	-1.603609	-1.013532
C	-2.433672	-0.726275	0.439466	C	-2.317213	-0.904659	-0.857223	C	-2.319152	-0.945849	-0.797836
C	-2.003578	0.714651	0.080486	C	-2.026980	0.535837	-0.335865	C	-2.026285	0.511340	-0.333881
C	-2.607470	1.173244	-1.254885	C	-2.862488	0.897762	0.894467	C	-2.860414	0.920881	0.887699
O	-2.436731	1.687621	1.033218	O	-2.361531	1.544879	-1.305351	O	-2.239868	1.460832	-1.398718
O	0.588586	-0.877252	-1.532742	O	-0.541150	-1.490922	1.266889	O	-0.503959	-1.487177	1.289640
C	0.929989	-2.142031	-2.083291	C	-0.020609	-0.983709	2.491317	C	0.033002	-0.964921	2.499970
C	0.231292	-1.749392	2.200457	C	1.749241	-2.701037	-0.377128	C	1.777374	-2.679275	-0.390225
O	2.336042	2.794565	-0.399733	O	2.052688	2.836798	0.821952	O	2.034359	2.829376	0.833457
H	2.717276	-0.053113	-0.877226	H	3.487533	0.829533	0.099069	H	3.488341	0.874352	0.029496
H	3.645558	0.826655	0.337486	H	3.059633	1.657458	-1.380354	H	2.994528	1.698683	-1.433067
H	1.842933	0.173325	2.048479	H	3.007356	-0.834195	-1.608646	H	2.982006	-0.800137	-1.658874
H	2.931068	-1.090360	1.532879	H	1.465743	-0.116419	-2.021483	H	1.422147	-0.097540	-2.035146
H	1.311033	-2.321720	0.457924	H	1.935712	-0.957087	0.834073	H	1.960619	-0.927952	0.809826
H	-0.129710	2.662205	0.119554	H	-0.373866	2.607561	-0.019397	H	-0.394905	2.602186	-0.040651
H	-1.711502	-1.699495	-1.349312	H	-0.988725	-2.666769	-0.908285	H	-0.966391	-2.687324	-0.877950
H	-1.370124	-2.631882	0.104614	H	-0.534887	-1.383573	-2.039072	H	-0.564517	-1.389665	-2.018841
H	-2.361225	-0.863462	1.524613	H	-2.901603	-0.857350	-1.780479	H	-2.927078	-0.938183	-1.707245
H	-3.470902	-0.926985	0.154945	H	-2.907725	-1.455861	-0.121216	H	-2.872194	-1.485092	-0.024211
H	-2.242275	2.172841	-1.503601	H	-2.622835	1.910211	1.232313	H	-2.625956	1.947514	1.184962
H	-3.698144	1.206963	-1.166984	H	-3.925266	0.861729	0.633709	H	-3.931218	0.860767	0.651691
H	-2.334499	0.495514	-2.067981	H	-2.674242	0.193306	1.707517	H	-2.667400	0.253995	1.731891
H	-1.996403	1.518548	1.877761	H	-1.806253	1.427127	-2.088435	H	-3.193079	1.582034	-1.509051
H	1.217037	-1.948032	-3.119488	H	-0.685131	-1.354414	3.275524	H	-0.611571	-1.338221	3.299701
H	0.085588	-2.843617	-2.080113	H	-0.015183	0.113049	2.517606	H	0.025682	0.132171	2.518240
H	1.780282	-2.611314	-1.569745	H	0.993487	-1.351164	2.694709	H	1.055456	-1.317649	2.687424
H	-0.535240	-2.521763	2.097535	H	1.218938	-3.330654	0.343406	H	1.268456	-3.312992	0.342204
H	0.984598	-2.130275	2.898552	H	2.821180	-2.904336	-0.281664	H	2.853621	-2.867070	-0.312638
H	-0.224624	-0.870559	2.670496	H	1.445258	-3.005809	-1.384696	H	1.460187	-2.992353	-1.391082
**5b3**				**5b4**				**5b5**			
C	-2.023927	-1.302607	-0.455465	C	-2.039543	-1.261903	-0.482139	C	-1.787149	1.694208	-0.357168
C	-2.715076	0.019716	-0.157333	C	-2.711268	0.079954	-0.229331	C	-2.689776	0.745418	0.416839
C	-2.037136	1.243337	-0.794197	C	-1.977562	1.283270	-0.843913	C	-2.422157	-0.764443	0.301006
C	-0.776789	1.829094	-0.110118	C	-0.732968	1.842031	-0.110597	C	-1.074429	-1.291648	0.835441
C	0.122481	0.768743	0.614151	C	0.116564	0.755421	0.631528	C	0.132074	-0.906196	-0.066699
C	0.368426	-0.481787	-0.235394	C	0.365560	-0.484637	-0.231430	C	0.496875	0.582901	-0.055319
C	-0.554666	-1.351287	-0.682865	C	-0.567333	-1.340711	-0.680753	C	-0.308933	1.646225	-0.193112
C	1.556250	1.281999	0.931645	C	1.546575	1.226701	1.014363	C	1.457330	-1.616938	0.370444
C	2.452561	0.738874	-0.190161	C	2.475273	0.692425	-0.084204	C	2.373511	-0.517680	0.929107
C	1.867947	-0.658766	-0.514999	C	1.868484	-0.663912	-0.500862	C	2.012521	0.739223	0.107886

C	2.488319	-1.752811	0.364952	C	2.448995	-1.832583	0.312727	C	2.724657	0.773264	-1.251574
O	2.134930	-1.083470	-1.854681	O	2.160116	-0.849063	-1.891555	O	2.390163	1.955684	0.752138
O	-0.621201	0.512138	1.827175	O	-0.677383	0.490500	1.811502	O	-0.168722	-1.183925	-1.457696
C	-0.227425	-0.564337	2.667498	C	-0.328006	-0.599357	2.653478	C	-0.406667	-2.537384	-1.821325
C	-0.054961	2.715537	-1.138603	C	0.047753	2.716210	-1.106186	C	-0.857210	-0.944276	2.318510
O	-2.671835	-2.339995	-0.555378	O	-2.704489	-2.288930	-0.580821	O	-2.272965	2.591991	-1.035782
H	-2.780862	0.136181	0.929309	H	-2.827313	0.207230	0.851802	H	-3.711216	0.961660	0.090732
H	-3.737099	-0.088130	-0.534417	H	-3.716500	-0.009619	-0.653999	H	-2.621649	1.053402	1.470681
H	-2.773653	2.053461	-0.854713	H	-2.691717	2.110111	-0.937511	H	-2.536311	-1.059559	-0.746190
H	-1.789741	0.994774	-1.835445	H	-1.691610	1.023674	-1.872031	H	-3.218506	-1.275844	0.856981
H	-1.103805	2.472412	0.717433	H	-1.077370	2.484283	0.710901	H	-1.137531	-2.388023	0.783959
H	-0.223990	-2.235343	-1.225919	H	-0.265878	-2.241376	-1.216020	H	0.159801	2.626537	-0.271941
H	1.875153	0.867819	1.892679	H	1.816766	0.784394	1.978005	H	1.920081	-2.063630	-0.514159
H	1.591476	2.368687	1.037846	H	1.602499	2.309954	1.144561	H	1.285491	-2.427312	1.084872
H	2.384460	1.369208	-1.083495	H	2.471279	1.344419	-0.961647	H	2.146129	-0.325372	1.985838
H	3.509793	0.682533	0.085955	H	3.514030	0.588772	0.243456	H	3.437820	-0.762781	0.862351
H	1.996762	-2.711326	0.178294	H	1.957791	-2.775941	0.047455	H	2.409377	1.659699	-1.808097
H	3.550566	-1.848743	0.119519	H	3.520313	-1.921430	0.106546	H	3.807015	0.821754	-1.094607
H	2.394760	-1.514895	1.427635	H	2.311948	-1.682287	1.388164	H	2.484656	-0.108728	-1.849674
H	1.673089	-0.496447	-2.469350	H	1.855843	-1.723494	-2.170907	H	1.923103	2.020108	1.597106
H	-0.937713	-0.566131	3.497178	H	-1.057728	-0.590385	3.466126	H	-0.471137	-2.546256	-2.911771
H	0.782009	-0.429895	3.078926	H	0.674041	-0.491050	3.089884	H	-1.350092	-2.923576	-1.412675
H	-0.278439	-1.531169	2.152692	H	-0.388802	-1.562191	2.131984	H	0.408752	-3.205191	-1.513282
H	0.775227	3.284293	-0.712324	H	0.865622	3.272084	-0.639754	H	0.008475	-1.466810	2.736570
H	-0.765227	3.440303	-1.550756	H	-0.632214	3.453631	-1.546327	H	-1.731512	-1.244387	2.906408
H	0.326068	2.128793	-1.982637	H	0.457904	2.121708	-1.929778	H	-0.704436	0.128282	2.473404
